# Performance Comparison of Different Constructed Wetlands Designs for the Removal of Personal Care Products

**DOI:** 10.3390/ijerph17093091

**Published:** 2020-04-29

**Authors:** Huma Ilyas, Eric D. van Hullebusch

**Affiliations:** 1 Institut de physique du globe de Paris, Université de Paris, CNRS, F-75005 Paris, France; vanhullebusch@ipgp.fr; 2 Water Treatment and Management Consultancy, B.V., 2289 ED Rijswijk, The Netherlands

**Keywords:** artificial aeration, constructed wetlands, personal care products, physicochemical properties, removal efficiency, removal mechanisms

## Abstract

This research investigates the performance of four types of constructed wetlands (CWs): free water surface CW (FWSCW), horizontal flow CW (HFCW), vertical flow CW (VFCW), and hybrid CW (HCW) for the removal of 20 personal care products (PCPs), based on secondary data compiled for 137 CWs reported in 39 peer reviewed journal papers. In spite of considerable variation in the re-moval efficiency of PCPs, CWs prove to be a promising treatment technology. The average removal efficiency of 15 widely studied PCPs ranged from 9.0% to 84%. Although CWs effectively reduced the environmental risks caused by many PCPs, triclosan was still classified under high risk category based on effluent concentration. Five other PCPs were classified under medium risk category (triclocarban > methylparaben > galaxolide > oxybenzone > methyl dihydrojasmonate). In most of the examined PCPs, adsorption and/or sorption is the most common removal mechanism followed by biodegradation and plant uptake. The comparatively better performance of HCW followed by VFCW, HFCW, and FWSCW might be due to the co-existence of aerobic and anaerobic conditions, and longer hydraulic retention time enhancing the removal of PCPs (e.g., triclosan, methyl dihydro-jasmonate, galaxolide, tonalide, and oxybenzone), which are removed under both conditions and by adsorption/sorption processes.

## 1. Introduction

Personal care products (PCPs) are among the emerging organic contaminants (EOCs) that are discharged to water resources and environment through various sources such as domestic wastewater (from bathing, shaving, spraying, swimming etc.), industrial wastewater (from product manufacturing discharges), landfill leachate (from improper disposal of used, defective or expired items), and effluent discharge from wastewater treatment plants (WWTPs) [1,2,3,4,5,6,7,8]. Although PCPs are often found in very low concentrations (e.g., ng L^−1^ to µg L^−1^) in water bodies, they can still pose negative impacts on human health as well as aquatic and terrestrial life, if these are discharged continuously through various sources including WWTPs [6]. It has been indicated that higher concentration of PCPs compared with their potential no-effect concentration could pose severe risk to human health, since many of these PCPs are considered as prospective endocrine disruptors [3,9].

Constructed wetlands (CWs) are cost-effective and nature-based treatment technologies that have been comprehensively investigated for the removal of PCPs from wastewater over the last decade [10,11,12,13]. More than 50 individual case studies related to PCPs have been published in peer reviewed journals including the investigation of free water surface CW (FWSCW), horizontal flow CW (HFCW), vertical flow CW (VFCW), and hybrid CW (HCW). Most of the studies focus on one or more topics such as different designs, impact of seasonality, and physicochemical properties of PCPs. An in-depth examination of these studies revealed that the research is lacking on a comparative assessment of performance by different types of CWs. Although comparative analysis on the performance of different types of CWs has been conducted within the individual studies (also a limited number), it has not been done between the studies. For instance, a comparison was made between FWSCW, HFCW, and HCW [14,15,16]; between HFCW, VFCW, and HCW [17,18]; between FWSCW and VFCW [19]; and between VFCW and HFCW [20]. However, a comprehensive and critical review of performance and a comparison of all types of CWs is lacking. Furthermore, most of the studies examine only a limited number of PCPs (Tables S1–S4, Supplementary Materials). Additionally, a comprehensive statistical analysis is missing, for instance, a meta-analysis of existing studies to ascertain significant differences in the performance of different types of CWs. Some recent studies investigated the effect of dissolved oxygen (DO) by the application of artificial aeration (AA) in CWs; for example, in FWSCW [21]; HFCW [17,18]; and VFCW [17,18,22] (Tables S1–S3, Supplementary Materials).

Although, based on a large number of research studies, it is possible to critically evaluate and summarize the available knowledge of the indicated areas, only a few studies reviewed and summarized the existing knowledge on some of the above mentioned topics with specific focus on PCPs removal by CWs [10,11,12,13]. Verlicchi and Zambello [10] and Verlicchi et al. [12] analyzed the performance of CWs used for primary, secondary, and tertiary treatment. The authors also discussed the role of physicochemical properties of PCPs on their removal efficiencies. Verlicchi and Zambello [10] only studied triclosan and triclocarban. However, Verlicchi et al. [12] studied triclosan, triclocarban, tonalide, methyl dihydrojasmonate, cashmeran, celestolide, oxybenzone, hydrocinnamic acid, and N,N-diethyl-3-methyl benzoylamide. A scientific elucidation of removal processes in CWs has been presented by Zhang et al. [11] and Vo et al. [13], which was supported by (limited) scientific evidence from available studies, and the few PCPs considered by both studies were triclosan, tonalide, galaxolide, methyl dihydrojasmonate, and oxybenzone. In spite of significant contribution of these review studies in advancing scientific knowledge of the removal of PCPs by CWs, comprehensive and critical research is needed to infer evidence based general conclusions. Furthermore, most of the previous reviews were constrained by a limited number of existing studies on certain topics and also considered a limited number of PCPs (from two to nine).

Therefore, the main objectives of this study are: (1) to critically evaluate and summarize the available evidence on major PCPs removal mechanisms; (2) to investigate the impact of physicochemical properties of PCPs on their removal processes; (3) to assess the environmental risk posed by PCPs and the contribution of CWs in risk reduction; (4) to conduct a comparative assessment of four types of CWs for the removal of large number of PCPs; and (5) to analyze the effect of AA on the removal of PCPs in different types of CWs.

## 2. Methods

This research is based on the secondary data and a critical review of the published literature. The research papers, review papers, and books were searched in various sources, such as Scopus, Google Scholar, and individual journal websites, related to the performance of different types of CWs for the removal of different categories of PCPs. The snowball sampling method yielded over 50 journal articles published up to 2019, which were further screened and used for the purpose of this research. Screening was carried out to check the quality of the published data. Only peer-reviewed journal papers were selected for this research, which helped to ensure the reliability of given data. The selected studies have used generally accepted and reliable analytical methods such as solid phase extraction-gas chromatography-tandem mass spectrometry (SPE-GC-MS/MS); SPE-(ultra) high performance liquid chromatography-MS/MS (SPE-(U)HPLC-MS/MS); and liquid-liquid phase extraction-(U)HPLC-MS/MS (LLPE-(U)HPLC-MS/MS). Instrumental detection and quantification limits described as limit of detection and limit of quantification were in the range of 0.0002–1.0 μg L^−1^ and 0.00005–0.8 μg L^−1^, respectively. The samples were analyzed soon after collection, as the storage time was less than one or two days in most cases. The selected studies contained the required information on most of the key parameters such as concentration of PCPs in influent and effluent waters, removal efficiency, biochemical oxygen demand (BOD), chemical oxygen demand (COD), hydraulic loading rate (HLR), and hydraulic retention time (HRT). In this way, a global database was compiled containing information on 137 CWs that were reported in 39 journal publications with case studies from 13 countries (Tables S1–S4, Supplementary Materials). This database contains influent and effluent concentrations, removal efficiencies and rates of 20 PCPs grouped in seven categories according to their uses (Table 1).

The treatment performance of four types of CWs, namely, FWSCW, HFCW, VFCW, and HCW was evaluated for the removal of selected PCPs with three or more data points. The other parameters such as treatment scale and type, wastewater type, depth, area, HLR, organic loading rate (OLR), HRT, experiment duration, system age, filter media, temperature, pH, DO, and oxidation reduction potential (ORP) were considered for the comparison of four types of CWs. The removal mechanisms were identified for the selected PCPs as presented in the published case studies. The majority of the studies only attributed removal to certain mechanisms (e.g., biodegradation, adsorption/sorption, plant uptake, and photodegradation). The relative contribution of mechanisms to removal was only quantified in a few experimental studies (Table S5, Supplementary Materials). Therefore, the analysis of removal mechanisms was based on a critical oversight of both qualitative and quantitative information. The information on the physicochemical properties of PCPs was gathered from various sources (e.g., journal papers, reports, and websites) for molecular formula/structure/weight, water solubility, dissociation constant (pKa), organic carbon sorption coefficient (Log Koc), octanol-water partition coefficient (Log Kow), and distribution coefficient (Log Dow). The available evidence on the role of these properties in the removal of PCPs in CWs was comprehensively and critically analyzed. The linkages between physicochemical properties and removal mechanisms were delineated from this analysis. Additionally, risk assessment was carried out by estimating risk quotient (RQ), which is a ratio between the predicted or measured environmental concentrations (PEC or MEC), and the worst-case predicted no effect concentration (PNEC) [23]. Following the recommendations by Hernando et al. [23] and several applications [24,25,26,27,28,29,30,31], we categorized the risk into four levels: high risk (RQ > 1.0), medium risk (0.1 ≤ RQ ≤ 1.0), low risk (0.01 ≤ RQ ≤ 0.1), and no risk (RQ < 0.01).

Furthermore, statistical analysis was conducted to estimate mean and standard deviation of the selected studied variables. The statistical comparison among different types of CWs was performed with one-way ANOVA for significance and z-Test for pair-wise comparison of means between two types of CWs.

## 3. Results and Discussion

### 3.1. Removal of Widely Studied PCPs by CWs

**Figure 1:**
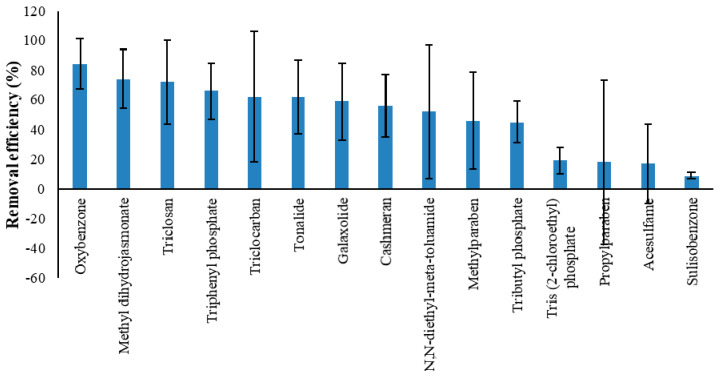
Removal efficiency of widely investigated PCPs. Note: the statistics are for 15 PCPs with three or more data points.

The average removal efficiencies of 15 out of 20 examined PCPs, for which three or more data points were available, are presented in Figure 1. The estimated statistics (mean and standard deviation of influent and effluent concentration, removal efficiency and rate) are given in Table S6 (Supplementary Materials).

The analysis presented in Figure 1 and Table S6 (Supplementary Materials) shows a very high range of variability in the influent and effluent concentrations and removal efficiencies among the studied PCPs. In spite of large variation, CWs are demonstrated as a promising wastewater treatment technology for a large number of PCPs. For instance, for nine out of 15 widely studied PCPs, CWs reveal a moderate to high potential for removal (e.g., average removal efficiency > 50%) in the case of oxybenzone (84%), methyl dihydrojasmonate (74%), triclosan (72%), triphenyl phosphate (66%), triclocarban (62%), tonalide (62%), galaxolide (59%), cashmeran (56%), and N,N-diethyl-meta-toluamide (52%). The four PCPs indicating lowest removal efficiency (average removal efficiency < 25%) are tris (2-chloroethyl) phosphate (19%), propylparaben (18%), acesulfame (17%), and sulisobenzone (9.0%).

### 3.2. Environmental Risk Assessment for the Selected PCPs

As previously stated, PCPs are discharged into water resources through various routes. Many of the PCPs are considered as prospective endocrine disruptors, and the higher concentration of PCPs compared with their PNEC could pose severe risk to human health as well as aquatic and terrestrial life [3,9]. Therefore, it is important to estimate the environmental risk posed by PCPs and the contribution of CWs in their risk reduction.

Environmental risk was evaluated for 11 PCPs for which the PNEC estimates were available in the literature. The PNEC value for a certain PCP is reported based on experimental and modeling studies related to *Daphnia magna* (e.g., references [25,28]), which was used to calculate RQ of that PCP in the influent and effluent water. PNEC values of selected PCPs show large variation in water. For example, the PNEC values of triclosan and triclocarban were below 0.5 μg L^−1^, which indicate the high toxicities of these compounds in the aqueous phase. On the other hand, the PNEC estimate of tris (2-chloroethyl) phosphate above 100 μg L^−1^ showed its comparatively lower toxicities in the aqueous phase [25] (Table 2).

**Figure 2:**
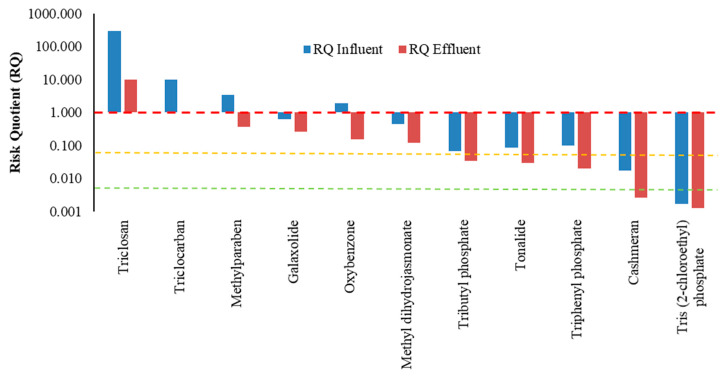
Risk quotient of the 11 selected PCPs based on influent and effluent concentration in constructed wetlands (CWs). Note: risk is categorized into four levels: high risk (RQ > 1.0; above red line), medium risk (0.1 ≤ RQ ≤ 1.0; between red and orange line), low risk (0.01 ≤ RQ ≤ 0.1; between orange and green line), and no risk (RQ < 0.01; below green line) (For interpretation of the references to colour in this figure legend, the reader is referred to the web version of this article).

The RQ was calculated using the PNEC value and the MEC of influent and effluent of PCPs. These calculations were performed for the selected PCPs based on all the available data points. The mean RQ were estimated from this analysis and discussed in detail in this section (Figure 2; Table 2). Since mean could be biased towards high values, median and various other percentiles were also estimated. The RQ was also estimated based on extremes (minimum and maximum values). The resulting statistics are given in Table S7 (Supplementary Materials). The results reveal that estimated RQs based on effluent concentrations are considerably lower compared with those based on influent values (Figure 2; Table 2), which indicated the significant role of CWs in attenuating the ecological risk posed by PCPs. These assessments are similar to the studies by Zhu and Chen [25]; Matamoros et al. [28,29]; and Chen et al. [27]. Although CWs do contribute to reducing ecological risk of several PCPs, this is not fully abolished in most of the cases (Table 2). Based on the effluent concentrations, cashmeran and tris (2-chloroethyl) phosphate could be classified as no risk PCPs, whereas triclosan is assessed as high-risk PCP, despite considerable risk reduction after treatment. The PCPs under medium risk category are triclocarban, methylparaben, galaxolide, oxybenzone, and methyl dihydrojasmonate. Tributyl phosphate, tonalide, and triphenyl phosphate pose low risk. The above mentioned findings agree with Matamoros et al. [29] and Chen et al. [27], as they also reported triclosan under high risk category. Matamoros et al. [29] depicted that galaxolide and oxybenzone had medium risk in effluent. The study by Zhu and Chen [25] indicated that triclocarban had a medium risk in effluent. However, contrary to our findings methylparaben (medium risk PCP) was designated under low risk category by Chen et al. [27]. These results indicated the need to include only a few PCPs (under high to medium risk category based on data from several countries) in regulatory monitoring and control purposes, as well as for water quality standard formulation purpose.

### 3.3. Role of Physicochemical Properties of PCPs and Removal Mechanisms in CWs

**Figure 3:**
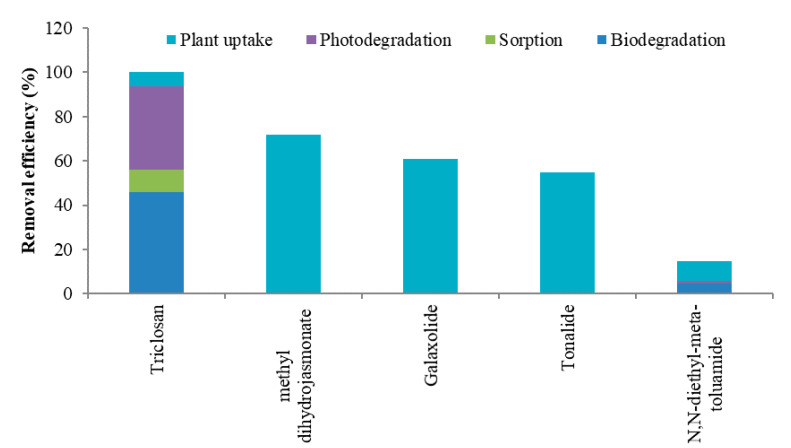
Relative contribution of removal mechanisms for PCPs in hydroponic microcosms, media adsorption experiments, and CWs. Note: Adsorption is the adhesion of dissolved solid molecules (adsorbate) to a surface of the substrate (adsorbent). It is a surface phenomenon. Absorption is a process in which a fluid (absorbate) permeates a solid (absorbent), thus involves the whole volume of the material. In the case of sorption both processes take place. Data is taken from: Hijosa-Valsero et al. [14,15]; Matamoros et al. [36]; Reyes-Contreras et al. [16]; and Li et al. [21]. The studies examined the contribution of one or more removal mechanisms. When the sum of reported contributions by different mechanisms exceeded 100%, we standardized the contribution from each mechanism to 100% by adding removal of all the studied mechanisms and dividing it by the total. For example, in the case of triclosan, the contribution by biodegradation, sorption, photodegradation, and plant uptake was 84%, 19%, 69%, and 11%, respectively. The total is 183%. However out of 100% the contribution of biodegradation, adsorption, photodegradation, and plant uptake was 46%, 10%, 38%, and 6.1%, respectively.

The available evidence suggests that the physicochemical properties of PCPs play a pivotal role in their removal processes in CWs (Table 3 and Table 4). For a few PCPs, experimental studies (e.g., hydroponic microcosms, media adsorption experiments, and CWs) were conducted to calculate the relative contribution of various mechanisms (Figure 3; Table S5, Supplementary Materials). The possible removal pathways of PCPs revealed by experimental studies are biodegradation (aerobic and anaerobic), plant uptake, adsorption, sorption, hydrolysis, volatilization, and photodegradation. Although for most of the PCPs more than one mechanism contributes to their removal, all the above mentioned removal pathways are not responsible for every PCP. Therefore, a compound specific examination is required for understanding each removal mechanism. Furthermore, it is noteworthy that environmental conditions in a certain type of CW (e.g., FWSCW, HFCW, and VFCW) are different in order to facilitate the specific removal mechanisms. For instance, HFCW and VFCW provide suitable conditions for the growth of microbial communities, which contribute to PCPs’ biodegradation, whereas, FWSCW enables the possibility of photodegradation of PCPs.

The physicochemical properties of EOCs which play a considerable role in the removal processes are governed by molecular weight/structure, solubility in water, Log Kow, Log Koc, Log Dow, cationic or anionic nature (pKa/charge), and presence of certain elements (e.g., chlorine, well known for its recalcitrance against biodegradation) (Table S8, Supplementary Materials). The removal of EOCs by plant uptake in CWs is considered an important mechanism. However, it is dominant only for few PCPs such as methylparaben, propylparaben, and methyl dihydrojasmonate (Table 3). The removal of these PCPs is low to moderate (Figure 1; Table S6, Supplementary Materials). Most of the studied PCPs showed their removal by biodegradation (aerobic and/or anaerobic) (Table 3), which indicates that the environmental conditions required for biodegradation must be ensured in the treatment systems, which aim to remove multiple types of PCPs. It should be acknowledged that for the readily biodegradable compounds, which mostly showed moderate to high removal (e.g., triclosan, methyl dihydrojasmonate, and oxybenzone) (Table 3, Table S6, Supplementary Materials), their biodegradability cannot be established only by physicochemical properties. The removal of most of the PCPs with Log Kow < 3.5 was attributed to biodegradation (Table 3 and Table 4), which indicates that experimental studies are indispensable to ascertain biodegradability of a certain PCP. The complexity of the biodegradation process itself (how much a compound is biodegradable) is evident by the low removal of PCPs (e.g., acesulfame, methylparaben, propylparaben, and sulisobenzone) (Table 3, Table S6, Supplementary Materials), although biodegradation is considered as their dominant removal pathway. When adsorption to the substrate and sorption onto organic surfaces are considered as the dominant removal mechanism, the compounds showed moderate or low removal (e.g., triclosan, triclocarban, cashmeran, galaxolide, tonalide, tributyl phosphate, and tris (2-chloroethyl) phosphate), even in the CWs that can provide media for adsorption and sorption (Table 3, Table S6, Supplementary Materials). The complication in the occurrence of adsorption and sorption processes in CWs might be due to the creation of biofilms around the filter media obstructing their access to adsorption/sorption surfaces [37,38]. The other process considerably contributing to the removal of some of the studied PCPs is photodegradation (e.g., methylparaben, propylparaben, triclosan, and tonalide), which also demonstrates low to moderate removal efficiencies of these PCPs (Table 3, Table S6, Supplementary Materials).

**Table 3:** Removal mechanisms of 15 widely studied PCPs in CWs.

Class/PCPs	Possible Removal Mechanism	References	Dominant Removal Mechanism *
**Artificial sweeteners**			
Acesulfame	Biodegradation (aerobic)	Kahl et al. [17]; Nivala et al. [18]	Biodegradation (aerobic)
**Preservatives**			
Methylparaben	Plant uptake	Anjos et al. [39]; Petrie et al. [40]	Plant uptake; Biodegradation (aerobic) Photodegradation **
	Biodegradation (aerobic)	Matamoros et al. [28,29]; Anjos et al. [39]; Chen et al. [27]	
	Photodegradation	Chen et al. [27]	
	Hydrolysis	Chen et al. [27]	
	Volatilization	Chen et al. [27]	
Propylparaben	Plant uptake	Anjos et al. [39]	Plant uptake; Biodegradation (aerobic) Photodegradation **
	Biodegradation (aerobic)	Anjos et al. [39]	
	Photodegradation	NA	
**Insect repellents**			
N,N-diethyl-meta- toluamide	Biodegradation (aerobic)	Li et al. [21]; Sgroi et al. [20]	Biodegradation (anaerobic) **
	Biodegradation (anaerobic)	Yi et al. [5]; Sgroi et al. [20]	
**Antiseptics**			
Triclosan	Adsorption	Carranza-Diaz et al. [41]; Chen et al. [26]; Liu et al. [42]; Xie et al. [43]; Button et al. [44]; Wang et al. [45]	Adsorption; Biodegradation (aerobic); Photodegradation
	Sorption	Ávila et al. [46]; Vystavna et al. [47]	
	Biodegradation (aerobic)	Ávila et al. [22,46,48]; Zhang et al. [11]; Zhao et al. [49]; Chen et al. [26]; Liu et al. [42]; Li et al. [21]; Vymazal et al. [31]; Xie et al. [43]; Button et al. [44]; Chen et al. [27]; Wang et al. [45]	
	Biodegradation (anaerobic)	Park et al. [50]; Vystavna et al. [47]	
	Photodegradation	Matamoros and Salvadó [51]; Zhang et al. [11]; Ávila et al. [46,48]; Matamoros et al. [28]; Li et al. [21]; Vymazal et al. [31]; Vystavna et al. [47]; Francini et al. [52]; Chen et al. [27]	
	Plant uptake	Zhang et al. [11]; Liu et al. [42]; Dai et al. [30]; Li et al. [21]; Vymazal et al. [31]; Francini et al. [52]; Xie et al. [43]	
Triclocarban	Sorption	Zhu and Chen [25]; Vymazal et al. [31]	Sorption **
**Fragrances**			
Methyl dihydro-jasmonate	Biodegradation (aerobic)	Matamoros et al. [19,28]; Hijosa-Valsero et al. [14,15,53,54]; Reyes-Contreras et al. [16]	Biodegradation (aerobic); Plant uptake
	Biodegradation (anaerobic)	Hijosa-Valsero et al. [14]	
	Plant uptake	Hijosa-Valsero et al. [14,15]; Reyes-Contreras et al. [16]; Salcedo et al. [55]	
	Retention processes	Hijosa-Valsero et al. [15]	
Cashmeran	Sorption	Matamoros and Salvadó [51]	Sorption **; Adsorption **
	Adsorption	NA	
Galaxolide	Plant uptake	Hijosa-Valsero et al. [14,15]; Reyes-Contreras et al. [16]; Salcedo et al. [55]	Sorption; Adsorption
	Adsorption	Hijosa-Valsero et al. [14,15]; Reyes-Contreras et al. [16]	
	Retention processes	Hijosa-Valsero et al. [15]	
	Sorption onto organic surfaces	Matamoros and Bayona [56]; Matamoros et al. [19,28]; Hijosa-Valsero et al. [14,54]; Matamoros and Salvadó [51]; Carranza-Diaz et al. [41]	
Tonalide	Plant uptake	Hijosa-Valsero et al. [14,15]; Reyes-Contreras et al. [16]	Sorption; Adsorption
	Adsorption	Hijosa-Valsero et al. [14,15]; Reyes-Contreras et al. [16]	
	Retention processes	Hijosa-Valsero et al. [15]	
	Sorption onto organic surfaces	Matamoros and Bayona [56]; Matamoros et al. [19,28]; Hijosa- Valsero et al. [14,54]; Matamoros and Salvadó [51]; Ávila et al. [22,48,57]; Carranza-Diaz et al. [41]	
	Photodegradation	Ávila et al. [46,48]	
**Flame retardants**			
Tributyl phosphate	Biodegradation	Matamoros et al. [28]	Sorption **; Biodegradation (aerobic) **
	Sorption	NA	
Triphenyl phosphate	Biodegradation	Matamoros et al. [28]	Biodegradation (aerobic) **; Sorption **
	Sorption	NA	
Tris (2-chloroethyl) phosphate	Recalcitrant to biodegradation	Matamoros and Salvadó [51]; Matamoros et al. [28,29]	Sorption **
	Sorption	NA	
	Plant uptake	NA	
**Sunscreen agents**			
Oxybenzone	Biodegradation (aerobic)	Matamoros and Salvadó [51]; Ávila et al. [22,46,57]	Adsorption **; Biodegradation (aerobic); Sorption
	Sorption	Matamoros and Salvadó [51]	
	Adsorption	NA	
Sulisobenzone	NA	NA	Biodegradation (aerobic) **

Note: Authors’ own insight based on physicochemical properties, removal mechanisms, and limited evidence in the literature (*); Authors’ own insight based on physicochemical properties and removal mechanisms (**).

In this section, synthesis on six selected PCPs is presented, for which sufficient data (three or more data points) are available in two or more types of CWs for statistical comparison to choose the best possible design of CWs. Detailed discussion about the other nine PCPs is given in Table S9 (Supplementary Materials). The removal efficiency of 15 studied PCPs with four types of CWs (FWSCW, HFCW, VFCW, and HCW) is presented in Table S10 (Supplementary Materials). The results of one-way ANOVA and z-Test for comparison of means for six selected PCPs with different types of CWs are presented in Table S11 (Supplementary Materials).

#### 3.3.1. Preservatives

**Methylparaben**: The removal efficiency of methylparaben was higher in FWSCW (90 ± 1%), moderate in HCW (55 ± 22%), and comparatively lower in HFCW (23 ± 21%) (Table S10, Supplementary Materials). Methylparaben is highly water soluble (5.98 g L^−1^ at 25 °C) and has low hydrophobicity and distribution coefficient (Log Kow = 2.00; Log Dow = 1.63) with low molecular weight (152.15 g mol^−1^) and low organic carbon sorption capacity (Log Koc = 2.11) (Table 4), which suggests that adsorption onto soil particles and sorption onto organic surfaces cannot be considered as one of its major removal mechanisms. Moreover, it is a neutral compound under neutral conditions (pH = 7) (Table 4) and neutral compounds which are hydrophilic in nature might be taken up by rooted vascular plants via hydrogen bonding with water molecules into the transpiration stream [40]. Petrie et al. [40] confirmed its removal by plant uptake and calculated its high concentration (197 μg kg^−1^) in the plants. Similarly, Anjos et al. [39] attributed its removal to plant uptake. The removal efficiency by FWSCW, planted with *Landoltia punctata* and *Lemna minor* was 91% and 89%, respectively (Table S1, Supplementary Materials). Additionally, the indirect positive effects of plants’ presence such as degradation by enzymatic exudates as well as release of oxygen and root exudates (such as carbohydrates and amino acids) by the plant roots in the rhizosphere, which can provide organic carbon and a nutrient source for microorganisms helping them to degrade aerobically [30,36,55,58], might contribute to its removal [36]. Furthermore, Chen et al. [27] ascribed its removal to photolysis, hydrolysis, and volatilization. The role of these removal pathways is evident in its higher removal efficiency in summer compared with winter (56% and 33%, respectively) [28] (Table S4, Supplementary Materials) due to the higher activity of the rooted plants in the warm season, as well as the enhancement in biodegradation and photodegradation processes in summer [28,29].

#### 3.3.2. Antiseptics

**Triclosan**: The removal efficiency of triclosan was higher in FWSCW (97 ± 2%) and VFCW (88 ± 9%), but moderate in HCW (77 ± 19%) and HFCW (59 ± 31%) (Table S10, Supplementary Materials). Triclosan’s very low water solubility (10 mg L^−1^ at 25 °C), high hydrophobicity (Log Kow = 5.34; Log Dow = 4.76) with moderate molecular weight (289.55 g mol^−1^), and neutral or anionic nature under neutral conditions (pH = 7) with pKa value of 7.9 (Table 4), suggest its removal by adsorption onto soil particles following complex formation with metal ions such as Ca^2+^, Mg^2+^, Fe^3+^ or Al^3+^ [59]. This can be explained by its better removal efficiency in winter (45%) compared with summer (35%) [28] (Table S4, Supplementary Materials), because abiotic processes like adsorption are exothermic processes and favored by low temperature (in winter) [16]. Its high organic carbon sorption capacity (Log Koc = 4.26) also favors its removal by sorption, which can be seen by its sorption (19%) to the vessel of hydroponic microcosm [36] (Figure 3; Table S5, Supplementary Materials). The dominance of adsorption/sorption processes in its removal is further supported by the almost similar removal efficiency in the planted and unplanted CWs (54 ± 65% and 51 ± 69%, respectively) [41,44] (Tables S2 and S3, Supplementary Materials) as well as lower contribution of plants (11%) in the hydroponic system (*Spirodela polyrhiza*) compared with the control without plants (95% and 84%, respectively) [21]. Its translocation factor was zero or below 1.0 from roots to the shoots of the plant, which indicates rhizofiltration as one of the sources of remediation [45]. Similarly, Petrie et al. [40] did not observe its uptake by any of the studied plants. However, the presence of plants enhances microbial activity (biodegradation), which might be responsible for its removal [21,22,26,49]. This can be seen by the high contribution (up to 84%) of this process in its removal efficiency in the case of hydroponic microcosms [21] (Figure 3; Table S5, Supplementary Materials). Next to adsorption and sorption, triclosan’s higher removal efficiency in FWSCW suggests that photodegradation might be a considerable removal pathway [11,28,31,48,51], since its high removal efficiency by photodegradation was achieved in hydroponic microcosm (69 ± 16%) [21,36] (Figure 3; Table S5, Supplementary Materials).

#### 3.3.3. Fragrances

**Methyl dihydrojasmonate**: The removal efficiency of methyl dihydrojasmonate was moderate in HCW (76 ± 19%), HFCW (73 ± 21%), and FWSCW (71 ± 20%) (Table S10, Supplementary Materials). Its moderate hydrophobicity (Log Kow = 2.98), slight water solubility (91.7 mg L^−1^ at 25 °C) with moderate molecular weight (226.31 g mol^−1^), and neutral form under neutral conditions (pH = 7) (Table 4) indicate that uptake by plants is one of its main removal mechanisms in CWs. The contribution of this removal pathway is explicitly shown by the higher removal efficiency in hydroponic system (72 ± 22%) [14,15,16] (Figure 3; Table S5, Supplementary Materials). This can also be explained by the better removal efficiency in the planted compared with the unplanted HFCW (76 ± 19% and 66 ± 25%, respectively) [14,15,16,55] (Table S2, Supplementary Materials), and planted and unplanted HCW (78 ± 20% and 72 ± 19%, respectively) [14,15,16,28,51,53,54] (Table S4, Supplementary Materials). Although its sorption capacity is moderate (Log Koc = 2.18), due to its neutral nature under neutral pH conditions its binding to biomass through cation exchange with anionic sites is likely to be minimal. However, it was physically retained on the root surface [15]. On the other hand, the indirect positive effects of plants’ presence such as biodegradation might contribute to its removal. This can be elucidated by its higher removal efficiency in summer compared with winter (88 ± 7% and 52 ± 19%, respectively) [14,16,28,54] due to the higher activity of the rooted plants in the warm season as well as the enhancement of biodegradation in summer [28,54].

**Galaxolide**: The removal efficiency of galaxolide was moderate in HCW (65 ± 22%), FWSCW (63 ± 26%), and HFCW (53 ± 26%) (Table S10, Supplementary Materials). It is highly hydrophobic (Log Kow = 6.26), and almost insoluble in water (1.75 mg L^−1^ at 25 °C) with moderate molecular weight (258.4 g mol^−1^) (Table 4), which indicate that the removal by adsorption onto soil particles can be considered its main removal mechanism. Due to this reason, in the HFCW and HCW which represented full substrate system and half substrate system, respectively, its higher removal efficiency is expected in HFCW. However, HCW showed twice the removal efficiency of HFCW (60 ± 13% and 30 ± 12%, respectively) [14,15,16] (Tables S2 and S4, Supplementary Materials). This could be due to the presence of microscopic algae and other suspended solids as well as macrophyte leaves, stems, and roots in the free-water layer of the HCW, which could act as important adsorption surfaces [14]. Its hydrophobic nature favors its adsorption on the root exodermis, since it is in neutral form under neutral conditions (pH = 7) (Table 4), which would facilitate its uptake by plants [15]. The contribution of this removal pathway is exemplified by its removal efficiency in the hydroponic system (61 ± 29%) [14,15,16] (Figure 3; Table S5, Supplementary Materials). This can also be seen by the higher removal efficiency in planted compared with unplanted HFCW (62 ± 26% and 32 ± 14%, respectively) [14,15,16,41,55] (Table S2, Supplementary Materials), and planted and unplanted HCW (69 ± 18% and 51 ± 25%, respectively) [14,15,16,54] (Table S4, Supplementary Materials). Its high organic carbon sorption capacity (Log Koc = 4.10) (Table 4) also favors its removal by sorption onto organic solid surfaces [14,19,28,41,54,56]. The roots of the plants in the CWs increase the accumulation of organic matter as well as the sorption capacity [51]. This can be demonstrated by its higher removal efficiency in summer compared with winter (65 ± 24% and 42 ± 26%, respectively) [14,16,28,54,60] due to the efficient growth of the rooted plants in the warm season.

**Tonalide**: The removal efficiency of tonalide was moderate in VFCW (74 ± 11%), HCW (72 ± 20%), and FWSCW (59 ± 27%), and comparatively lower in HFCW (43 ± 25%) (Table S10, Supplementary Materials). It is highly hydrophobic (Log Kow = 6.35; Log Dow = 5.80), almost insoluble in water (1.25 mg L^−1^ at 25 °C) with moderate molecular weight (258.4 g mol^−1^) (Table 4), which favors its removal by adsorption onto soil particles. Therefore, in the HFCW and HCW which represented full substrate system and half substrate system, respectively, its higher removal efficiency is expected in HFCW. However, HCW showed more than twice the removal efficiency of HFCW (63 ± 8% and 30 ± 12%, respectively) [14,15,16] (Tables S2 and S4, Supplementary Materials). This might be due to its adsorption on the available microscopic algae, other suspended solids, macrophyte leaves, stems, and roots in the free-water layer of the HCW [14]. Its hydrophobic nature supports its adsorption on the root exodermis, and neutral form under neutral conditions (pH = 7) (Table 4) would assist its uptake by plants [15]. The contribution of this removal pathway is explicitly shown by its removal efficiency in the hydroponic system (55 ± 29%) [14,15,16] (Figure 3; Table S5, Supplementary Materials), which can also be explained by its better removal efficiency in planted compared with unplanted HFCW (48 ± 26% and 25 ± 14%, respectively) [14,15,16,41] (Table S2, Supplementary Materials), and planted and unplanted HCW (65 ± 12% and 54 ± 24%, respectively) [14,15,16,54] (Table S4, Supplementary Materials). Its high Log Koc = 4.27 (Table 4) favors its removal by sorption onto organic surfaces [14,19,22,28,41,54,56,57], which is evident in its higher removal efficiency in summer compared with winter (62 ± 21% and 45 ± 26%, respectively) [14,16,28,54,60] due to the higher activity of the rooted plants in the warm season. The roots of the plants in the CWs increase the accumulation of organic matter as well as the sorption capacity [51]. Furthermore, some studies have suggested that photodegradation might contribute to its removal efficiency in FWSCW [46,48].

#### 3.3.4. Sunscreen Agents

**Oxybenzone**: The removal efficiency of oxybenzone was higher in VFCW (94 ± 4%) and HCW (88 ± 8%), and moderate in HFCW (64 ± 28%) (Table S10, Supplementary Materials). Its moderate hydrophobicity (Log Kow = 3.52; Log Dow = 3.06) with low water solubility (68.6 mg L^−1^ at 25 °C), and moderate molecular weight (228.25 g mol^−1^) (Table 4) indicate that the removal by adsorption onto soil particles can be considered one of its main removal mechanisms. This can be indicated by its higher removal efficiency in winter compared with summer (87% and 78%, respectively) [28] (Table S4, Supplementary Materials), which is favored by low temperature (in winter). It is neutral in nature under neutral conditions (pH = 7), which favors its uptake by plants, but its low water solubility hinders its removal by this pathway. This might be the reason that it was not detected in any of the studied plants [40]. However, the presence of rooted plants in CW increases its capacity to accumulate organic matter, to develop biofilm and to transport oxygen. This also increases its capacity to remove biodegradable organic pollutants [51], which might be one of its important removal pathways owing to its moderate organic carbon sorption capacity (Log Koc = 2.63) (Table 4). 

**Table 4:** Physicochemical properties of 15 selected PCPs.

Class/PCPs/ Molecular Weight (g mol^−1^)	Molecular Formula	Molecular Structure	Water Solubility at 25 °C (mg L^−1^)	Log Kow	Log Koc	Log Dow	Henry’s Law Constant (atm m^3^ mol^−1^)	pKa/ Charge at pH 7	Reference
**Artificial sweeteners**									
Acesulfame/ 163.15	C_4_H_5_NO_4_S	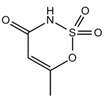	2.7 × 10^5^	−1.33	0.347	−1.49	9.63 × 10^−9^	2.0/ negative	(1); (2); (3); Zou et al. [61]; Magnuson et al. [62]
**Preservatives**									
Methylparaben/ 152.15	C_8_H_8_O_3_	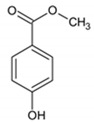	5.98 × 10^3^	2.00	2.11	1.63	3.61 × 10^−9^	8.3/ neutral	(2); (3); Petrie et al. [40]
Propylparaben/ 180.21	C_10_H_12_O_3_	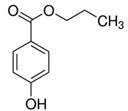	529.3	2.98	2.71	2.51	6.37 × 10^−9^	8.2/ neutral	(2); (3); Petrie et al. [40]
**Insect repellents**									
N,N-diethyl-meta-toluamide/ 191.3	C_12_H_17_NO	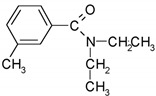	912	2.18	1.76	2.50	2.10 × 10^−8^	0.7/ neutral	(2); Conkle et al. [63]; Anumol et al. [64]; Li et al. [21]; Yi et al. [5]; Sgroi et al. [20]
**Antiseptics**									
Triclosan/ 289.55	C_12_H_7_Cl_3_O_2_	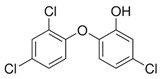	10	5.34	4.26	4.76	2.13 × 10^−8^	7.9/ neutral; negative	(2); Park et al. [50]; Verlicchi et al. [12,65]; Zhang et al. [11]; Zhu and Chen [25]; Carranza-Diaz et al. [41]; Dai et al. [30]; Li et al. [21]; Vystavna et al. [47]; Petrie et al. [40]; Wang et al. [45]
Triclocarban/ 315.6	C_13_H_9_Cl_3_N_2_O	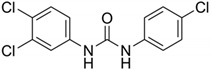	0.11	4.90	3.73	4.90	4.50 × 10^−11^	12.8/ neutral	(2); Zhu and Chen [25]; Anumol et al. [64]; Verlicchi et al. [12]; Chen et al. [26]
**Fragrances**									
Methyl dihydrojasmonate/ 226.31	C_13_H_22_O_3_	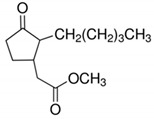	91.7	2.98	2.18	NA	5.02 × 10^−7^	−6.9/ neutral	(2); (4) Hijosa-Valsero et al. [14,15,54]; Reyes-Contreras et al. [16]; Zhang et al. [11]; Verlicchi et al. [12]
Cashmeran/ 206.33	C_14_H_22_O	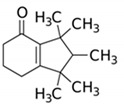	5.94	4.49	3.60	NA	1.42 × 10^−4^	−5.1/ neutral	(2); (3); (5); Hijosa-Valsero et al. [14]; Verlicchi et al. [12]
Galaxolide/ 258.4	C_18_H_26_O	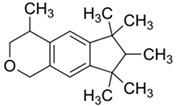	1.75	6.26	4.10	NA	1.32 × 10^−4^	8.24/ neutral	(2); (3); Hijosa-Valsero et al. [14,15,54]; Reyes-Contreras et al. [16]; Zhang et al. [11]; Verlicchi et al. [12]
Tonalide/ 258.41	C_18_H_26_O	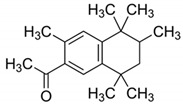	1.25	6.35	4.27	5.80	4.22 × 10^−5^	16/ neutral	(2); (3); Ávila et al. [66]; Hijosa-Valsero et al. [14,15,54]; Reyes-Contreras et al. [16]; Zhang et al. [11]; Verlicchi et al. [12]
**Flame retardants**									
Tributyl phosphate/ 266.32	C_12_H_27_O_4_P	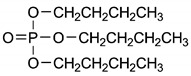	280	4.00	3.24	NA	3.19 × 10^−6^	19/ neutral	(2); (3); Bergman et al. [67]
Triphenyl phosphate/ 326.29	C_18_H_15_O_4_P	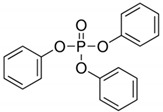	1.9	4.70	3.24	NA	3.98 × 10^−8^	16.4/ neutral	(2); (3); Brooke et al. [68]
Tris (2-chloroethyl) phosphate/ 285.48	C_6_H_12_Cl_3_O_4_P	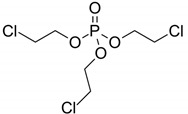	7.82 × 10^3^	1.63	2.48	NA	2.55 × 10^−8^	16.1/ neutral	(3); (6); (7); Xu et al. [69]
**Sunscreen agents**									
Oxybenzone/ 228.25	C_14_H_12_O_3_	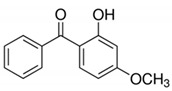	68.6	3.52	2.63	3.06	1.50 × 10^−8^	7.92/ neutral	(2); (3); (7); Zhang et al. [11]; Verlicchi et al. [12]; Petrie et al. [40]
Sulisobenzone/ 308.31	C_14_H_12_O_6_S	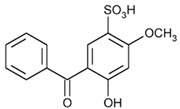	2.03 × 10^4^	0.37	1.55	−0.53	7.06 × 10^−15^	1.99/ negative	(2); (3); (8); Petrie et al. [40]

Note: https://www.drugfuture.com/chemdata/ (1); https://www.ncbi.nlm.nih.gov/pccompound (2); QSAR Toolbox 4.3.1 (3); http://www.hmdb.ca/metabolites/HMDB0031740 (4); http://contaminantdb.ca/contaminants/CHEM008153 (5); https://www.sigmaaldrich.com/nederland.html (6); http://www.t3db.ca/toxins/T3D4950 (7); https://www.drugbank.ca/drugs/DB11185 (8); Molecular structures are taken from website: https://images.google.com/.

### 3.4. Statistical Comparison of Different Types of CWs for PCPs Removal

As discussed above, the removal efficiency of PCPs in CWs is affected by their physicochemical properties, which leads to an extremely high compound specific variation (Figure 1; Table S6, Supplementary Materials). Similarly, Ilyas and van Hullebusch [70] comprehensively discovered that the removal efficiency of PCPs is governed by several design and operational factors (area, depth, HLR, OLR, and HRT), and physicochemical parameters (DO, temperature, and pH) of CWs, albeit with different degrees of influence on individual PCPs. In addition, the removal efficiency of widely studied PCPs was analyzed when CWs were used for primary, secondary, and tertiary treatment (Table S12, Supplementary Materials). However, the results indicate no clear pattern of high or low performance in case of primary, secondary or tertiary treatment. For instance, in some cases higher removal efficiencies are achieved when CWs are used as tertiary treatment compared to primary treatment and vice versa. These results are consistent with Verlicchi and Zambello [10], and Verlicchi et al. [12], as they also indicated a very high variability in removal efficiency of PCPs under the studied CWs used as primary, secondary, and tertiary treatment. Therefore, it is challenging to establish the level of treatment for improved performance and risk attenuation by CWs.

**Figure 4:**
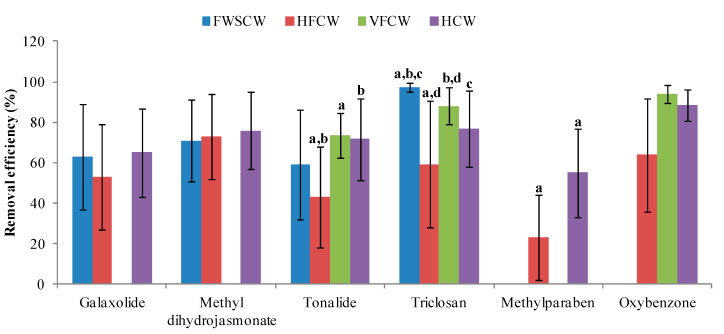
Removal efficiency of six selected PCPs with different types of CWs. Note: Tonalide: ‘a’ shows that horizontal flow constructed wetlands (HFCW) exhibit significant difference from vertical flow constructed wetlands (VFCW); ‘b’ shows that HFCW and hybrid constructed wetlands (HCW) are significantly different from each other; Triclosan: ‘a’ shows that free water surface constructed wetlands (FWSCW) exhibit significant difference from HFCW; ‘b’ shows that FWSCW and VFCW are significantly different from each other; ‘c’ shows that FWSCW exhibit significant difference from HCW; ‘d’ shows that HFCW and VFCW are significantly different from each other; Methylparaben: ‘a’ shows that HFCW exhibit significant difference from HCW at α = 0.05 (*p* < 0.05); The number of observations for studied PCPs in different types of CWs is given in Table S10 (Supplementary Materials).

It can be seen from the results that the recent studies have examined a higher number of PCPs and several new studies have comprehensively investigated the occurrence and removal of these compounds. Thus, we could compile data on 20 PCPs from different sources. Although it is a promising trend, the number of data points for several compounds is still limited. Furthermore, contradictory results on the removal of several PCPs (Tables S1–S4, Supplementary Materials) obstruct generalization of results based on an individual case study. Therefore, a statistical comparison is not possible for every compound. However, six PCPs that were studied by several authors and in more than two types of CWs were identified for statistical comparison. For the six selected PCPs, CWs demonstrate a moderate to high potential for successful treatment with an average removal efficiency > 50% in the case of five PCPs (oxybenzone, methyl dihydrojasmonate, triclosan, tonalide, and galaxolide) with the exception of methylparaben (46%) (Figure 1). The results of ANOVA and z-Test for comparison of means, for statistical significance or non-significance of observed difference among studied CWs for the removal of six selected PCPs (Table S11, Supplementary Materials), are substantiated by Figure 4 and discussed in this section.

The removal efficiency of tonalide was moderate in VFCW (74 ± 11%), HCW (72 ± 20%), and FWSCW (59 ± 27%) but lower in HFCW (43 ± 25%) (Table S10, Supplementary Materials). The removal efficiency in HFCW is significantly lower compared with VFCW and HCW (Figure 4; Table S11, Supplementary Materials). Although the removal efficiency in FWSCW is much lower compared with VFCW and HCW, this does not reveal statistically significant differences. Similarly, the removal efficiency in FWSCW was also higher compared with HFCW, but does not show significant differences (Figure 4; Table S11, Supplementary Materials). However, its low to moderate removal efficiency in the studied CWs might be due to the major processes responsible for its removal such as sorption onto organic surfaces, adsorption, and retention on the root exodermis, as well as plant uptake (Table 3).

The removal efficiency of triclosan was significantly higher in FWSCW (97 ± 2%) compared with VFCW (88 ± 9%), HCW (77 ± 19%), and HFCW (59 ± 31%) (Figure 4; Tables S10 and S11, Supplementary Materials). Although adsorption and/or sorption is one of its main removal mechanisms, its higher removal efficiency in FWSCW suggests that photodegradation might be a considerable removal pathway (Table 3). Its uptake by plants cannot be considered in CWs, but the indirect positive effects of plants’ presence such as biodegradation contributed to its removal. Its major removal process in CWs is aerobic biodegradation. However, some studies also attributed its removal to anaerobic biodegradation (Table 3). VFCW are predominantly aerobic compared with anoxic HFCW. Although it is an easily biodegradable compound, the significantly higher removal efficiency in VFCW compared with HFCW (Figure 4; Table S11, Supplementary Materials) can be explained by the fact that the aerobic biodegradation mainly contributes to its microbial degradation process, thus, removal efficiency increases under oxic conditions. The comparatively better removal efficiency in HCW than HFCW might be due to the establishment of aerobic and anaerobic conditions. Consistent with that, the removal efficiency of methylparaben was almost twice in HCW (55 ± 22%) compared with HFCW (23 ± 21%) (Table S10, Supplementary Materials), and exhibits significant differences between them (Figure 4; Table S11, Supplementary Materials). Its low to moderate removal efficiency might be due to its major removal processes in CWs such as biodegradation and plant uptake. Additionally, its removal was also attributed to photodegradation and volatilization processes (Table 3), which are dominant in FWSCW. Based on limited evidence its higher removal efficiency was observed in FWSCW (90 ± 1%) (Table S10, Supplementary Materials).

The removal efficiency of methyl dihydrojasmonate was slightly better in HCW (76 ± 19%) compared with HFCW (73 ± 21%), and FWSCW (71 ± 20%) (Table S10, Supplementary Materials) but this does not exhibit statistical significance in differences (Figure 4; Table S11, Supplementary Materials). However, the slight improvement in its removal efficiency in HCW might be due to the co-existence of aerobic and anaerobic micro-environments in the system. Similarly, the removal efficiency of galaxolide was moderate in HCW (65 ± 22%) and FWSCW (63 ± 26%), and comparatively better than HFCW (53 ± 26%) (Table S10, Supplementary Materials) but does not reveal significant differences (Figure 4; Table S11, Supplementary Materials). The moderate removal efficiency in the studied CWs might be due to the fact that major processes responsible for its removal are sorption onto organic surfaces and adsorption as well as retention on the root exodermis, besides plant uptake. Analogous to this, the removal efficiency of oxybenzone was much higher in VFCW (94 ± 4%) and HCW (88 ± 8%) compared with HFCW (64 ± 28%) (Table S10, Supplementary Materials) but does not demonstrate significant differences (Figure 4; Table S11, Supplementary Materials). Nevertheless, the higher removal efficiency in VFCW might be due to the occurrence of an oxic condition which is beneficial for its removal. Its removal is attributed to aerobic biodegradation. The other removal pathway is sorption, which might contribute to its removal efficiency in HFCW. Furthermore, the higher removal efficiency in HCW might be due to the availability of longer HRT because of more than one compartment for wastewater treatment.

In general, there is a large variation in the reported removal efficiencies for each PCP in different types of CWs. Low or negative removal efficiency of PCPs could be due to analytical errors in the case of extremely low influent and effluent concentrations (close to detection limit) [40]. Furthermore, evapotranspiration may influence the removal processes leading to lower pollutant removal efficiency [71]. Białowiec et al. [71] studied the evapotranspiration effect on the removal efficiency estimates of COD. As a result, lower values of removal efficiency were estimated when based on influent and effluent concentration compared with mass balance determination. The authors suggest the need to monitor flow rates at the outflow and inflow, and the evaluation of potential evapotranspiration to estimate removal efficiency of pollutants based on their mass balance in CWs. Unfortunately, it is not possible to calculate the removal efficiency of PCPs in this study based on their mass balance, because sufficient information (e.g., water outflow rates and evapotranspiration rates) is not available in the studied literature.

### 3.5. Effect of Artificial Aeration (AA) on the Removal of PCPs

**Figure 5:**
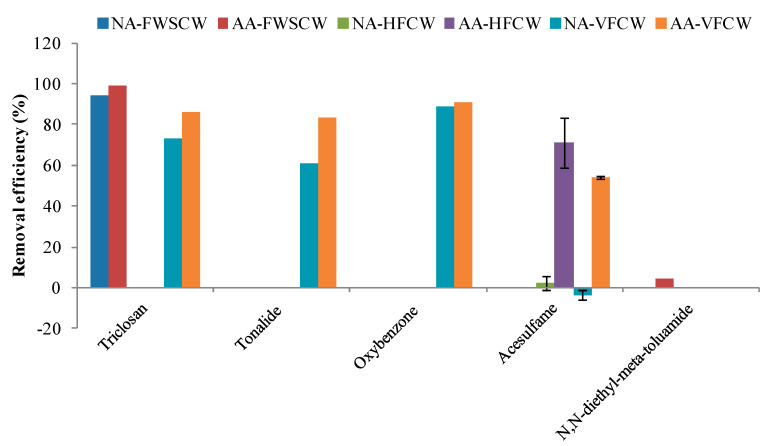
Removal efficiency of PCPs in different types of aerated (AA) and non-aerated (NA) CWs. Note: Data is taken from: Ávila et al. [22]; Li et al. [21]; Kahl et al. [17]; and Nivala et al. [18]. The enhanced removal is explicit in the case of AA-FWSCW (triclosan), AA-HFCW (acesulfame), AA-VFCW (triclosan, tonalide, oxybenzone, and acesulfame) compared with their corresponding NA-CWs.

The availability of sufficient oxygen within the system gives favorable conditions to microorganisms to complete biodegradation besides contribution in reducing clogging [72] and improving the removal efficiency of the system for organic matter, nitrogen, and phosphorus [73,74]. Oxygen also contributes to reduce the land area required by CWs [75]. It is suggested that aerobic (ORP > +100 mV) and anoxic (−100 mV < ORP < +100 mV) conditions favor the biodegradation of EOCs while facilitating biogeochemical reactions [76], which depend on the co-existing redox processes occurring at the wetland system scale and the rhizosphere scale [77]. The redox potential was considered among the main factors affecting EOCs removal [19,78,79]. The removal of PCPs in different types of CWs revealed that the configuration, operation, and ambient environmental conditions within the CW are affecting their removal efficiency. Since DO plays an important role in the removal of PCPs by CWs, to improve the level of DO some recent studies investigated the effect of AA on the performance of FWSCW, HFCW, and VFCW [17,18,21,22]. The comparative performance of different types of aerated (AA) CWs and non-aerated (NA) CWs (Figure 5; Table S13, Supplementary Materials) for the removal of studied PCPs is discussed in this section.

The improvement in the removal efficiency of aerobically biodegradable compounds (Table 3) is attributed to the elevated DO level in the AA-CWs (Table S13, Supplementary Materials). For instance, the removal efficiency of triclosan was improved in AA-FWSCW (99%) compared with NA-FWSCW (94%) [21], and in AA-VFCW (86%) compared with NA-VFCW (73%) [22] (Figure 5). However, the higher removal efficiency in AA-FWSCW compared with AA-VFCW might be due to the fact that photodegradation is one of its possible removal pathways, which is feasible in FWSCW (Table 3). Similarly, the removal efficiency of oxybenzone was slightly improved in AA-VFCW compared with NA-VFCW (91% and 89%, respectively) [22] (Figure 5).

Analogous to this, the enhancement in the removal efficiency of moderately biodegradable compounds has also been demonstrated due to improved DO level in CWs. For example, the removal efficiency of acesulfame in AA-HFCW was significantly enhanced (71 ± 12%) compared with NA-HFCW (2.5 ± 3.5%). Although it was negatively removed in NA-VFCW (−3.5 ± 2.1%), its moderate removal efficiency was achieved in AA-VFCW (54 ± 1%) [17,18] (Figure 5). Similarly, although tonalide is a typically hydrophobic compound, which is removed by sorption onto particulate matter and uptake by the plants (Table 3), the considerable improvement in its removal efficiency in AA-VFCW compared with NA-VFCW (83% and 61%, respectively) (Figure 5) is attributed to the marked increase in DO level in the AA-VFCW [22] (Table S13, Supplementary Materials). Its hydrophobic nature supports its adsorption on the root exodermis, and its neutral form under neutral conditions (pH = 7) would assist its uptake by plants [15]. However, Reyes-Contreras et al. [16] suggested that the clogging of the system due to aging effect might obstruct the efficient contribution of plants to the removal of PCPs. The availability of sufficient oxygen due to the improvement in DO with AA not only reduces the clogging of the system [72], but also promotes the development of increased biomass growth [22]. The roots of the plants in the CWs increase the accumulation of organic matter as well as the sorption capacity [51].

On the contrary, the application of AA in CWs could not contribute to the enhancement of the removal efficiency of PCPs that are better removed under anaerobic conditions. For instance**,** the removal efficiency of N,N-diethyl-meta-toluamide in NA-FWSCW and AA-FWSCW was very poor (0.0% and 4.2%, respectively) [21] (Figure 5). It has been demonstrated that this compound is not light sensitive (1.2% removal at highest by photodegradation). The lower contribution of plants (9.1%) in hydroponic system (*Spirodela polyrhiza*) compared with the control without plants (17% and 7.9%, respectively) also reveals that this removal pathway is contributing much less to its removal efficiency. Furthermore, in the *E. coli* biodegradation experiment, the highest removal efficiency observed was very low (4.5%) [21]. On the other hand, some studies observed its removal efficiency in NA-HFCW, NA-VFCW, and NA-HCW up to 98%, 28%, and 80%, respectively [5,20] (Tables S2–S4, Supplementary Materials). The highest removal efficiency in NA-HFCW followed by NA-HCW and lowest removal efficiency in NA-VFCW indicates that predominantly anaerobic and slightly aerobic conditions might favor its removal.

## 4. Future Research Needs

This study generated several new insights about the performance of CWs for PCPs removal, which could be instructive for improved understanding and guiding future research. Based on this comprehensive study, the following research needs are identified.

1. More research should be undertaken to investigate the occurrence of PCPs in water resources and the environment, especially for those PCPs which are classified under high and medium risk categories (i.e., triclosan, methylparaben, galaxolide, oxybenzone, and methyl dihydrojasmonate). Furthermore, more emphasis should be given to examine the impact of these PCPs on human and ecosystem health.
2. The HCW showed better performance compared with individual systems (e.g., VFCW, HFCW, and FWSCW), which shows the high potential for this type of CW for practical applications for the treatment of wastewater containing PCPs. Nevertheless, different types of HCWs are investigated such as FWSCW + FWSCW, HFCW + HFCW, VFCW + VFCW, VFCW + HFCW, HCW including FWSCW, and also multistage of more than two types of CWs. Therefore, further research is needed to develop the best possible integrated design of CWs to ensure the occurrence of various removal processes, which are necessary to remove multiple types of PCPs.
3. The water flow rate at both inflow and outflow should be measured to quantify the effect of evapotranspiration on the removal efficiency estimates of PCPs in different types of CWs.
4. The establishment of various micro-environments (aerobic and anaerobic conditions) in CWs by the application of AA, provides both aerobic and anaerobic metabolic pathways contributing to the removal of PCPs. However, to date, the application of AA has been considered by a very limited number of studies, which highlights the need for further research. In addition, more research is needed to investigate which type of aeration (e.g., intermittent or continuous) would be beneficial to generate such conditions to improve the performance of CWs.

## 5. Conclusions

The removal of PCPs has been investigated by different types of CWs. In this paper, a statistical comparison was made among four types of CWs (FWSCW, HFCW, VFCW, and HCW) of their removal efficiency. The environmental risk posed by a number of PCPs and the attenuation in risk after the treatment with CWs were estimated. Additionally, the impact of physicochemical properties of PCPs on their removal mechanisms was comprehensively analyzed. This comprehensive analysis reveals some specific conclusions, which are outlined below.

1. The CWs contributed considerably in reducing the environmental risks posed by PCPs. Although the risk is not fully abolished by CWs, it is significantly reduced in most cases. Our analysis of global data classified triclosan under high risk category, whereas, triclocarban, methylparaben, galaxolide, oxybenzone, and methyl dihydrojasmonate were grouped under medium risk category. These high to medium risk PCPs are recommended to be considered for regulatory monitoring, control and water quality standard formulation purposes.
2. CWs could effectively remove a large number of PCPs from wastewater, and all of the 15 widely studied compounds show a positive removal efficiency ranging from 9.0% to 84%. In most of the examined PCPs, adsorption and/or sorption is the most dominant removal mechanism (8 out of 15) followed by biodegradation (aerobic and anaerobic) (5 out of 15) and plant uptake (planted CWs) (3 out of 15), and the physicochemical properties of PCPs play a pivotal role in their removal processes.
3. The six selected PCPs, which were studied by more than two types of CWs, demonstrate a moderate to high potential for successful treatment. Among the studied CWs, the HCW performed better for most of the examined PCPs followed by VFCW, HFCW, and FWSCW. The superior performance of HCW could be mainly attributed to the co-existence of aerobic and anaerobic conditions, and longer HRT that is beneficial for the removal of PCPs (e.g., triclosan, methyl dihydrojasmonate, galaxolide, tonalide, and oxybenzone), which are removed under both conditions and by adsorption and/or sorption processes. Aerobic biodegradation being more efficient than anaerobic explains the better removal efficiency in VFCW compared with HFCW. In FWSCW, photodegradation is the main removal pathway and only few PCPs (e.g., methylparaben, propylparaben, triclosan, and tonalide) demonstrated considerable removal by this process.
4. The improvement in DO due to redox manipulation with AA enhances the removal efficiency of PCPs, which are better removed under aerobic conditions. Although anoxic bio-transformations are slower than the oxic ones, the high performance of AA-CWs could be due to the occurrence of various micro-environments (aerobic and anaerobic) and subsequent contribution of both aerobic and anaerobic metabolic pathways in the removal of PCPs. This is evident by the enhanced removal efficiency in the case of AA-FWSCW (triclosan), AA-HFCW (acesulfame), and AA-VFCW (triclosan, tonalide, oxybenzone, and acesulfame) compared with their corresponding NA-CWs.

## Figures and Tables

**Table 1:** Studied personal care products (PCPs) categorized according to their uses.

No. of Categories	Category	Personal Care Products
1	Artificial sweeteners	Sucralose, Acesulfame
2	Preservatives	Methylparaben, Propylparaben
3	Insect repellents	N,N-diethyl-3-methyl benzoylamide, N,N-diethyl-3-methyl benzamide, N,N-diethyl-meta-toluamide
4	Antiseptics	Triclosan, Triclocarban
5	Fragrances	Cashmeran, Celestolide, Galaxolide, Methyl dihydrojasmonate, Tonalide
6	Flame retardants	Tributyl phosphate, Triphenyl phosphate, Tris (2-chloroethyl) phosphate
7	Sunscreen agents	Hydrocinnamic acid, Oxybenzone, Sulisobenzone

**Table 2:** Risk assessment of 11 selected PCPs based on influent and effluent concentration in CWs.

Class/PCPs	PNEC (μg L^−1^)	(MEC) Influent Conc. (μg L^−1^)	(MEC) Effluent Conc. (μg L^−1^)	Influent RQ	Effluent RQ	Risk Rank * Influent/ Effluent	References for PNEC Values
**Preservatives**							
Methylparaben	11.2	39	4.2	3.5	0.4	High/ Medium	Yamamoto et al. [32]
**Antiseptics**							
Triclosan	0.13	39	1.3	300	10	High/ High	Kosma et al. [24]; Zhu and Chen [25]
Triclocarban	0.01	0.1	0.01	10	1.0	High/ Medium	Zhu and Chen [25]
**Fragrances**							
Methyl dihydro-jasmonate	15.8	7.1	1.9	0.4	0.1	Medium/ Medium	Matamoros et al. [28]
Cashmeran	11.6	0.2	0.03	0.02	0.003	Low/No	Brausch and Rand [33]
Galaxolide	3.5	2.2	0.9	0.6	0.3	Medium/ Medium	Balk and Ford [34]
Tonalide	6.8	0.6	0.2	0.09	0.03	Low/Low	Balk and Ford [34]
**Flame retardants**							
Tributyl phosphate	5.8	0.4	0.2	0.07	0.03	Low/Low	Cristale et al. [35]
Triphenyl phosphate	1.0	0.1	0.02	0.10	0.02	Medium/ Low	Cristale et al. [35]
Tris (2-chloroethyl) phosphate	235	0.4	0.3	0.002	0.001	No/No	Cristale et al. [35]
**Sunscreen agents**							
Oxybenzone	1.9	3.6	0.3	1.9	0.2	High/ Medium	Brausch and Rand [33]

Note: Predicted no effect concentration (PNEC); Measured environmental concentration (MEC); Risk quotient (RQ); PNEC values are taken from the referred studies; Bold values indicate a high-risk category; Risk rank is based on our results (*); Risk is categorized into four levels: high risk (RQ > 1.0), medium risk (0.1 ≤ RQ ≤ 1.0), low risk (0.01 ≤ RQ ≤ 0.1), and no risk (RQ < 0.01).

## Supplementary Materials

The following are available online at https://www.mdpi.com/1660-4601/17/9/3091/s1: Table S1. The performance of FWSCW for personal care products removal; Table S2. The performance of HFCW for personal care products removal; Table S3. The performance of VFCW for personal care products removal; Table S4. The performance of HCW for personal care products removal; Table S5. Contribution of removal mechanisms/pathways of PCPs in hydroponic microcosms, media adsorption experiments, and CWs; Table S6. Mean and standard deviation of 15 widely studied personal care products; Table S7. The statistics on risk quotient of nine selected PCPs based on effluent concentration in CWs; Table S8. Role of physicochemical properties of EOCs in their removal mechanisms; Table S9. Role of physicochemical properties of PCPs and removal mechanisms in CWs; Table S10. Removal efficiency (mean % and standard deviation) of 15 widely studied PCPs in different types of CWs; Table S11. The results (*p*-values) of one-way ANOVA and z-Test for comparison of means for six selected PCPs with different types of CWs; Table S12. Statistics (mean and standard deviation) of widely studied 15 PCPs in primary, secondary, and tertiary treatment by CWs; Table S13. Removal efficiency (mean % and standard deviation) of PCPs in different types of aerated (AA) and non-aerated (NA) CWs.
