# Supplementary material for: Performance Comparison of Different Constructed Wetlands Designs for the Removal of Personal Care Products
Source: Int J Environ Res Public Health. 2020 Apr 29;17(9):3091. doi: 10.3390/ijerph17093091 (PMC7246432; doi:10.3390/ijerph17093091)
Supplement: Supplementary file 1 [file ijerph-17-03091-s001.pdf]

Supplementary Materials

Performance Comparison of Different Constructed Wetlands Designs for the Removal of Personal Care Products

Huma Ilyas [1,2,*] and Eric D. van Hullebusch [1]

[1] Université de Paris, Institut de physique du globe de Paris, CNRS, F-75005 Paris, France; vanhullebusch@ipgp.fr
[2] Water Treatment and Management Consultancy, B.V., 2289 ED Rijswijk, The Netherlands
* Correspondence: hi.wtmconsult@gmail.com

Supplementary materials 1: The treatment performance of four types of CWs (FWSCW, HFCW, VFCW, and HCW) for the removal of personal care products.

Table S1. The performance of FWSCW for personal care products removal.

Wetland type/Scale /type of treatment	WT	PCPs	Depth (m)/ Area (m^2 PE^{-1})	T (°C) /pH	HLR (m^3 m^{-2} d^{-1})/ OM	OLR (g COD m^{-2} d^{-1})	HRT (days) /SA/ED (months)	Filter media (mm)/Plants	Effluent DO (mg L^{-1})/ ORP (Inf/Eff) (mV)	Influent/ Effluent conc. (μg L^{-1})	Removal rate (mg m^{-2} d^{-1})/ Removal (%)	Author/ Country
Full/ Tertiary	*	Galaxolide	3.5/0.1	17/8.1	0.01/NA	0.2	30/36/2	NA/*Phragmites australis, Typha latifolia*	8.4/NA	2.9/0.4	0.02/85	Matamoros et al. [1] /Spain
	*	Tonalide	3.5/0.1	17/8.1	0.01/NA	0.2	30/36/2	As above	8.4/NA	0.9/0.1	0.01/88	
Full/ Tertiary	*	Galaxolide	3.5/0.1	7.8/7.3	0.01/NA	0.2	30/36/2	NA/*Phragmites australis, Typha latifolia*	8.9/NA	2.9/0.3	0.03/88	Matamoros et al. [1] /Spain
	*	Tonalide	3.5/0.1	7.8/7.3	0.01/NA	0.2	30/36/2	As above	8.9/NA	0.9/0.1	0.01/90	
Full/ Tertiary	*	Galaxolide	3.5/0.1	NA/NA	0.01/NA	0.7	30/36/2	NA/*Phragmites australis, Typha latifolia*	NA/NA	2.9/0.4	0.02/87	Llorens et al. [2]/Spain
	*	Tonalide	3.5/0.1	NA/NA	0.01/NA	0.7	30/36/2	As above	NA/NA	0.9/0.1	0.01/89	
Lab/ Secondary	D	Methyl dihydro-jasmonate	0.5/18	18/6.9	0.05/CF	5.0	2.1/17/3	Soilless system/*Typha angustifolia*	0.6 (0.5/0.7)/ +26(+36/ -62)	4.0/0.7	0.2/82	Hijosa-Valsero et al. [3]/Spain
	D	Galaxolide	0.5/18	18/6.9	0.05/CF	5.0	2.1/17/3	As above	As above	1.2/0.3	0.05/78	
	D	Tonalide	0.5/18	18/6.9	0.05/CF	5.0	2.1/17/3	As above	As above	0.4/0.1	0.01/75	
Lab/ Secondary	D	Methyl dihydro-jasmonate	0.5/18	17/7.1	0.05/CF	5.0	3.3/17/3	25 cm Siliceous gravel (4.0)/ *Typha angustifolia*	0.5 (0.6/0.3)/ +26/(+63/ -101)	4.0/0.8	0.2/80	Hijosa-Valsero et al. [3]/Spain
	D	Galaxolide	0.5/18	17/7.1	0.05/CF	5.0	3.3/17/3	As above	As above	1.2/0.5	0.03/58	
	D	Tonalide	0.5/18	17/7.1	0.05/CF	5.0	3.3/17/3	As above	As above	0.4/0.2	0.01/50	

Wetland type/Scale /type of treatment	WT	PCPs	Depth (m)/ Area (m^2 PE^{-1})	T (°C) /pH	HLR (m^3 m^{-2} d^{-1})/ OM	OLR (g COD m^{-2} d^{-1})	HRT (days) /SA/ED (months)	Filter media (mm)/Plants	Effluent DO (mg L^{-1})/ ORP (Inf/Eff) (mV)	Influent/ Effluent conc. (µg L^{-1})	Removal rate (mg m^{-2} d^{-1})/ Removal (%)	Author/ Country
Lab/ Secondary	D	Methyl dihydro-jasmonate	0.5/18	19/6.6	0.05/CF	5.0	2.9/17/3	Soilless system/ *Phragmites australis*	0.7 (0.8/0.5)/ +26/(+48/ -25)	4.0/0.2	0.2/96	Hijosa-Valsero et al. [3]/Spain
	D	Galaxolide	0.5/18	19/6.6	0.05/CF	5.0	2.9/17/3	As above	As above	1.2/0.3	0.05/78	
	D	Tonalide	0.5/18	19/6.6	0.05/CF	5.0	2.9/17/3	As above	As above	0.4/0.1	0.01/75	
Lab/ Secondary	D	Methyl dihydro-jasmonate	0.5/6.0	6.9/6.7	0.05/CF	25	2.1/11/3	Soilless system/*Typha angustifolia*	0.6 (0.7/0.4)/ -6/(+22/ -25)	12/6.0	0.3/50	Hijosa-Valsero et al. [3]/Spain
	D	Galaxolide	0.5/6.0	6.9/6.7	0.05/CF	25	2.1/11/3	As above	As above	0.9/0.5	0.02/40	
	D	Tonalide	0.5/6.0	6.9/6.7	0.05/CF	25	2.1/11/3	As above	As above	0.4/0.3	0.005/25	
Lab/ Secondary	D	Methyl dihydro-jasmonate	0.5/6.0	5.6/6.8	0.05/CF	25	3.3/11/3	25 cm Siliceous gravel (4.0)/ *Typha angustifolia*	0.6 (0.6/0.6)/ -6/(-10/ -3)	12/6.0	0.3/50	Hijosa-Valsero et al. [3]/Spain
	D	Galaxolide	0.5/6.0	5.6/6.8	0.05/CF	25	3.3/11/3	As above	As above	0.9/0.7	0.01/22	
	D	Tonalide	0.5/6.0	5.6/6.8	0.05/CF	25	3.3/11/3	As above	As above	0.4/0.3	0.004/20	
Lab/ Secondary	D	Methyl dihydro-jasmonate	0.5/6.0	6.4/7.0	0.05/CF	25	2.9/11/3	Soilless system/ *Phragmites australis*	0.6 (0.6/0.5)/ -6/(-10/ -50)	12/7.2	0.2/40	Hijosa-Valsero et al. [3]/Spain
	D	Galaxolide	0.5/6.0	6.4/7.0	0.05/CF	25	2.9/11/3	As above	As above	0.9/0.8	0.003/7.0	
	D	Tonalide	0.5/6.0	6.4/7.0	0.05/CF	25	2.9/11/3	As above	As above	0.4/0.38	0.001/4.0	
Lab/ Secondary	D	Methyl dihydro-jasmonate	0.5/18	18/6.9	0.05/CF	6.0	0.5/36/9	Soilless system/*Typha angustifolia*	0.9 (1.0/0.8)/ +2.7/(+65 /+14)	5.8/0.7	0.3/88	Reyes-Contreras et al. [4]/Spain
	D	Galaxolide	0.5/18	18/6.9	0.05/CF	6.0	0.5/36/9	As above	As above	2.1/0.3	0.09/87	
	D	Tonalide	0.5/18	18/6.9	0.05/CF	6.0	0.5/36/9	As above	As above	0.4/0.1	0.02/76	

Wetland type/Scale /type of treatment	WT	PCPs	Depth (m)/ Area (m^2 PE^{-1})	T (°C) /pH	HLR (m^3 m^{-2} d^{-1})/ OM	OLR (g COD m^{-2} d^{-1})	HRT (days) /SA/ED (months)	Filter media (mm)/Plants	Effluent DO (mg L^{-1})/ ORP (Inf/Eff) (mV)	Influent/ Effluent conc. (µg L^{-1})	Removal rate (mg m^{-2} d^{-1})/ Removal (%)	Author/ Country
Lab/ Secondary	D	Methyl dihydro-jasmonate	0.5/8.4	6.9/6.7	0.05/CF	17	0.5/30/6	Soilless system/*Typha angustifolia*	0.4/-41/ (-47/-25)	6.6/3.2	0.2/51	Reyes-Contreras et al. [4]/Spain
	D	Galaxolide	0.5/8.4	6.9/6.7	0.05/CF	17	0.5/30/6	As above	As above	1.5/0.8	0.03/44	
	D	Tonalide	0.5/8.4	6.9/6.7	0.05/CF	17	0.5/30/6	As above	As above	0.3/0.2	0.01/38	
Lab/ Secondary	D	Methyl dihydro-jasmonate	0.5/18	17/7.1	0.05/CF	6.0	0.4/36/9	25 cm Siliceous gravel (4.0)/ *Typha angustifolia*	1.0 (1.5/0.6)/ +2.7/(+93 /-65)	5.8/0.8	0.3/87	Reyes-Contreras et al. [4]/Spain
	D	Galaxolide	0.5/18	17/7.1	0.05/CF	6.0	0.4/36/9	As above	As above	2.1/0.6	0.08/73	
	D	Tonalide	0.5/18	17/7.1	0.05/CF	6.0	0.4/36/9	As above	As above	0.4/0.1	0.01/68	
Lab/ Secondary	D	Methyl dihydro-jasmonate	0.5/8.4	5.6/6.8	0.05/CF	17	0.4/30/6	25 cm Siliceous gravel (4.0)/ *Typha angustifolia*	0.6 (0.6/0.6)/ -41/(-22/ -81)	6.6/3.3	0.2/50	Reyes-Contreras et al. [4]/Spain
	D	Galaxolide	0.5/8.4	5.6/6.8	0.05/CF	17	0.4/30/6	As above	As above	1.5/0.9	0.03/40	
	D	Tonalide	0.5/8.4	5.6/6.8	0.05/CF	17	0.4/30/6	As above	As above	0.3/0.2	0.01/40	
Lab/ Secondary	D	Methyl dihydro-jasmonate	0.5/18	19/6.6	0.05/CF	6.0	1.1/36/9	Soilless system/ *Phragmites australis*	0.9 (1.2/0.7)/ +2.7/(+54 /-14)	5.8/0.3	0.3/94	Reyes-Contreras et al. [4]/Spain
	D	Galaxolide	0.5/18	19/6.6	0.05/CF	6.0	1.1/36/9	As above	As above	2.1/0.4	0.08/81	
	D	Tonalide	0.5/18	19/6.6	0.05/CF	6.0	1.1/36/9	As above	As above	0.4/0.1	0.02/73	
Lab/ Secondary	D	Methyl dihydro-jasmonate	0.5/8.4	6.4/7.0	0.05/CF	17	1.1/30/6	Soilless system/ *Phragmites australis*	0.5/-41/ (-54/-50)	6.6/3.4	0.2/49	Reyes-Contreras et al. [4]/Spain
	D	Galaxolide	0.5/8.4	6.4/7.0	0.05/CF	17	1.1/30/6	As above	As above	1.5/1.1	0.02/24	
	D	Tonalide	0.5/8.4	6.4/7.0	0.05/CF	17	1.1/30/6	As above	As above	0.3/0.2	0.004/26	
Lab/ Secondary	D	Methyl dihydro-jasmonate	0.5/20	18/7.0	0.05/CF	6.0	1.6/36/3	Soilless system/	0.2/ (-100/ -126)	7.3/1.5	0.3/79	Hijosa-Valsero et al. [5]/Spain

Wetland type/Scale /type of treatment	WT	PCPs	Depth (m)/ Area (m^2 PE^{-1})	T (°C) /pH	HLR (m^3 m^{-2} d^{-1})/ OM	OLR (g COD m^{-2} d^{-1})	HRT (days) /SA/ED (months)	Filter media (mm)/Plants	Effluent DO (mg L^{-1})/ ORP (Inf/Eff) (mV)	Influent/ Effluent conc. (μg L^{-1})	Removal rate (mg m^{-2} d^{-1})/ Removal (%)	Author/ Country
								Typha angustifolia				
	D	Galaxolide	0.5/20	18/7.0	0.05/CF	6.0	1.6/36/3	As above	As above	3.8/0.4	0.2/89	
	D	Tonalide	0.5/20	18/7.0	0.05/CF	6.0	1.6/36/3	As above	As above	0.3/0.1	0.01/76	
Lab/ Secondary	D	Methyl dihydro-jasmonate	0.5/20	19/6.8	0.05/CF	6.0	1.2/36/3	25 cm Siliceous gravel (4.0)/ *Typha angustifolia*	0.2/ (-100/ -201)	7.3/1.5	0.3/79	Hijosa-Valsero et al. [5]/Spain
	D	Galaxolide	0.5/20	19/6.8	0.05/CF	6.0	1.2/36/3	As above	As above	3.8/1.3	0.1/66	
	D	Tonalide	0.5/20	19/6.8	0.05/CF	6.0	1.2/36/3	As above	As above	0.3/0.1	0.01/68	
Lab/ Secondary	D	Methyl dihydro-jasmonate	0.5/20	20/6.8	0.05/CF	6.0	1.6/36/3	Soilless system/ *Phragmites australis*	0.2/ (-100/ -107)	7.3/0.7	0.3/90	Hijosa-Valsero et al. [5]/Spain
	D	Galaxolide	0.5/20	20/6.8	0.05/CF	6.0	1.6/36/3	As above	As above	3.8/0.8	0.1/80	
	D	Tonalide	0.5/20	20/6.8	0.05/CF	6.0	1.6/36/3	As above	As above	0.3/0.04	0.01/84	
Lab/ Primary	S	Triclosan	0.5/7.0	NA/NA	0.3/NA	17	5/NA/ NA	25 cm Sand (0-3)/*Cattail*	NA/NA	60/1.0	17/98	Liu et al. [6]/China
Lab/ Primary	S	Triclosan	0.5/7.0	NA/NA	0.3/NA	17	5/NA/ NA	As above/ *Hornwort*	NA/NA	60/1.3	17/98	Liu et al. [6]/China
Lab/ Primary	S	Triclosan	0.5/7.0	NA/NA	0.3/NA	17	5/NA/ NA	As above/ *Lemna minor*	NA/NA	60/0.8	17/99	Liu et al. [6]/China
Lab/ Primary	S	N,N-diethyl-meta-toluamide	0.7/27	NA/7.4	0.02/CF	5.0	7/NA/4	NA/*Spirodela polyrhiza*	6.0/NA/ -63	25/25	0.0/0.0	Li et al. [7]/UK
	S	Triclosan	0.7/27	NA/7.4	0.02/CF	5.0	7/NA/4	As above	As above	25/1.5	0.4/94	
Pilot/ Secondary	D	Methylparaben	1.0/1.3	NA/NA	0.1/CF	73	21/NA/ NA	NA/*Landoltia punctata*	NA/NA	157/15	17/91	Anjos et al. [8]/Brazil
	D	Propylparaben	1.0/1.3	NA/NA	0.1/CF	73	21/NA/ NA	As above	NA/NA	149/57	11/61	
Pilot/ Secondary	D	Methylparaben	1.0/1.3	NA/NA	0.1/CF	73	21/NA/ NA	NA/*Lemna minor*	NA/NA	157/18	17/89	Anjos et al. [8]/Brazil

Wetland type/Scale /type of treatment	WT	PCPs	Depth (m)/ Area (m² PE⁻¹)	T (°C) /pH	HLR (m³ m⁻² d⁻¹)/ OM	OLR (g COD m⁻² d⁻¹)	HRT (days) /SA/ED (months)	Filter media (mm)/Plants	Effluent DO (mg L⁻¹)/ ORP (Inf/Eff) (mV)	Influent/ Effluent conc. (µg L⁻¹)	Removal rate (mg m⁻² d⁻¹)/ Removal (%)	Author/ Country
	D	Propylparaben	1.0/1.3	NA/NA	0.1/CF	73	21/NA/ NA	As above	NA/NA	149/16	16/89	
**Aerated FWSCW**												
Lab/ Primary	S	N,N-diethyl-meta-toluamide	0.7/27	NA/8.0	0.02/CF	5.0	7/NA/4	NA/*Spirodela polyrhiza*	NA/NA/ +14	25/17	0.0/4.2	Li et al. [7]/UK
	S	Triclosan	0.7/27	NA/8.0	0.02/CF	5.0	7/NA/4	As above	As above	25/0.3	0.4/99	

Note: Free water surface flow constructed wetland (FWSCW); Wastewater type (WT); Domestic (D); Synthetic (S); Personal care products (PCPs); Temperature (T); Hydraulic loading rate (HLR); Operational mode (OM); Intermittently fed (IF); Continuously fed (CF); Organic loading rate (OLR); Chemical oxygen demand (COD); Hydraulic retention time (HRT); System age (SA); Experiment duration (ED); Dissolved oxygen (DO); Oxidation-reduction potential (ORP); Not available (NA).

Domestic (55%) and Industrial discharge (45%) (*); The Population equivalent (PE) is calculated based on the common relation 1 PE = 60 g BOD d^{-1}. BOD values were approximated using the ratio COD/BOD = 2 in the studies where BOD was not reported [6-8], and COD values were approximated using the ratio COD = 2BOD in the studies where COD was not reported [5].

Table S2. The performance of HFCW for personal care products removal.

Wetland type/Scale/ type of treatment	WT	PCPs	Depth (m)/ Area (m^2 PE^{-1})	T (°C) /pH	HLR (m^3 m^{-2} d^{-1})/ OM	OLR (g COD m^{-2} d^{-1})	HRT (days) /SA/ED (months)	Filter media (mm)/Plants	Effluent DO (mg L^{-1})/ ORP (Inf/Eff) (mV)	Influent/ Effluent conc. (μg L^{-1})	Removal rate (mg m^{-2} d^{-1})/ Removal (%)	Author/ Country
Lab/ Secondary	D	Methyl dihydro-jasmonate	0.5/18	18/6.5	0.05/CF	5.0	2.5/17/3	45 cm Siliceous gravel (4.0)/ *Phragmites australis*	0.5/+26/ (+26/+22)	4.0/0.1	0.2/98	Hijosa-Valsero et al. [3]/Spain
	D	Galaxolide	0.5/18	18/6.5	0.05/CF	5.0	2.5/17/3	As above	As above	1.2/0.5	0.04/62	
	D	Tonalide	0.5/18	18/6.5	0.05/CF	5.0	2.5/17/3	As above	As above	0.4/0.2	0.01/62	
Lab/ Secondary	D	Methyl dihydro-jasmonate	0.5/18	19/7.4	0.05/CF	5.0	2.6/17/3	45 cm Siliceous gravel (4.0) /Unplanted	0.3/+26/ (-6/-91)	4.0/0.6	0.2/85	Hijosa-Valsero et al. [3]/Spain
	D	Galaxolide	0.5/18	19/7.4	0.05/CF	5.0	2.6/17/3	As above	As above	1.2/0.8	0.02/30	
	D	Tonalide	0.5/18	19/7.4	0.05/CF	5.0	2.6/17/3	As above	As above	0.4/0.3	0.007/37	
Lab/ Secondary	D	Methyl dihydro-jasmonate	0.5/6.0	5.6/6.9	0.05/CF	25	2.5/11/3	45 cm Siliceous gravel (4.0)/ *Phragmites australis*	0.5/-6/ (+39/+24)	12/3.0	0.4/75	Hijosa-Valsero et al. [3]/Spain
	D	Galaxolide	0.5/6.0	5.6/6.9	0.05/CF	25	2.5/11/3	As above	As above	0.9/0.8	0.004/10	
	D	Tonalide	0.5/6.0	5.6/6.9	0.05/CF	25	2.5/11/3	As above	As above	0.4/0.3	0.004/20	
Lab/ Secondary	D	Methyl dihydro-jasmonate	0.5/6.0	5.4/7.4	0.05/CF	25	2.6/11/3	45 cm Siliceous gravel (4.0) /Unplanted	0.6/-6/ (+46/+11)	12/6.0	0.3/50	Hijosa-Valsero et al. [3]/Spain
Lab/ Secondary	D	Methyl dihydro-jasmonate	0.5/18	18/6.5	0.05/CF	6.0	2.2/36/9	45 cm Siliceous gravel (4.0)/ *Phragmites australis*	0.6 (0.6/0.5)/ +2.7/(+47/ -3.3)	5.8/0.7	0.3/89	Reyes-Contreras et al. [4]/Spain
	D	Galaxolide	0.5/18	18/6.5	0.05/CF	6.0	2.2/36/9	As above	As above	2.1/0.6	0.07/70	
	D	Tonalide	0.5/18	18/6.5	0.05/CF	6.0	2.2/36/9	As above	As above	0.4/0.1	0.01/68	

Wetland type/Scale/ type of treatment	WT	PCPs	Depth (m)/ Area (m^2 PE^{-1})	T (°C) /pH	HLR (m^3 m^{-2} d^{-1})/ OM	OLR (g COD m^{-2} d^{-1})	HRT (days) /SA/ED (months)	Filter media (mm)/Plants	Effluent DO (mg L^{-1})/ ORP (Inf/Eff) (mV)	Influent/ Effluent conc. (µg L^{-1})	Removal rate (mg m^{-2} d^{-1})/ Removal (%)	Author/ Country
Lab/ Secondary	D	Methyl dihydro-jasmonate	0.5/8.4	5.6/6.9	0.05/CF	17	2.2/30/6	45 cm Siliceous gravel (4.0)/ *Phragmites australis*	0.6 (0.8/0.5)/ -41/(+14/ -55)	6.6/4.7	0.1/29	Reyes-Contreras et al. [4]/Spain
	D	Galaxolide	0.5/8.4	5.6/6.9	0.05/CF	17	2.2/30/6	As above	As above	1.5/1.3	0.01/12	
	D	Tonalide	0.5/8.4	5.6/6.9	0.05/CF	17	2.2/30/6	As above	As above	0.3/0.4	NR	
Lab/ Secondary	D	Methyl dihydro-jasmonate	0.5/18	17/7.4	0.05/CF	6.0	0.7/36/9	45 cm Siliceous gravel (4.0)/ Unplanted	0.5 (0.5/0.4)/ +2.7/(-5.7 /-83)	5.8/1.4	0.2/76	Reyes-Contreras et al. [4]/Spain
	D	Galaxolide	0.5/18	17/7.4	0.05/CF	6.0	0.7/36/9	As above	As above	2.1/1.7	0.02/19	
	D	Tonalide	0.5/18	17/7.4	0.05/CF	6.0	0.7/36/9	As above	As above	0.4/0.36	0.004/16	
Lab/ Secondary	D	Methyl dihydro-jasmonate	0.5/8.4	5.4/7.4	0.05/CF	17	0.7/30/6	45 cm Siliceous gravel (4.0)/ Unplanted	0.7	6.6/6.0	0.03/8.5	Reyes-Contreras et al. [4]/Spain
	D	Galaxolide	0.5/8.4	5.4/7.4	0.05/CF	17	0.7/30/6	As above	(0.8/0.5)/	1.5/1.7	NR	
	D	Tonalide	0.5/8.4	5.4/7.4	0.05/CF	17	0.7/30/6	As above	-41/(+8/	0.3/0.5	NR	
Pilot/ Secondary	D	Galaxolide	0.5/8.4	NA/7.6	0.03/IF	14	5.5/NA/6	Gravel (8-32)/ *Phragmites australis*	0.3/-91/+8	2.1/1.8	0.01/14	Carranza-Diaz et al. [9]/ Germany
	D	Tonalide	0.5/8.4	NA/7.6	0.03/IF	14	5.5/NA/6	As above	As above	0.9/0.7	0.01/20	
	D	Triclosan	0.5/8.4	NA/7.6	0.03/IF	14	5.5/NA/6	As above	As above	1.0/0.9	0.003/8.0	
Pilot/ Secondary	D	Galaxolide	0.5/8.4	NA/7.7	0.03/IF	14	5.5/NA/6	As above/ Unplanted	0.8/-91/ -84	2.1/2.0	0.004/5.0	Carranza-Diaz et al. [9]/ Germany
	D	Tonalide	0.5/8.4	NA/7.7	0.03/IF	14	5.5/NA/6	As above	As above	0.9/0.8	0.003/9.0	
	D	Triclosan	0.5/8.4	NA/7.7	0.03/IF	14	5.5/NA/6	As above	As above	1.0/0.95	0.001/2.0	

Wetland type/Scale/ type of treatment	WT	PCPs	Depth (m)/ Area (m² PE⁻¹)	T (°C) /pH	HLR (m³ m⁻² d⁻¹)/ OM	OLR (g COD m⁻² d⁻¹)	HRT (days) /SA/ED (months)	Filter media (mm)/Plants	Effluent DO (mg L⁻¹)/ ORP (Inf/Eff) (mV)	Influent/ Effluent conc. (μg L⁻¹)	Removal rate (mg m⁻² d⁻¹)/ Removal (%)	Author/ Country
Lab/ Primary	S	Triclosan	0.5/6.7	NA/NA	0.3/IF	18	5/5/1	25 cm (0-3)/ *Typha angustifolia*	NA/NA	60/5.1	16/91	Zhao et al. [10]/China
Lab/ Primary	S	Triclosan	0.5/6.7	NA/NA	0.3/IF	18	5/5/1	25 cm (0-3)/ *Salvinia natans*	NA/NA	60/8.0	15/87	Zhao et al. [10]/China
Lab/ Primary	S	Triclosan	0.5/6.7	NA/NA	0.3/IF	18	5/5/1	25 cm (0-3)/ *Hydrilla. verticillata*	NA/NA	60/11	14/81	Zhao et al. [10]/China
Full/ Secondary	D	Triclosan	0.8/5.0	NA/NA	0.03	13	6.5/120/6	80 cm Gravel (4-8)/ *Phragmites australis*	NA/NA	0.1/0.01	0.003/91	Chen et al. [11]/Czech Republic
Full/ Secondary	D	Triclosan	0.8/9.4	NA/NA	0.02	3.4	13/36/6	80 cm Gravel (4-8)/*Phalaris arundinacea*	NA/NA	0.1/0.01	0.003/91	Chen et al. [11]/Czech Republic
Full/ Secondary	D	Triclosan	0.8/6.6	NA/NA	0.04	7.3	5.4/144/6	80 cm Gravel (4-16)/ *Phragmites australis*	NA/NA	0.1/0.01	0.003/91	Chen et al. [11]/Czech Republic
Pilot/ Secondary	D	Galaxolide	0.47/2.0	25/7.4	0.1/CF	61	NA/NA/ 7	3.9 cm Gravel (5) 1.9 cm Tezontle (5) 3.8 cm Tezontle (5)/ *Typha latiffolia, Phragmites australis, Cyperus papyrus*	1.4/NA/ -325	4.1/0.9	0.3/79	Herrera-Cárdenas et al. [12]/ México

Wetland type/Scale/ type of treatment	WT	PCPs	Depth (m)/ Area (m² PE⁻¹)	T (°C) /pH	HLR (m³ m⁻² d⁻¹)/ OM	OLR (g COD m⁻² d⁻¹)	HRT (days) /SA/ED (months)	Filter media (mm)/Plants	Effluent DO (mg L⁻¹)/ ORP (Inf/Eff) (mV)	Influent/ Effluent conc. (μg L⁻¹)	Removal rate (mg m⁻² d⁻¹)/ Removal (%)	Author/ Country
	D	Tonalide	0.47/2.0	25/7.4	0.1/CF	61	NA/NA/ 7	As above	As above	0.5/0.1	0.05/86	
	D	Methyl dihydro-jasmonate	0.47/2.0	25/7.4	0.1/CF	61	NA/NA/ 7	As above	As above	11/3.4	0.8/70	
Lab/ Secondary	D	Methyl dihydro-jasmonate	0.5/20	20/6.5	0.05/CF	6.0	3/36/3	45 cm Siliceous gravel (4.0)/ *Phragmites australis*	0.2/ (-100/ -102)	7.3/1.1	0.3/85	Hijosa-Valsero et al. [5]/Spain
	D	Galaxolide	0.5/20	20/6.5	0.05/CF	6.0	3/36/3	As above	As above	3.8/1.1	0.1/72	
	D	Tonalide	0.5/20	20/6.5	0.05/CF	6.0	3/36/3	As above	As above	0.3/0.1	0.01/72	
Lab/ Secondary	D	Methyl dihydro-jasmonate	0.5/20	19/7.2	0.05/CF	6.0	1.7/36/3	45 cm Siliceous gravel (4.0)/ Unplanted	0.2/ (-100/ -155)	7.3/1.2	0.3/83	Hijosa-Valsero et al. [5]/Spain
	D	Galaxolide	0.5/20	19/7.2	0.05/CF	6.0	1.7/36/3	As above	As above	3.8/2.2	0.08/42	
	D	Tonalide	0.5/20	19/7.2	0.05/CF	6.0	1.7/36/3	As above	As above	0.3/0.2	0.004/36	
Full/ Tertiary	*	Galaxolide	0.7/8.6	18/NA	0.003/IF	14	NA/120/1	Gravel (8-25) Granitic gravel (6-25)/ *Phragmites australis*	0.4/NA	1.4/0.8	0.001/42	Matamoros et al. [13]/ Spain
	*	Oxybenzone	0.7/8.6	18/NA	0.003/IF	14	NA/120/1	As above	0.4/NA	1.1/0.3	0.002/74	
	*	Methylparaben	0.7/8.6	18/NA	0.003/IF	14	NA/120/1	As above	0.4/NA	0.7/0.6	0.0003/16	
	*	Triclosan	0.7/8.6	18/NA	0.003/IF	14	NA/120/1	As above	0.4/NA	0.7/0.2	0.001/67	
	*	Tributyl phosphate	0.7/8.6	18/NA	0.003/IF	14	NA/120/1	As above	0.4/NA	0.5/0.2	0.001/61	
	*	Tonalide	0.7/8.6	18/NA	0.003/IF	14	NA/120/1	As above	0.4/NA	0.5/0.3	0.001/41	
	*	Tris (2-chloroethyl) phosphate	0.7/8.6	18/NA	0.003/IF	14	NA/120/1	As above	0.4/NA	0.5/0.4	0.0004/28	

Wetland type/Scale/ type of treatment	WT	PCPs	Depth (m)/ Area (m² PE⁻¹)	T (°C) /pH	HLR (m³ m⁻² d⁻¹)/ OM	OLR (g COD m⁻² d⁻¹)	HRT (days) /SA/ED (months)	Filter media (mm)/Plants	Effluent DO (mg L⁻¹)/ ORP (Inf/Eff) (mV)	Influent/ Effluent conc. (µg L⁻¹)	Removal rate (mg m⁻² d⁻¹)/ Removal (%)	Author/ Country
	*	Methyl dihydro-jasmonate	0.7/8.6	18/NA	0.003/IF	14	NA/120/1	As above	0.4/NA	0.3/0.1	0.0004/56	
	*	Triphenyl phosphate	0.7/8.6	18/NA	0.003/IF	14	NA/120/1	As above	0.4/NA	0.1/0.01	0.0002/82	
Full/ Tertiary	*	Galaxolide	0.7/3.2	23/NA	0.003/IF	38	NA/120/1	As above	0.5/NA	1.4/0.6	0.002/59	Matamoros et al. [13]/ Spain
	*	Oxybenzone	0.7/3.2	23/NA	0.003/IF	38	NA/120/1	As above	0.5/NA	1.1/0.2	0.002/85	
	*	Methylparaben	0.7/3.2	23/NA	0.003/IF	38	NA/120/1	As above	0.5/NA	0.7/0.3	0.001/60	
	*	Triclosan	0.7/3.2	23/NA	0.003/IF	38	NA/120/1	As above	0.5/NA	0.7/0.2	0.001/73	
	*	Tributyl phosphate	0.7/3.2	23/NA	0.003/IF	38	NA/120/1	As above	0.5/NA	0.5/0.3	0.0004/34	
	*	Tonalide	0.7/3.2	23/NA	0.003/IF	38	NA/120/1	As above	0.5/NA	0.5/0.2	0.001/51	
	*	Tris (2-chloroethyl) phosphate	0.7/3.2	23/NA	0.003/IF	38	NA/120/1	As above	0.5/NA	0.5/0.4	0.0002/19	
	*	Methyl dihydro-jasmonate	0.7/3.2	23/NA	0.003/IF	38	NA/120/1	As above	0.5/NA	0.3/0.1	0.0004/60	
	*	Triphenyl phosphate	0.7/3.2	23/NA	0.003/IF	38	NA/120/1	As above	0.5/NA	0.1/0.02	0.0002/75	
Pilot/ Secondary	D	Acesulfame	0.5/5.5	21/NA	0.03/CF	22	5.5/60/12	Gravel (8-16)/ *Phragmites australis*	3.0/-277/ -228	19/19	0.0/0.0	Kahl et al. [14]/ Germany
Full/ Tertiary	L	N,N-diethyl-meta-toluamide	NA/NA	NA/NA	0.01/NA	1.0	23/NA/12	NA/ *Phragmites australis*	NA/NA	1.5/0.03	0.02/98	Yi et al. [15]/ Singapore
Full/ Primary	D	Triclosan	NA/5.3	NA/NA	NA/NA	NA	NA/264/ 12	Crushed rock (4-8)/ *Phragmites*	NA/NA	0.6/0.1	NA/86	Vymazal et al. [16]/

Wetland type/Scale/ type of treatment	WT	PCPs	Depth (m)/ Area (m² PE⁻¹)	T (°C) /pH	HLR (m³ m⁻² d⁻¹)/ OM	OLR (g COD m⁻² d⁻¹)	HRT (days) /SA/ED (months)	Filter media (mm)/Plants	Effluent DO (mg L⁻¹)/ ORP (Inf/Eff) (mV)	Influent/ Effluent conc. (µg L⁻¹)	Removal rate (mg m⁻² d⁻¹)/ Removal (%)	Author/ Country
								australis				Czech Republic
	D	Triclocarban	NA/5.3	NA/NA	NA/NA	NA	As above	As above	NA/NA	0.1/0.01	NA/86	
Full/ Primary	D	Triclosan	NA/11	NA/NA	NA/NA	NA	NA/252/ 12	Gravel (4-8)/ *Phragmites australis*	NA/NA	0.2/0.1	NA/53	Vymazal et al. [16]/ Czech Republic
	D	Triclocarban	NA/11	NA/NA	NA/NA	NA	As above	As above	NA/NA	0.03/0.01	NA/67	
Full/ Primary	D	Triclosan	NA/5.5	NA/NA	NA/NA	NA	NA/192/ 12	Gravel (4-8)/ *Phragmites australis*, *Phragmites arundinacea*	NA/NA	0.2/0.1	NA/47	Vymazal et al. [16]/ Czech Republic
	D	Triclocarban	NA/5.5	NA/NA	NA/NA	NA	As above	As above	NA/NA	0.3/0.01	NA/96	
Full/ Primary	D	Triclosan	NA/4.6	NA/NA	NA/NA	NA	NA/120/ 12	Gravel (8-16)/ *Phragmites australis*, *Phragmites arundinacea*	NA/NA	0.5/0.1	NA/73	Vymazal et al. [16]/ Czech Republic
	D	Triclocarban	NA/4.6	NA/NA	NA/NA	NA	As above	As above	NA/NA	0.05/0.01	NA/74	
Lab/ Primary	S	Methyl dihydro-jasmonate	0.33/2.3	NA/NA	0.25/IF	51	1.0/NA/ NA	33 cm Volcanic gravel (4.0)/ *Phragmites australis*	NA/NA	4.8/2.8	0.5/42	Salcedo et al. [17]/ Mexico
	S	Galaxolide	0.33/2.3	NA/NA	0.25/IF	51	As above	As above	NA/NA	2.7/1.1	0.4/59	
Lab/ Primary	S	Methyl dihydro-jasmonate	0.33/2.3	NA/NA	0.25/IF	51	3.0/NA/ NA	33 cm Volcanic gravel (4.0)/ *Phragmites australis*	NA/NA	4.8/1.5	0.8/70	Salcedo et al. [17]/ Mexico

Wetland type/Scale/ type of treatment	WT	PCPs	Depth (m)/ Area (m^2 PE^{-1})	T (°C) /pH	HLR (m^3 m^{-2} d^{-1})/ OM	OLR (g COD m^{-2} d^{-1})	HRT (days) /SA/ED (months)	Filter media (mm)/Plants	Effluent DO (mg L^{-1})/ ORP (Inf/Eff) (mV)	Influent/ Effluent conc. (µg L^{-1})	Removal rate (mg m^{-2} d^{-1})/ Removal (%)	Author/ Country
	S	Galaxolide	0.33/2.3	NA/NA	0.25/IF	51	As above	As above	NA/NA	2.7/0.4	0.6/86	
Lab/ Primary	S	Methyl dihydro-jasmonate	0.33/2.3	NA/NA	0.25/IF	51	5.0/NA/ NA	33 cm Volcanic gravel (4.0)/ *Phragmites australis*	NA/NA	4.8/0.8	1.0/84	Salcedo et al. [17]/ Mexico
	S	Galaxolide	0.33/2.3	NA/NA	0.25/IF	51	As above	As above	NA/NA	2.7/0.2	0.6/93	
Lab/ Primary	S	Methyl dihydro-jasmonate	0.33/2.3	NA/NA	0.25/IF	51	1.0/NA/ NA	33 cm River gravel (10)/ *Phragmites australis*	NA/NA	4.8/2.2	0.6/54	Salcedo et al. [17]/ Mexico
	S	Galaxolide	0.33/2.3	NA/NA	0.25/IF	51	As above	As above	NA/NA	2.7/0.8	0.5/71	
Lab/ Primary	S	Methyl dihydro-jasmonate	0.33/2.3	NA/NA	0.25/IF	51	3.0/NA/ NA	33 cm River gravel (10)/ *Phragmites australis*	NA/NA	4.8/1.3	0.9/73	Salcedo et al. [17]/ Mexico
	S	Galaxolide	0.33/2.3	NA/NA	0.25/IF	51	As above	As above	NA/NA	2.7/0.3	0.6/88	
Lab/ Primary	S	Methyl dihydro-jasmonate	0.33/2.3	NA/NA	0.25/IF	51	5.0/NA/ NA	33 cm River gravel (10)/ *Phragmites australis*	NA/NA	4.8/0.7	1.0/86	Salcedo et al. [17]/ Mexico
	S	Galaxolide	0.33/2.3	NA/NA	0.25/IF	51	As above	As above	NA/NA	2.7/0.2	0.6/92	
Lab/ Primary	S	Methyl dihydro-jasmonate	0.33/2.3	NA/NA	0.25/IF	51	1.0/NA/ NA	33 cm Volcanic gravel (4.0)/ *Typha latifolia*	NA/NA	4.8/0.9	1.0/81	Salcedo et al. [17]/ Mexico
	S	Galaxolide	0.33/2.3	NA/NA	0.25/IF	51	As above	As above	NA/NA	2.7/1.6	0.3/41	
Lab/ Primary	S	Methyl dihydro-jasmonate	0.33/2.3	NA/NA	0.25/IF	51	3.0/NA/ NA	33 cm Volcanic gravel (4.0)/ *Typha latifolia*	NA/NA	4.8/0.4	1.1/93	Salcedo et al. [17]/ Mexico
	S	Galaxolide	0.33/2.3	NA/NA	0.25/IF	51	As above	As above	NA/NA	2.7/1.4	0.3/49	

Wetland type/Scale/ type of treatment	WT	PCPs	Depth (m)/ Area (m² PE⁻¹)	T (°C) /pH	HLR (m³ m⁻² d⁻¹)/ OM	OLR (g COD m⁻² d⁻¹)	HRT (days) /SA/ED (months)	Filter media (mm)/Plants	Effluent DO (mg L⁻¹)/ ORP (Inf/Eff) (mV)	Influent/ Effluent conc. (µg L⁻¹)	Removal rate (mg m⁻² d⁻¹)/ Removal (%)	Author/ Country
Lab/ Primary	S	Methyl dihydro-jasmonate	0.33/2.3	NA/NA	0.25/IF	51	5.0/NA/ NA	33 cm Volcanic gravel (4.0)/ *Typha latifolia*	NA/NA	4.8/0.2	1.2/96	Salcedo et al. [17]/ Mexico
	S	Galaxolide	0.33/2.3	NA/NA	0.25/IF	51	As above	As above	NA/NA	2.7/0.4	0.6/84	
Lab/ Primary	S	Methyl dihydro-jasmonate	0.33/2.3	NA/NA	0.25/IF	51	1.0/NA/ NA	33 cm River gravel (10)/ *Typha latifolia*	NA/NA	4.8/1.2	0.9/76	Salcedo et al. [17]/ Mexico
	S	Galaxolide	0.33/2.3	NA/NA	0.25/IF	51	As above	As above	NA/NA	2.7/1.8	0.2/34	
Lab/ Primary	S	Methyl dihydro-jasmonate	0.33/2.3	NA/NA	0.25/IF	51	3.0/NA/ NA	33 cm River gravel (10)/ *Typha latifolia*	NA/NA	4.8/0.3	1.1/95	Salcedo et al. [17]/ Mexico
	S	Galaxolide	0.33/2.3	NA/NA	0.25/IF	51	As above	As above	NA/NA	2.7/1.4	0.3/47	
Lab/ Primary	S	Methyl dihydro-jasmonate	0.33/2.3	NA/NA	0.25/IF	51	5.0/NA/ NA	33 cm River gravel (10)/ *Typha latifolia*	NA/NA	4.8/0.2	1.2/96	Salcedo et al. [17]/ Mexico
	S	Galaxolide	0.33/2.3	NA/NA	0.25/IF	51	As above	As above	NA/NA	2.7/0.3	0.6/87	
Lab/ Primary	S	Methyl dihydro-jasmonate	0.33/2.3	NA/NA	0.25/IF	51	1.0/NA/ NA	33 cm Volcanic gravel (4.0)/ *Cyperus papyrus*	NA/NA	4.8/2.1	0.7/56	Salcedo et al. [17]/ Mexico
	S	Galaxolide	0.33/2.3	NA/NA	0.25/IF	51	As above	As above	NA/NA	2.7/1.2	0.4/54	
Lab/ Primary	S	Methyl dihydro-jasmonate	0.33/2.3	NA/NA	0.25/IF	51	3.0/NA/ NA	33 cm Volcanic gravel (4.0)/ *Cyperus papyrus*	NA/NA	4.8/0.7	1.0/85	Salcedo et al. [17]/ Mexico
	S	Galaxolide	0.33/2.3	NA/NA	0.25/IF	51	As above	As above	NA/NA	2.7/0.7	0.5/73	
Lab/ Primary	S	Methyl dihydro-jasmonate	0.33/2.3	NA/NA	0.25/IF	51	5.0/NA/ NA	33 cm Volcanic gravel (4.0)/	NA/NA	4.8/0.3	1.1/93	Salcedo et al. [17]/ Mexico

Wetland type/Scale/ type of treatment	W T	PCPs	Depth (m)/ Area (m^2 PE^{-1})	T (°C) /pH	HLR (m^3 m^{-2} d^{-1})/ OM	OLR (g COD m^{-2} d^{-1})	HRT (days) /SA/ED (months)	Filter media (mm)/Plants	Effluent DO (mg L^{-1})/ ORP (Inf/Eff) (mV)	Influent/ Effluent conc. (µg L^{-1})	Removal rate (mg m^{-2} d^{-1})/ Removal (%)	Author/ Country
								Cyperus papyrus				
	S	Galaxolide	0.33/2.3	NA/NA	0.25/IF	51	As above	As above	NA/NA	2.7/0.5	0.6/83	
Lab/ Primary	S	Methyl dihydro-jasmonate	0.33/2.3	NA/NA	0.25/IF	51	1.0/NA/ NA	33 cm River gravel (10)/ *Cyperus papyrus*	NA/NA	4.8/2.6	0.6/47	Salcedo et al. [17]/ Mexico
	S	Galaxolide	0.33/2.3	NA/NA	0.25/IF	51	As above	As above	NA/NA	2.7/1.7	0.3/38	
Lab/ Primary	S	Methyl dihydro-jasmonate	0.33/2.3	NA/NA	0.25/IF	51	3.0/NA/ NA	33 cm River gravel (10)/ *Cyperus papyrus*	NA/NA	4.8/0.6	1.1/87	Salcedo et al. [17]/ Mexico
	S	Galaxolide	0.33/2.3	NA/NA	0.25/IF	51	As above	As above	NA/NA	2.7/0.9	0.5/68	
Lab/ Primary	S	Methyl dihydro-jasmonate	0.33/2.3	NA/NA	0.25/IF	51	5.0/NA/ NA	33 cm River gravel (10)/ *Cyperus papyrus*	NA/NA	4.8/0.3	1.1/93	Salcedo et al. [17]/ Mexico
	S	Galaxolide	0.33/2.3	NA/NA	0.25/IF	51	As above	As above	NA/NA	2.7/0.3	0.6/90	
Lab/ Primary	S	Methyl dihydro-jasmonate	0.33/2.3	NA/NA	0.25/IF	51	1.0/NA/ NA	33 cm Volcanic gravel (4.0)/ Unplanted	NA/NA	4.8/2.5	0.6/49	Salcedo et al. [17]/ Mexico
	S	Galaxolide	0.33/2.3	NA/NA	0.25/IF	51	As above	As above	NA/NA	2.7/1.8	0.2/33	
Lab/ Primary	S	Methyl dihydro-jasmonate	0.33/2.3	NA/NA	0.25/IF	51	3.0/NA/ NA	33 cm Volcanic gravel (4.0)/ Unplanted	NA/NA	4.8/0.9	1.0/81	Salcedo et al. [17]/ Mexico
	S	Galaxolide	0.33/2.3	NA/NA	0.25/IF	51	As above	As above	NA/NA	2.7/1.8	0.2/32	
Lab/ Primary	S	Methyl dihydro-jasmonate	0.33/2.3	NA/NA	0.25/IF	51	5.0/NA/ NA	33 cm Volcanic gravel (4.0)/ Unplanted	NA/NA	4.8/0.7	1.0/86	Salcedo et al. [17]/ Mexico

Wetland type/Scale/ type of treatment	WT	PCPs	Depth (m)/ Area (m^2 PE^{-1})	T (°C) /pH	HLR (m^3 m^{-2} d^{-1})/ OM	OLR (g COD m^{-2} d^{-1})	HRT (days) /SA/ED (months)	Filter media (mm)/Plants	Effluent DO (mg L^{-1})/ ORP (Inf/Eff) (mV)	Influent/ Effluent conc. (µg L^{-1})	Removal rate (mg m^{-2} d^{-1})/ Removal (%)	Author/ Country
	S	Galaxolide	0.33/2.3	NA/NA	0.25/IF	51	As above	As above	NA/NA	2.7/1.3	0.3/52	
Lab/ Primary	S	Methyl dihydro-jasmonate	0.33/2.3	NA/NA	0.25/IF	51	1.0/NA/ NA	33 cm River gravel (10)/ Unplanted	NA/NA	4.8/2.7	0.5/45	Salcedo et al. [17]/ Mexico
	S	Galaxolide	0.33/2.3	NA/NA	0.25/IF	51	As above	As above	NA/NA	2.7/2.1	0.1/22	
Lab/ Primary	S	Methyl dihydro-jasmonate	0.33/2.3	NA/NA	0.25/IF	51	3.0/NA/ NA	33 cm River gravel (10)/ Unplanted	NA/NA	4.8/1.0	1.0/79	Salcedo et al. [17]/ Mexico
	S	Galaxolide	0.33/2.3	NA/NA	0.25/IF	51	As above	As above	NA/NA	2.7/1.7	0.2/35	
Lab/ Primary	S	Methyl dihydro-jasmonate	0.33/2.3	NA/NA	0.25/IF	51	5.0/NA/ NA	33 cm River gravel (10)/ Unplanted	NA/NA	4.8/0.6	1.1/88	Salcedo et al. [17]/ Mexico
	S	Galaxolide	0.33/2.3	NA/NA	0.25/IF	51	As above	As above	NA/NA	2.7/1.3	0.3/50	
Full/ Tertiary	D	Oxybenzone	NA/NA	16/8.6	3.4/NA	NA	14/NA/2	Steel slag/ *Phragmites australis*	6.0/NA	0.05/0.06	NR	Petrie et al. [18]/UK
	D	Sulisobenzone	NA/NA	16/8.6	3.4/NA	NA	14/NA/2	As above	6.0/NA	4.2/3.8	1.2/8.7	
	D	Methylparaben	NA/NA	16/8.6	3.4/NA	NA	14/NA/2	As above	6.0/NA	0.024/ 0.023	0.004/5.0	
	D	Propylparaben	NA/NA	16/8.6	3.4/NA	NA	14/NA/2	As above	6.0/NA	0.006/ 0.008	NR	
	D	Triclosan	NA/NA	16/8.6	3.4/NA	NA	14/NA/2	As above	6.0/NA	0.1/0.1	NR	
Full/ Tertiary	D	Oxybenzone	NA/NA	16/8.0	3.4/NA	NA	14/NA/2	Gravel/ *Phragmites australis*	5.5/NA	0.05/0.03	0.05/32	Petrie et al. [18]/UK
	D	Sulisobenzone	NA/NA	16/8.0	3.4/NA	NA	14/NA/2	As above	5.5/NA	4.2/3.9	1.0/7.3	
	D	Methylparaben	NA/NA	16/8.0	3.4/NA	NA	14/NA/2	As above	5.5/NA	0.024/ 0.02	0.01/16	
	D	Propylparaben	NA/NA	16/8.0	3.4/NA	NA	14/NA/2	As above	5.5/NA	0.006/ 0.006	0.0/1.6	
	D	Triclosan	NA/NA	16/8.0	3.4/NA	NA	14/NA/2	As above	5.5/NA	0.13/0.1	0.08/19	

Wetland type/Scale/ type of treatment	WT	PCPs	Depth (m)/ Area (m^2 PE^{-1})	T (°C) /pH	HLR (m^3 m^{-2} d^{-1})/ OM	OLR (g COD m^{-2} d^{-1})	HRT (days) /SA/ED (months)	Filter media (mm)/Plants	Effluent DO (mg L^{-1})/ ORP (Inf/Eff) (mV)	Influent/ Effluent conc. (μg L^{-1})	Removal rate (mg m^{-2} d^{-1})/ Removal (%)	Author/ Country
Full/ Tertiary	D	Oxybenzone	NA/NA	16/8.2	3.4/NA	NA	14/NA/12	Gravel/ *Phragmites australis*	6.0/NA	0.05/0.07	NR	Petrie et al. [18]/UK
	D	Sulisobenzone	NA/NA	16/8.2	3.4/NA	NA	14/NA/12	As above	6.0/NA	4.2/3.7	1.5/11	
	D	Methylparaben	NA/NA	16/8.2	3.4/NA	NA	14/NA/12	As above	6.0/NA	0.024/ 0.02	0.01/17	
	D	Propylparaben	NA/NA	16/8.2	3.4/NA	NA	14/NA/12	As above	6.0/NA	0.006/ 0.009	NR	
	D	Triclosan	NA/NA	16/8.2	3.4/NA	NA	14/NA/12	As above	6.0/NA	0.13/0.1	0.09/21	
Pilot/ Secondary	D	Acesulfame	0.5/5.3	12/7.2	0.03/CF	23	5.5/60/12	Gravel (8-16)/ *Phragmites australis*	3.0/-270/ -220	19/18	0.03/5.0	Nivala et al. [19]/ Germany
Full/ Tertiary	L	Triclosan	NA/NA	NA/NA	NA/NA	NA	NA/NA/ 1.0	NA/*Typha angustifolia*; *Chrysopogon zizanioide*; *Cyperus papyrus*	NA/NA	0.003/ 0.001	NA/57	Wang et al. [20]/ Singapore
Full/ Tertiary	L	Triclosan	NA/NA	NA/NA	NA/NA	NA	NA/NA/ 1.0	As above	NA/NA	0.003/ 0.002	NA/29	Wang et al. [20]/ Singapore
**Aerated HFCW**												
Pilot/ Secondary	D	Acesulfame	1.0/1.4	21/NA	0.1/CF	87	5.5/60/12	Gravel (8-16)/ *Phragmites australis*	10/-277/ +208	19/4.0	2.1/79	Kahl et al. [14]/ Germany
Pilot/ Secondary	D	Acesulfame	1.0/1.3	12/6.9	0.1/CF	90	5.5/60/12	Gravel (8-16)/ *Phragmites australis*	10/-270/ +183	19/7.2	1.6/62	Nivala et al. [19]/ Germany

Note: Horizontal flow constructed wetland (HFCW); Wastewater type (WT); Domestic (D); Synthetic (S); Landfill leachate (L); Personal care products (PCPs); Temperature (T); Hydraulic loading rate (HLR); Operational mode (OM); Intermittently fed (IF); Continuously fed (CF); Organic loading rate (OLR); Chemical oxygen demand (COD); Hydraulic retention time (HRT); System age (SA); Experiment duration (ED); Dissolved oxygen (DO); Oxidation-reduction potential (ORP); Not available (NA); Not removed (NR).

Domestic (55%) and Industrial discharge (45%) (*); The Population equivalent (PE) is calculated based on the common relation 1 PE = 60 g BOD d^{-1}. BOD values were approximated using the ratio COD/BOD = 2 in the studies where BOD was not reported [10,13], and COD values were approximated using the ratio COD = 2BOD in the studies where COD was not reported [5,12,14,17,19].

Table S3. The performance of VFCW for personal care products removal.

Wetland type/Scale/ type of treatment	WT	PCPs	Depth (m)/ Area (m^2 PE^{-1})	T (°C) /pH	HLR (m^3 m^{-2} d^{-1})/ OM	OLR (g COD m^{-2} d^{-1})	HRT (days) /SA/ED (months)	Filter media (mm)/Plants	Effluent DO (mg L^{-1})/ ORP (Inf/Eff) (mV)	Influent/ Effluent conc. (μg L^{-1})	Removal rate (mg m^{-2} d^{-1})/ Removal (%)	Author/ Country
Pilot/ Secondary	D	Methyl dihydro-jasmonate	1.0/3.5	NA/8.1	0.07/IF	35	0.25/60/2	80 cm Gravel (0.5–4) 20 cm Gravel (8-16)/ *Phragmites australis*	9.0/NA	23/0.2	1.6/99	Matamoros et al. [21]/ Denmark
	D	Hydrocinnamic acid	1.0/3.5	NA/8.1	0.07/IF	35	0.25/60/2	As above	9.0/NA	15/0.2	1.1/99	
	D	Oxybenzone	1.0/3.5	NA/8.1	0.07/IF	35	0.25/60/2	As above	9.0/NA	15/0.4	1.0/97	
	D	Galaxolide	1.0/3.5	NA/8.1	0.07/IF	35	0.25/60/2	As above	9.0/NA	5.6/0.6	0.4/90	
	D	Tonalide	1.0/3.5	NA/8.1	0.07/IF	35	0.25/60/2	As above	9.0/NA	1.0/0.2	0.1/82	
Pilot/ Secondary	D	Tonalide	0.85/NA	19/6.6	0.095/IF	NA	NA/12/2	Sand (1-3)/ *Phragmites australis*	5.5/-263/ +169	0.2/0.04	0.01/78	Ávila et al. [22]/ Germany
	D	Oxybenzone	0.85/NA	19/6.6	0.095/IF	NA	NA/12/2	As above	As above	2.6/0.1	0.2/96	
	D	Triclosan	0.85/NA	19/6.6	0.095/IF	NA	NA/12/2	As above	As above	0.4/0.1	0.04/89	
Pilot/ Secondary	D	Tonalide	0.85/NA	19/7.2	0.095/IF	NA	NA/12/2	Gravel (4-8)/ *Phragmites australis*	3.4/-263/ +98	0.2/0.1	0.01/61	Ávila et al. [22]/ Germany
	D	Oxybenzone	0.85/NA	19/7.2	0.095/IF	NA	NA/12/2	As above	As above	2.6/0.3	0.2/89	
	D	Triclosan	0.85/NA	19/7.2	0.095/IF	NA	NA/12/2	As above	As above	0.4/0.1	0.03/73	
Pilot/ Secondary	D	Acesulfame	0.85/2.1	21/NA	0.09/CF	58	NA/60/12	Gravel (4-8)/ *Phragmites australis*	4.0/-277/ +111	19/19.4	NR	Kahl et al. [14]/ Germany
Pilot/ Primary	D	Triclosan	0.69/NA	NA/NA	0.03/IF	1.0	7.0/NA/ 3.0	15 cm Gravel (20-30) 45 cm of expanded	NA/NA	0.8/0.1	0.02/84	Francini et al. [23]/Italy

Wetland type/Scale/ type of treatment	WT	PCPs	Depth (m)/ Area (m^2 PE^{-1})	T (°C) /pH	HLR (m^3 m^{-2} d^{-1})/ OM	OLR (g COD m^{-2} d^{-1})	HRT (days) /SA/ED (months)	Filter media (mm)/Plants	Effluent DO (mg L^{-1})/ ORP (Inf/Eff) (mV)	Influent/ Effluent conc. (µg L^{-1})	Removal rate (mg m^{-2} d^{-1})/ Removal (%)	Author/ Country
								Agrileca clay (8-20) 5 cm Gravel (20-30)/ *Phragmites australis*				
Pilot/ Primary	D	Triclosan	0.69/NA	NA/NA	0.03/IF	1.0	7.0/NA/ 3.0	15 cm Gravel (20-30) 45 cm of expanded Agrileca clay (8-20) 5 cm Gravel (20-30)/*Salix matsudana*	NA/NA	0.8/0.1	0.02/83	Francini et al. [23]/Italy
Pilot/ Secondary	D	N,N-diethyl-meta-toluamide	0.8/1.6	21/6.9	0.1/IF	40	NA/76/4	10 cm Sand (1-2) 70 cm Gravel (3-8)/ *Phragmites australis*	0.5/+33/ -16	2.4/1.7	0.1/28	Sgroi et al. [24]/Spain
	D	Sucralose	0.8/1.6	21/6.9	0.1/IF	40	NA/76/4	As above	As above	13.1/12.5	0.1/4.4	
Lab/ Primary	S	Triclosan	0.6/NA	NA/NA	0.08/CF	4.0	3.0/5.0/ 3.0	20 cm Washed river sand (0-3.0) 20 cm Birnessite-coated sand (1.0-3.0)	2.4/NA	80/7.2	5.8/91	Xie et al. [25]/China

Wetland type/Scale/ type of treatment	WT	PCPs	Depth (m)/ Area (m² PE⁻¹)	T (°C) /pH	HLR (m³ m⁻² d⁻¹)/ OM	OLR (g COD m⁻² d⁻¹)	HRT (days) /SA/ED (months)	Filter media (mm)/Plants	Effluent DO (mg L⁻¹)/ ORP (Inf/Eff) (mV)	Influent/ Effluent conc. (μg L⁻¹)	Removal rate (mg m⁻² d⁻¹)/ Removal (%)	Author/ Country
Lab/ Primary	S	Triclosan	0.6/NA	NA/NA	0.08/CF	4.0	3.0/5.0/ 3.0	10 cm Gravel (10-30)/ *Phragmites australis* 40 cm Washed river sand (0-3.0)	2.4/NA	80/13	5.4/84	Xie et al. [25]/China
Pilot/ Secondary	D	Acesulfame	0.85/2.0	13/6.6	0.09/CF	61	NA/60/12	10 cm Gravel (10-30)/ *Phragmites australis* Gravel (4-8)/ *Phragmites australis*	5.0/-270/ +108	19/20	NR	Nivala et al. [19]/ Germany
Lab/ Primary	S	Triclosan	0.64/11	20/8.0	0.02/ NA	11	7.0/1.0/16	30 cm Gravel (10-15) 80% Limestone /*Phalaris arundinacea*	8.5/NA	500/0.2	11/100	Button et al. [26]/ Canada
Lab/ Primary	S	Triclosan	0.64/11	20/8.0	0.02/ NA	11	7.0/1.0/16	30 cm Gravel (10-15) 80% Limestone /Unplanted	8.5/NA	500/0.2	11/100	Button et al. [26]/ Canada

Aerated VFCW

Wetland type/Scale/ type of treatment	WT	PCPs	Depth (m)/ Area (m^2 PE^{-1})	T (°C) /pH	HLR (m^3 m^{-2} d^{-1})/ OM	OLR (g COD m^{-2} d^{-1})	HRT (days) /SA/ED (months)	Filter media (mm)/Plants	Effluent DO (mg L^{-1})/ ORP (Inf/Eff) (mV)	Influent/ Effluent conc. (µg L^{-1})	Removal rate (mg m^{-2} d^{-1})/ Removal (%)	Author/ Country
Pilot/ Secondary	D	Tonalide	0.85/NA	19/6.1	0.095/IF	NA	NA/12/2	Gravel (8-16)/ *Phragmites australis*	5.2/-263/ +172	0.2/0.03	0.01/83	Ávila et al. [22]/ Germany
	D	Oxybenzone	0.85/NA	19/6.1	0.095/IF	NA	NA/12/2	As above	As above	2.6/0.2	0.2/91	
	D	Triclosan	0.85/NA	19/6.1	0.095/IF	NA	NA/12/2	As above	As above	0.4/0.1	0.04/86	
Pilot/ Secondary	D	Acesulfame	0.85/2.1	21/NA	0.09/CF	58	NA/60/12	Gravel (8-16)/ *Phragmites australis*	8.0/-277 /+86	19/9.0	0.9/53	Kahl et al. [14]/ Germany
Pilot/ Secondary	D	Acesulfame	0.85/2.0	12/7.0	0.09/CF	61	NA/60/12	Gravel (8-16)/ *Phragmites australis*	8.0/-270 /+88	19/8.7	1.0/54	Nivala et al. [19]/ Germany

Note: Vertical flow constructed wetland (VFCW); Wastewater type (WT); Domestic (D); Synthetic (S); Personal care products (PCPs); Temperature (T); Hydraulic loading rate (HLR); Operational mode (OM); Intermittently fed (IF); Continuously fed (CF); Organic loading rate (OLR); Chemical oxygen demand (COD); Hydraulic retention time (HRT); System age (SA); Experiment duration (ED); Dissolved oxygen (DO); Oxidation-reduction potential (ORP); Not available (NA); Not removed (NR).

The Population equivalent (PE) is calculated based on the common relation 1 PE = 60 g BOD d^{-1}. BOD values were approximated using the ratio COD/BOD = 2 in the studies where BOD was not reported [23,25,26], and COD values were approximated using the ratio COD = 2BOD in the studies where COD was not reported [14,19,21].

Table S4. The performance of HCW for personal care products removal.

Wetland type/Scale/ type of treatment	WT	PCPs	Depth (m)/Area (m^2 PE^{-1})	T (°C) /pH	HLR (m^3 m^{-2} d^{-1})/ OM	OLR (g COD m^{-2} d^{-1})	HRT (days) /SA/ED (months)	Filter media (mm)/Plants	Effluent DO (mg L^{-1})/ ORP (Inf/Eff) (mV)	Influent/ Effluent conc. (μg L^{-1})	Removal rate (mg m^{-2} d^{-1})/ Removal (%)	Author/ Country
Full (FWS+FWS) /Secondary	D	Triclosan	0.13/NA	29/6.9	0.5/NA	NA	0.25/48/ 1.0	NA/*Acoru, Typha* spp.	NA/(543 /116)	NA/NA	NA/70	Park et al. [27]/Korea
	D	Tris (2-chloroethyl) phosphate	0.13/NA	29/6.9	0.5/NA	NA	As above	As above	As above	NA/NA	NA/10	
Pilot (H+H+H)/ Secondary	D	Tonalide	NA/13	21/7.1	0.04/ NA	9.3	3.5/26/0.8	30 cm Gravel (5)/*Phragmites australis*	5.4/(-97/ +126)	1.8/0.04	0.1/98	Ávila et al. [28]/Spain
Lab (FWS+H) /Secondary	D	Methyl dihydro-jasmonate	0.5/18	16/7.3	0.05/ CF	5.0	5.1/17/3	25 cm Siliceous gravel (4.0)/ *Typha angustifolia*	0.4 (0.5/0.3) /+26/(+9/ -55)	4.0/0.7	0.2/82	Hijosa-Valsero et al. [3]/ Spain
	D	Galaxolide	0.5/18	16/7.3	0.05/ CF	5.0	5.1/17/3	As above	As above	1.2/0.3	0.04/75	
	D	Tonalide	0.5/18	16/7.3	0.05/ CF	5.0	5.1/17/3	As above	As above	0.4/0.1	0.01/70	
Lab (FWS+H) /Secondary	D	Methyl dihydro-jasmonate	0.5/18	17/7.9	0.05/ CF	5.0	6.1/17/3	25 cm Siliceous gravel (4.0)/ Unplanted	1.4 (2.4/0.3) /+26/(+2 5/-118)	4.0/0.8	0.2/80	Hijosa-Valsero et al. [3]/ Spain
	D	Galaxolide	0.5/18	17/7.9	0.05/ CF	5.0	6.1/17/3	As above	As above	1.2/0.3	0.04/75	
	D	Tonalide	0.5/18	17/7.9	0.05/ CF	5.0	6.1/17/3	As above	As above	0.4/0.1	0.01/75	
Lab (FWS+H) /Secondary	D	Methyl dihydro-jasmonate	0.5/6.0	5.2/7.0	0.05/ CF	25	5.1/11/3	25 cm Siliceous gravel (4.0)/ *Typha angustifolia*	0.6 (0.7/0.4) /-6/(+15/ -16)	12/3.0	0.4/75	Hijosa-Valsero et al. [3]/ Spain

Wetland type/Scale/ type of treatment	W T	PCPs	Depth (m)/Area (m² PE⁻¹)	T (°C) /pH	HLR (m³ m⁻² d⁻¹)/ OM	OLR (g COD m⁻² d⁻¹)	HRT (days) /SA/ED (months)	Filter media (mm)/Plants	Effluent DO (mg L⁻¹)/ ORP (Inf/Eff) (mV)	Influent/ Effluent conc. (μg L⁻¹)	Removal rate (mg m⁻² d⁻¹)/ Removal (%)	Author/ Country
	D	Galaxolide	0.5/6.0	5.2/7.0	0.05/ CF	25	5.1/11/3	As above	As above	0.9/0.3	0.03/65	
	D	Tonalide	0.5/6.0	5.2/7.0	0.05/ CF	25	5.1/11/3	As above	As above	0.4/0.2	0.01/60	
Lab (FWS+H) /Secondary	D	Methyl dihydro-jasmonate	0.5/6.0	5.1/7.6	0.05/ CF	25	6.1/11/3	25 cm Siliceous gravel (4.0)/ Unplanted	2.4 (3.5/1.2/ -6/(+40/ +3)	12/4.2	0.4/65	Hijosa-Valsero et al. [3]/ Spain
	D	Galaxolide	0.5/6.0	5.1/7.6	0.05/ CF	25	6.1/11/3	As above	As above	0.9/0.5	0.02/42	
	D	Tonalide	0.5/6.0	5.1/7.6	0.05/ CF	25	6.1/11/3	As above	As above	0.4/0.2	0.01/52	
Full (FP+FWS+H)/ Secondary	D	Methyl dihydro-jasmonate	1.6 FP/ 0.3 FWS/ 0.5 H/5.8	NA/ NA	0.02/ CF	4.0	75.9+1.2+ 5.7/NA/2	[30 cm Gravel (6-8)]FWS/ *Typha latifolia* [55 cm Gravel (6-8)]H /*Salix atrocinerea*	(9.0 FP/ 2.0 FWS/ 1.0 H)/ NA	12/1.2	0.2/90	Hijosa-Valsero et al. [29] /Spain
Full (FP+FWS+H)/ Secondary	D	Methyl dihydro-jasmonate	1.8 FP/ NA FWS/ NA H/1.2	NA/ NA	0.24/ NA	33	4.2+3.5+ 3.2/NA/2	[NA]FWS/ *Typha latifolia* [NA]H/*Salix atrocinerea*	(3.0 FP/ 3.0 FWS/ 6.0 H)/ NA	6.6/0.3	1.5/95	Hijosa-Valsero et al. [29] /Spain
Pilot (H+H+H)/ Secondary	D	Galaxolide	0.45-0.8/ 12	12/NA	0.04/IF	9.3	3.5/24/0.2	30 cm Siliceous gravel (4.0)/ *Phragmites australis*	3.5/(+12 4/-68)	1.1/1.9	NR	Hijosa-Valsero et al. [30] /Spain
	D	Tonalide	As above	12/NA	0.04/IF	9.3	3.5/24/0.2	As above	As above	0.3/0.6	NR	

Wetland type/Scale/ type of treatment	WT	PCPs	Depth (m)/Area (m² PE⁻¹)	T (°C) /pH	HLR (m³ m⁻² d⁻¹)/ OM	OLR (g COD m⁻² d⁻¹)	HRT (days) /SA/ED (months)	Filter media (mm)/Plants	Effluent DO (mg L⁻¹)/ ORP (Inf/Eff) (mV)	Influent/ Effluent conc. (μg L⁻¹)	Removal rate (mg m⁻² d⁻¹)/ Removal (%)	Author/ Country
	D	Methyl dihydro-jasmonate	As above	12/NA	0.04/IF	9.3	3.5/24/0.2	As above	As above	16/5.7	0.4/64	
Pilot (H+H+H)/ Secondary	D	Galaxolide	0.45-0.8/ 12	22/NA	0.04/IF	6.8	3.5/24/0.2	30 cm Siliceous gravel (4.0)/ *Phragmites australis*	0.4/(+124/+116)	1.3/0.2	0.04/85	Hijosa-Valsero et al. [30] /Spain
	D	Tonalide	As above	22/NA	0.04/IF	6.8	3.5/24/0.2	As above	As above	0.5/0.1	0.01/77	
	D	Methyl dihydro-jasmonate	As above	22/NA	0.04/IF	6.8	3.5/24/0.2	As above	As above	21/0.4	0.7/98	
Pilot (H+H+H)/ Secondary	D	Galaxolide	0.45-0.8/ 12	11/NA	0.04/ CF	9.3	3.5/24/0.2	30 cm Siliceous gravel (4.0)/ *Phragmites australis*	3.5/(+124/-106)	1.1/2.7	NR	Hijosa-Valsero et al. [30] /Spain
	D	Tonalide	As above	11/NA	0.04/ CF	9.3	3.5/24/0.2	As above	As above	0.3/0.9	NR	
	D	Methyl dihydro-jasmonate	As above	11/NA	0.04/ CF	9.3	3.5/24/0.2	As above	As above	16/10	0.2/35	
Pilot (H+H+H)/ Secondary	D	Galaxolide	0.45-0.8/ 12	21/NA	0.04/ CF	6.8	3.5/24/0.2	30 cm Siliceous gravel (4.0)/ *Phragmites australis*	0.8/(+124/+92)	1.3/0.2	0.04/85	Hijosa-Valsero et al. [30] /Spain
	D	Tonalide	As above	21/NA	0.04/ CF	6.8	3.5/24/0.2	As above	As above	0.5/0.1	0.01/74	

Wetland type/Scale/ type of treatment	WT	PCPs	Depth (m)/Area (m^2 PE^{-1})	T (°C) /pH	HLR (m^3 m^{-2} d^{-1})/ OM	OLR (g COD m^{-2} d^{-1})	HRT (days) /SA/ED (months)	Filter media (mm)/Plants	Effluent DO (mg L^{-1})/ ORP (Inf/Eff) (mV)	Influent/ Effluent conc. (µg L^{-1})	Removal rate (mg m^{-2} d^{-1})/ Removal (%)	Author/ Country
	D	Methyl dihydro-jasmonate	As above	21/NA	0.04/ CF	6.8	3.5/24/0.2	As above	As above	21/0.4	0.7/98	
Pilot (H+H+H)/ Secondary	D	Galaxolide	0.45-0.8/ 8.1	5.7/NA	0.04/ CF	18.6	3.5/24/0.2	30 cm Siliceous gravel (4.0)/ *Phragmites australis*	0.4/(-6.0 /-36)	0.9/1.1	NR	Hijosa-Valsero et al. [30] /Spain
	D	Tonalide	As above	5.7/NA	0.04/ CF	18.6	3.5/24/0.2	As above	As above	0.4/0.4	NR	
	D	Methyl dihydro-jasmonate	As above	5.7/NA	0.04/ CF	18.6	3.5/24/0.2	As above	As above	12/7.1	0.2/38	
Pilot (H+H+H)/ Secondary	D	Galaxolide	0.45-0.8/ 24	17/NA	0.04/ CF	3.8	3.5/24/0.2	30 cm Siliceous gravel (4.0)/ *Phragmites australis*	0.3/(-6.0 /-102)	1.2/0.9	0.01/29	Hijosa-Valsero et al. [30] /Spain
	D	Tonalide	As above	17/NA	0.04/ CF	3.8	3.5/24/0.2	As above	As above	0.4/0.2	0.005/38	
	D	Methyl dihydro-jasmonate	As above	17/NA	0.04/ CF	3.8	3.5/24/0.2	As above	As above	4.0/0.1	0.1/96	
Pilot (H+H+H)/ Secondary	D	Galaxolide	0.45-0.8/ 8.1	4.5/NA	0.04/ CF	18.6	3.5/24/0.2	30 cm Siliceous gravel (4.0)/ Unplanted	0.6/(-6.0 /-47)	0.9/1.3	NR	Hijosa-Valsero et al. [30] /Spain
	D	Tonalide	As above	4.5/NA	0.04/ CF	18.6	3.5/24/0.2	As above	As above	0.4/0.4	NR	
	D	Methyl dihydro-jasmonate	As above	4.5/NA	0.04/ CF	18.6	3.5/24/0.2	As above	As above	12/7.8	0.1/32	

Wetland type/Scale/ type of treatment	WT	PCPs	Depth (m)/Area (m^2 PE^{-1})	T (°C) /pH	HLR (m^3 m^{-2} d^{-1})/ OM	OLR (g COD m^{-2} d^{-1})	HRT (days) /SA/ED (months)	Filter media (mm)/Plants	Effluent DO (mg L^{-1})/ ORP (Inf/Eff) (mV)	Influent/ Effluent conc. (µg L^{-1})	Removal rate (mg m^{-2} d^{-1})/ Removal (%)	Author/ Country
Pilot (H+H+H)/ Secondary	D	Galaxolide	0.45-0.8/ 24	17/NA	0.04/ CF	3.8	3.5/24/0.2	30 cm Siliceous gravel (4.0)/ Unplanted	0.2/(-6.0 /-64)	1.2/1.1	0.003/6.6	Hijosa-Valsero et al. [30] /Spain
	D	Tonalide	As above	17/NA	0.04/ CF	3.8	3.5/24/0.2	As above	As above	0.4/0.3	0.001/8.1	
	D	Methyl dihydro-jasmonate	As above	17/NA	0.04/ CF	3.8	3.5/24/0.2	As above	As above	4.0/0.6	0.1/86	
Full (P+P+FWS)/ Tertiary	D	Cashmeran	0.5/0.4	16/NA	0.2/NA	NA	8.5/132/4	NA/ *Phragmites australis, Typha latifolia*	3.3/NA	0.2/0.03	0.02/80	Matamoros and Salvadó [31]/Spain
	D	Celestolide	0.5/0.4	16/NA	0.2/NA	NA	8.5/132/4	As above	3.3/NA	0.03/0.01	0.002/54	
	D	Galaxolide	0.5/0.4	16/NA	0.2/NA	NA	8.5/132/4	As above	3.3/NA	0.8/0.04	0.1/95	
	D	Tonalide	0.5/0.4	16/NA	0.2/NA	NA	8.5/132/4	As above	3.3/NA	0.3/0.04	0.04/88	
	D	Methyl dihydro-jasmonate	0.5/0.4	16/NA	0.2/NA	NA	8.5/132/4	As above	3.3/NA	0.5/0.2	0.05/64	
	D	Triclosan	0.5/0.4	16/NA	0.2/NA	NA	8.5/132/4	As above	3.3/NA	0.05/0.01	0.01/86	
	D	Oxybenzone	0.5/0.4	16/NA	0.2/NA	NA	8.5/132/4	As above	3.3/NA	0.1/0.03	0.01/77	
	D	Tris (2-chloroethyl) phosphate	0.5/0.4	16/NA	0.2/NA	NA	8.5/132/4	As above	3.3/NA	0.22/0.16	0.01/27	
	D	Tributyl phosphate	0.5/0.4	16/NA	0.2/NA	NA	8.5/132/4	As above	3.3/NA	0.06/0.02	0.01/60	
Lab (FWS+H) /Secondary	D	Methyl dihydro-jasmonate	0.5/18	16/7.4	0.05/ CF	6.0	1/36/9	[25 cm Siliceous gravel (4.0)] H /*Typha angustifolia*	1.0 (1.4/0.6) /+2.7/ (+60/ -15)	5.8/0.6	0.3/89	Reyes-Contreras et al. [4] /Spain

Wetland type/Scale/ type of treatment	WT	PCPs	Depth (m)/Area (m² PE⁻¹)	T (°C) /pH	HLR (m³ m⁻² d⁻¹)/ OM	OLR (g COD m⁻² d⁻¹)	HRT (days) /SA/ED (months)	Filter media (mm)/Plants	Effluent DO (mg L⁻¹)/ ORP (Inf/Eff) (mV)	Influent/ Effluent conc. (µg L⁻¹)	Removal rate (mg m⁻² d⁻¹)/ Removal (%)	Author/ Country
	D	Galaxolide	0.5/18	16/7.4	0.05/ CF	6.0	1/36/9	As above	As above	2.1/0.5	0.08/76	
	D	Tonalide	0.5/18	16/7.4	0.05/ CF	6.0	1/36/9	As above	As above	0.4/0.1	0.01/67	
Lab (FWS+H) /Secondary	D	Methyl dihydro-jasmonate	0.5/8.4	5.2/7.0	0.05/ CF	17	1/30/6	[25 cm Siliceous gravel (4.0)] H /*Typha angustifolia*	0.8 (1.1/0.5) /-41/ (+15/ -74)	6.6/1.8	0.2/72	Reyes-Contreras et al. [4] /Spain
	D	Galaxolide	0.5/8.4	5.2/7.0	0.05/ CF	17	1/30/6	As above	As above	1.5/0.5	0.05/68	
	D	Tonalide	0.5/8.4	5.2/7.0	0.05/ CF	17	1/30/6	As above	As above	0.3/0.1	0.01/66	
Lab (FWS+H) /Secondary	D	Methyl dihydro-jasmonate	0.5/18	18/7.9	0.05/ CF	6.0	0.6/36/9	[25 cm Siliceous gravel (4.0)] H /Unplanted	2.6 (4.6/0.5) /+2.7/ (+91/- 79)	5.8/1.0	0.2/82	Reyes-Contreras et al. [4] /Spain
	D	Galaxolide	0.5/18	18/7.9	0.05/ CF	6.0	0.6/36/9	As above	As above	2.1/0.7	0.07/69	
	D	Tonalide	0.5/18	18/7.9	0.05/ CF	6.0	0.6/36/9	As above	As above	0.4/0.2	0.01/62	
Lab (FWS+H) /Secondary	D	Methyl dihydro-jasmonate	0.5/8.4	5.1/7.7	0.05/ CF	17	0.6/30/6	[25 cm Siliceous gravel (4.0)] H/ Unplanted	1.7 (2.6/0.9) /-41/ (+36/ -40)	6.6/1.9	0.2/71	Reyes-Contreras et al. [4] /Spain
	D	Galaxolide	0.5/8.4	5.1/7.7	0.05/ CF	17	0.6/30/6	As above	As above	1.5/0.7	0.04/52	
	D	Tonalide	0.5/8.4	5.1/7.7	0.05/ CF	17	0.6/30/6	As above	As above	0.3/0.1	0.01/61	

Wetland type/Scale/ type of treatment	WT	PCPs	Depth (m)/Area (m^2 PE^{-1})	T (°C) /pH	HLR (m^3 m^{-2} d^{-1})/ OM	OLR (g COD m^{-2} d^{-1})	HRT (days) /SA/ED (months)	Filter media (mm)/Plants	Effluent DO (mg L^{-1})/ ORP (Inf/Eff) (mV)	Influent/ Effluent conc. (µg L^{-1})	Removal rate (mg m^{-2} d^{-1})/ Removal (%)	Author/ Country
Pilot (H+H+H)/ Secondary	D	Tonalide	0.45-0.5/10	14/6.9	0.04/ CF	12	3.5/34/0.9	30 cm Gravel (7.3)/ *Phragmites australis*	NA/ (-196/ -96)	3.4/0.4	0.1/88	Ávila et al. [32]/Spain
	D	Oxybenzone	0.45-0.5/10	14/6.9	0.04/ CF	12	3.5/34/0.9	As above	As above	8.3/1.1	0.3/87	
Pilot (H+H+H)/ Secondary	D	Tonalide	0.45-0.5/12	14/6.9	0.04/IF	10	3.5/34/0.9	As above	NA/ (-87/-11)	3.4/0.3	0.1/91	Ávila et al. [32]/Spain
	D	Oxybenzone	0.45-0.5/12	14/6.9	0.04/IF	10	3.5/34/0.9	As above	As above	8.2/0.4	0.3/95	
Full (H+H+FWS)/ Tertiary	D	N,N-diethyl-3-methyl benzoylamide	NA/NA	NA/ NA	0.8/CF	NA	1.6/NA/2	NA/[*Cyperus alternifolius, Thalia dealbata*] H, [*Thalia dealbata, Canna lily, Iris pseudacorus, Herba Saururi Chinensis, Oenanthe javanica*] H, [*Nymphaea alba, Herba Saururi Chinensis, Pontederia cordata, Iris tectorum, Thalia*	NA/NA	0.05/0.07	NR	Zhu and Chen [33]/ China

Wetland type/Scale/ type of treatment	WT	PCPs	Depth (m)/Area (m^{2} PE^{-1})	T (°C) /pH	HLR (m^{3} m^{-2} d^{-1})/ OM	OLR (g COD m^{-2} d^{-1})	HRT (days) /SA/ED (months)	Filter media (mm)/Plants	Effluent DO (mg L^{-1})/ ORP (Inf/Eff) (mV)	Influent/ Effluent conc. (µg L^{-1})	Removal rate (mg m^{-2} d^{-1})/ Removal (%)	Author/ Country
								dealbata, Hydrocotyle verticillata, Phragmites australis]^{FWS}				
	D	Triclocarban	NA/NA	NA/ NA	0.8/CF	NA	1.6/NA/2	As above	NA/NA	0.008/ 0.01	NR	
Pilot $^{(V+V+H+FWS)}$/ Secondary	D	Tonalide	NA/2.7	14/7.8	0.06/IF	37	4/12/1.4	[10 cm Sand (1-2) Gravel (3-8)]^{V} [30 cm Gravel (4-12) Inlet & outlet, Stones (30-50)]^{H} [10 cm Gravel (4-12)]^{FWS}/ *Phragmites australis*	(3.6 ^{V}/ 0.6 ^{H}/ 5.9 ^{FWS})/ (-88/ +171)	NA/NA	NA/95	Ávila et al. [34]/Spain
	D	Oxybenzone	NA/2.7	14/7.8	0.06/IF	37	4/12/1.4	As above	As above	NA/NA	NA/96	
	D	Triclosan	NA/2.7	14/7.8	0.06/IF	37	4/12/1.4	As above	As above	NA/NA	NA/96	
Pilot $^{(V+V+H+FWS)}$/ Secondary	D	Tonalide	NA/0.9	16/8.0	0.13/IF	110	2/12/1.4	As above	(3.2 ^{V}/ 0.4 ^{H}/ 5.2 ^{FWS})/ (-139/ +158)	NA/NA	NA/85	Ávila et al. [34]/Spain
	D	Oxybenzone	NA/0.9	16/8.0	0.13/IF	110	2/12/1.4	As above	As above	NA/NA	NA/92	
	D	Triclosan	NA/0.9	16/8.0	0.13/IF	110	2/12/1.4	As above	As above	NA/NA	NA/92	

Wetland type/Scale/ type of treatment	WT	PCPs	Depth (m)/Area (m² PE⁻¹)	T (°C) /pH	HLR (m^3 m^{-2} d^{-1})/ OM	OLR (g COD m^{-2} d^{-1})	HRT (days) /SA/ED (months)	Filter media (mm)/Plants	Effluent DO (mg L^{-1})/ ORP (Inf/Eff) (mV)	Influent/ Effluent conc. (µg L^{-1})	Removal rate (mg m^{-2} d^{-1})/ Removal (%)	Author/ Country
Pilot $^{(V+V+H+FWS)}$/ Secondary	D	Tonalide	NA/0.6	18/7.6	0.18/IF	159	1.5/12/1.4	As above	(2.7 ^{V}/ 0.3 ^{H}/ 3.7 ^{FWS})/ (-168/ +156)	NA/NA	NA/85	Ávila et al. [34]/Spain
	D	Oxybenzone	NA/0.6	18/7.6	0.18/IF	159	1.5/12/1.4	As above	As above	NA/NA	NA/95	
	D	Triclosan	NA/0.6	18/7.6	0.18/IF	159	1.5/12/1.4	As above	As above	NA/NA	NA/85	
Full $^{(V+H+FWS)}$/ Secondary	D	Tonalide	0.8 ^{V}/ 0.4 ^{H}/ 0.3 ^{FWS}/11	20/7.8	0.04/IF	11	7.4/54/ NA	[5 cm Sand (1-2) 60 cm Siliceous gravel (4-12) 15 cm Siliceous gravel (25-40)]^{V}/ *Phragmites australis* [40 cm Siliceous gravel (4-12) Inlet & outlet, Stones (40-80)]^{H}/ *Phragmites australis* [20 cm Siliceous gravel)]^{FWS}/	(2.0 ^{V}/ 4.2 ^{H}/ 2.7 ^{FWS})/ (+2/ +129)	0.3/0.02	0.01/94	Ávila et al. [35]/Spain

Wetland type/Scale/ type of treatment	WT	PCPs	Depth (m)/Area (m^2 PE^{-1})	T (°C) /pH	HLR (m^3 m^{-2} d^{-1})/ OM	OLR (g COD m^{-2} d^{-1})	HRT (days) /SA/ED (months)	Filter media (mm)/Plants	Effluent DO (mg L^{-1})/ ORP (Inf/Eff) (mV)	Influent/ Effluent conc. (µg L^{-1})	Removal rate (mg m^{-2} d^{-1})/ Removal (%)	Author/ Country
								Typha spp., *Scirpus* spp., *Iris pseudacorus*, *Carex flacca*, *Cyperus rutundus* and *Juncus* spp.				
	D	Triclosan	As above /11	20/7.8	0.04/IF	11	7.4/54/ NA	As above	As above	0.1/0.03	0.004/77	
Full [(H+H)]/ Secondary	D	Methyl dihydro-jasmonate	0.5/3.6	21/NA	0.1/IF	33	5/120/1	NA/ *Phragmites australis*	0.9/NA	NA/NA	NA/93	Matamoros et al. [36]/ Spain
	D	Galaxolide	0.5/3.6	21/NA	0.1/IF	33	5/120/1	As above	0.9/NA	NA/NA	NA/75	
	D	Oxybenzone	0.5/3.6	21/NA	0.1/IF	33	5/120/1	As above	0.9/NA	NA/NA	NA/78	
	D	Tonalide	0.5/3.6	21/NA	0.1/IF	33	5/120/1	As above	0.9/NA	NA/NA	NA/73	
	D	Methylparaben	0.5/3.6	21/NA	0.1/IF	33	5/120/1	As above	0.9/NA	NA/NA	NA/56	
	D	Tributyl phosphate	0.5/3.6	21/NA	0.1/IF	33	5/120/1	As above	0.9/NA	NA/NA	NA/37	
	D	Triclosan	0.5/3.6	21/NA	0.1/IF	33	5/120/1	As above	0.9/NA	NA/NA	NA/35	
	D	Cashmeran	0.5/3.6	21/NA	0.1/IF	33	5/120/1	As above	0.9/NA	NA/NA	NA/39	
	D	Triphenyl phosphate	0.5/3.6	21/NA	0.1/IF	33	5/120/1	As above	0.9/NA	NA/NA	NA/67	
	D	Tris (2-chloroethyl) phosphate	0.5/3.6	21/NA	0.1/IF	33	5/120/1	As above	0.9/NA	NA/NA	NA/24	
Full [(H+H)]/ Secondary	D	Methyl dihydro-jasmonate	0.5/4.8	10/NA	0.1/IF	24	5/120/1	As above	1.2/NA	NA/NA	NA/79	Matamoros et al. [36]/ Spain
	D	Galaxolide	0.5/4.8	10/NA	0.1/IF	24	5/120/1	As above	1.2/NA	NA/NA	NA/79	

Wetland type/Scale/ type of treatment	WT	PCPs	Depth (m)/Area (m² PE⁻¹)	T (°C) /pH	HLR (m³ m⁻² d⁻¹)/ OM	OLR (g COD m⁻² d⁻¹)	HRT (days) /SA/ED (months)	Filter media (mm)/Plants	Effluent DO (mg L⁻¹)/ ORP (Inf/Eff) (mV)	Influent/ Effluent conc. (µg L⁻¹)	Removal rate (mg m⁻² d⁻¹)/ Removal (%)	Author/ Country
	D	Oxybenzone	0.5/4.8	10/NA	0.1/IF	24	5/120/1	As above	1.2/NA	NA/NA	NA/87	
	D	Tonalide	0.5/4.8	10/NA	0.1/IF	24	5/120/1	As above	1.2/NA	NA/NA	NA/83	
	D	Methylparaben	0.5/4.8	10/NA	0.1/IF	24	5/120/1	As above	1.2/NA	NA/NA	NA/33	
	D	Tributyl phosphate	0.5/4.8	10/NA	0.1/IF	24	5/120/1	As above	1.2/NA	NA/NA	NA/32	
	D	Triclosan	0.5/4.8	10/NA	0.1/IF	24	5/120/1	As above	1.2/NA	NA/NA	NA/45	
	D	Cashmeran	0.5/4.8	10/NA	0.1/IF	24	5/120/1	As above	1.2/NA	NA/NA	NA/50	
	D	Triphenyl phosphate	0.5/4.8	10/NA	0.1/IF	24	5/120/1	As above	1.2/NA	NA/NA	NA/38	
	D	Tris (2-chloroethyl) phosphate	0.5/4.8	10/NA	0.1/IF	24	5/120/1	As above	1.2/NA	NA/NA	NA/7.0	
Lab $^{(FWS+H)}$ /Secondary	D	Methyl dihydro-jasmonate	0.5/20	19/6.7	0.05/ CF	6.0	1.6/36/3	[25 cm Siliceous gravel (4.0)]^{H}/ *Typha angustifolia*	(0.2 ^{FWS}/ 0.2 ^{H})/ (-100/ -134)	7.3/0.9	0.3/87	Hijosa-Valsero et al. [5]/ Spain
	D	Galaxolide	0.5/20	19/6.7	0.05/ CF	6.0	1.6/36/3	As above	As above	3.8/1.2	0.1/68	
	D	Tonalide	0.5/20	19/6.7	0.05/ CF	6.0	1.6/36/3	As above	As above	0.3/0.1	0.01/64	
Lab $^{(FWS+H)}$ /Secondary	D	Methyl dihydro-jasmonate	0.5/20	18/7.4	0.05/ CF	6.0	2.3/36/3	[25 cm Siliceous gravel (4.0)]^{H}/ Unplanted	(0.3 ^{FWS}/ 0.1 ^{H})/ (-100/ -132)	7.3/1.0	0.3/86	Hijosa-Valsero et al. [5]/ Spain
	D	Galaxolide	0.5/20	18/7.4	0.05/ CF	6.0	2.3/36/3	As above	As above	3.8/1.4	0.1/64	
	D	Tonalide	0.5/20	18/7.4	0.05/ CF	6.0	2.3/36/3	As above	As above	0.3/0.1	0.01/64	
Full $^{(V+H)}$/ Tertiary**	D	Triclosan	0.7 ^{V}/ 0.8 ^{H}/17	25.8/ 6.7	0.5/IF	7.2	1/9/2	[Gravel (10-30) Gravel	3.1/NA	0.2/0.03	0.1/84	Dai et al. [37]/China

Wetland type/Scale/ type of treatment	WT	PCPs	Depth (m)/Area (m² PE⁻¹)	T (°C) /pH	HLR (m³ m⁻² d⁻¹)/ OM	OLR (g COD m⁻² d⁻¹)	HRT (days) /SA/ED (months)	Filter media (mm)/Plants	Effluent DO (mg L⁻¹)/ ORP (Inf/Eff) (mV)	Influent/ Effluent conc. (µg L⁻¹)	Removal rate (mg m⁻² d⁻¹)/ Removal (%)	Author/ Country
								(30-50)]^V+H^/ [*Canna glauca, Thalia dealbata, Canna indica, Typha angustifolia*] ^V^ [*Cyperus alternifolius, Arundo donax, Acorus tatarinowii, Desmodium styracifolium*]^H^				
Full ^(V+H)^/ Tertiary**	D	Triclosan	0.7 ^V^/ 0.8 ^H^/8.4	25.8/ 6.7	1.0/IF	14	0.5/9/2	As above	3.1/NA	0.2/0.03	0.2/84	Dai et al. [37]/China
Full ^(V+H)^/ Tertiary**	D	Triclosan	0.7 ^V^/ 0.8 ^H^/5.6	25.8/ 6.7	1.5/IF	21	0.3/9/2	As above	3.1/NA	0.2/0.03	0.2/84	Dai et al. [37]/China
Full ^(V+H)^/ Tertiary**	D	Triclosan	0.7 ^V^/ 0.8 ^H^/4.2	25.8/ 6.7	2.0/IF	29	0.25/9/2	As above	3.1/NA	0.2/0.03	0.3/84	Dai et al. [37]/China
Pilot ^(V+V)^/ Secondary	D	Acesulfame	(0.85 ^VG^ /0.85 ^VS^) /4.1	21/NA	0.05/IF	29	NA/60/12	Gravel (4-8)^VG^ Sand (1-3)^VS^/ *Phragmites australis*	10/ (-277/ +216)	19/10	0.4/47	Kahl et al. [14]/ Germany
Full ^(V+V+H+FWS)^/ Secondary	H	Triclosan	NA/NA	NA/7.6	0.02/ NA	2.1	10/12/12	[60 cm Fluviatile sand] ^V^ [25 cm Fluviatile sand] ^H^/ *Phragmites australis,*	NA/NA	0.2/0.1	0.002/50	Vystavna et al. [38]/ Ukraine

Wetland type/Scale/ type of treatment	W T	PCPs	Depth (m)/Area (m^2 PE^{-1})	T (°C) /pH	HLR (m^3 m^{-2} d^{-1})/ OM	OLR (g COD m^{-2} d^{-1})	HRT (days) /SA/ED (months)	Filter media (mm)/Plants	Effluent DO (mg L^{-1})/ ORP (Inf/Eff) (mV)	Influent/ Effluent conc. (µg L^{-1})	Removal rate (mg m^{-2} d^{-1})/ Removal (%)	Author/ Country
								Typha latifolia L., Scirpus sylvaticus L.				
Full (V+V+H+FWS)/ Secondary	H	Triclosan	NA/NA	NA/7.7	0.03/ NA	4.4	13/48/12	As above	NA/NA	0.3/0.01	0.008/97	Vystavna et al. [38]/ Ukraine
Pilot (V+H+FWS)/ Secondary	D	N,N-diethyl-meta-toluamide	0.8^{V}/ 0.3^{H}/ 0.5^{FWS}/ 1.6	20/7.4	0.1/IF	40	NA/76/4	[10 cm Sand (1-2) 70 cm Gravel (3-8)]^{V} [30 cm Gravel (4-12) Inlet and outlet: Stone (30-50)]^{H}/ *Phragmites australis*	(2.1^{V}/ 2.2^{H}/ 0.8^{FWS})/ (+33/ +69)	2.4/0.5	0.3/80	Sgroi et al. [24]/Spain
	D	Sucralose	As above/ 1.6	20/7.4	0.1/IF	40	NA/76/4	As above	As above	13.1/13.3	NR	
Full (FWS+V+FWS+ FWS+SL)/ Primary	*	N,N-diethyl-3-methyl benzamide	NA/ 6.5	NA/8.0	0.22/ CF	26	1.5/NA/ 12	NA/ *Myriophyllum verticillatum* L., *Pontederia cordata*	NA/NA	0.5/0.01	0.1/97	Chen et al. [39]/China
	*	Triclosan	NA/ 6.5	NA/8.0	0.22/ CF	26	1.5/NA/ 12	As above		0.2/0.04	0.03/81	
	*	Carbendazim	NA/ 6.5	NA/8.0	0.22/ CF	26	1.5/NA/ 12	As above		0.1/0.003	0.02/98	
	*	Methylparaben	NA/ 6.5	NA/8.0	0.22/ CF	26	1.5/NA/ 12	As above		0.06/0.01	0.01/77	

Wetland type/Scale/ type of treatment	WT	PCPs	Depth (m)/Area (m^2 PE^{-1})	T (°C) /pH	HLR (m^3 m^{-2} d^{-1})/ OM	OLR (g COD m^{-2} d^{-1})	HRT (days) /SA/ED (months)	Filter media (mm)/Plants	Effluent DO (mg L^{-1})/ ORP (Inf/Eff) (mV)	Influent/ Effluent conc. (μg L^{-1})	Removal rate (mg m^{-2} d^{-1})/ Removal (%)	Author/ Country
Pilot $^{(V+V)}$/ Secondary	D	Acesulfame	(0.85 ^{VG}/ 0.85 ^{VS})/ 4.0	12/6.9	0.05/IF	30	NA/60/12	Gravel (4-8)^{VG} Sand (1-3)^{VS}/ *Phragmites australis*	9.0/ (-270/ +207)	19/8.7	0.5/54	Nivala et al. [19]/ Germany

Note: Free water surface flow constructed wetland (FWSCW); Horizontal flow constructed wetland (HFCW); Vertical flow constructed wetland (VFCW); Hybrid constructed wetland (HCW); Wastewater type (WT); Domestic (D); Healthcare facility/hospital (H); Personal care products (PCPs); Temperature (T); Hydraulic loading rate (HLR); Operational mode (OM); Intermittently fed (IF); Continuously fed (CF); Organic loading rate (OLR); Chemical oxygen demand (COD); Hydraulic retention time (HRT); System age (SA); Experiment duration (ED); Dissolved oxygen (DO); Oxidation-reduction potential (ORP); Not available (NA); Not removed (NR).

Domestic (70%) and livestock sewage (30%) (*); Two FWSCWs are connected in series ($^{FWS+FWS}$); Two HFCWs are connected in parallel with one HFCW in series ($^{H+H+H}$); FWSCWs on top of horizontal flow filter ($^{FWS+H}$); Two HFCWs and one FWSCW are connected in series ($^{H+H+FWS}$); Two polishing ponds and one FWSCW are connected in parallel ($^{P+P+FWS}$); Facultative pond, FWSCW and HFCW are connected in series ($^{FP+FWS+H}$); Two VFCWs are connected in parallel, and HFCW and FWSCW are connected in series ($^{V+V+H+FWS}$); VFCW, HFCW and FWSCW are connected in series ($^{V+H+FWS}$); Two HFCWs are connected in parallel ($^{H+H}$); VFCW and HFCW are in a stack design ($^{V+H}$)**; Three FWSCWs, one VFCW and one stabilization lagoon are connected in series ($^{FWS+V+FWS+FWS+SL}$); Two VFCWs are connected in parallel ($^{V+V}$).

The Population equivalent (PE) is calculated based on the common relation 1 PE = 60 g BOD d^{-1}. BOD values were approximated using the ratio COD/BOD = 2 in the studies where BOD was not reported [36,37], and COD values were approximated using the ratio COD = 2BOD in the studies where COD was not reported [5,14,19,29].

Supplementary materials 2: Contribution of removal mechanisms of PCPs in experimental studies and CWs.

Table S5. Contribution of removal mechanisms/pathways of PCPs in hydroponic microcosms, media adsorption experiments, and CWs.

PCPs	Removal mechanism	Contribution in removal (%) from different mechanisms	References
Triclosan	Biodegradation	84	Li et al. [7]
	Photodegradation	58	Li et al. [7]
	Photodegradation	80	Matamoros et al. [40]
	Sorption	19	Matamoros et al. [40]
	Plant uptake	11	Li et al. [7]
Methyl dihydrojasmonate	Plant uptake	67±26	Hijosa-Valsero et al. [3]
	Plant uptake	71±24	Reyes-Contreras et al. [4]
	Plant uptake	85±8	Hijosa-Valsero et al. [5]
Galaxolide	Plant uptake	51±34	Hijosa-Valsero et al. [3]
	Plant uptake	59±30	Reyes-Contreras et al. [4]
	Plant uptake	85±6	Hijosa-Valsero et al. [5]
Tonalide	Plant uptake	45±36	Hijosa-Valsero et al. [3]
	Plant uptake	53±25	Reyes-Contreras et al. [4]
	Plant uptake	80±6	Hijosa-Valsero et al. [5]
N,N-diethyl-meta-toluamide	Biodegradation	4.5	Li et al. [7]
	Photodegradation	1.2	Li et al. [7]
	Plant uptake	9.1	Li et al. [7]

Supplementary materials 3: The estimated statistics (mean and standard deviation of concentration and removal) for personal care products for which three or more data points were available.

Table S6. Mean and standard deviation of 15 widely studied personal care products.

**Class/PCPs**	**No. of observation based on removal (%)**	**Influent conc. (μg L^{-1}) Mean ± Stdev**	**Effluent conc. (μg L^{-1}) Mean ± Stdev**	**Removal rate (mg m^{-2} d^{-1}) Mean ± Stdev**	**Removal efficiency (%) Mean ± Stdev**
**Artificial sweeteners**					
Acesulfame	6	19±0	16±5	0.1±0.3	17±27
**Preservatives**					
Methylparaben	10	39±73	4.2±7.6	4.3±7.9	46±33
Propylparaben	5	60±82	15±25	5.4±7.6	18±55
**Insect repellents**					
N,N-diethyl-meta-toluamide	4	8±11	7±12	0.1±0.1	52±45
**Antiseptics**					
Triclosan	46	39±110	1.3±3.1	3.8±6.4	72±28
Triclocarban	5	0.1±0.1	0.01±0.0	NA	62±44
**Fragrances**					
Methyl dihydro-jasmonate	76	7.1±4.4	1.9±2.2	0.5±0.4	74±20
Cashmeran	3	0.2	0.03	0.02	56±21
Galaxolide	73	2.2±1.0	0.9±0.6	0.2±0.2	59±26
Tonalide	57	0.6±0.6	0.2±0.2	0.02±0.03	62±25
**Flame retardants**					
Tributyl phosphate	5	0.4±0.3	0.2±0.1	0.004±0.005	45±14
Triphenyl phosphate	4	0.1±0.0	0.02±0.01	0.0002±0.0	66±19
Tris (2-chloroethyl) phosphate	6	0.4±0.2	0.3±0.1	0.004±0.006	19±9
**Sunscreen agents**					
Oxybenzone	14	3.6±4.9	0.3±0.3	0.2±0.3	84±17
Sulisobenzone	3	4.2±0.0	3.8±0.1	1.2±0.3	9.0±1.9

Supplementary materials 4: The selected statistics on risk quotient for nine PCPs based on effluent concentration in CWs.

Table S7. The statistics on risk quotient of nine selected PCPs based on effluent concentration in CWs.

Class/PCPs	N	Mean	STDEV	Min	Max	P10	P25	P50	P75	P90
**Preservatives**										
Methylparaben	8	0.379	0.679	0.001	1.607	0.002	0.002	0.014	0.375	1.420
**Antiseptics**										
Triclosan	40	10.191	23.668	0.008	100.000	0.077	0.231	0.769	6.346	40.846
Triclocarban	5	1.000	0.000	1.000	1.000	1.000	1.000	1.000	1.000	1.000
**Fragrances**										
Methyl dihydro-jasmonate	74	0.122	0.137	0.006	0.633	0.013	0.038	0.060	0.169	0.374
Galaxolide	76	0.260	0.169	0.011	0.771	0.086	0.114	0.229	0.371	0.514
Tonalide	58	0.033	0.027	0.003	0.132	0.015	0.015	0.029	0.044	0.059
**Flame retardants**										
Tributyl phosphate	3	0.030	0.024	0.003	0.052	0.010	0.019	0.034	0.043	0.048
Tris (2-chloroethyl) phosphate	3	0.001	0.001	0.001	0.002	0.001	0.001	0.002	0.002	0.002
**Sunscreen agents**										
Oxybenzone	11	0.143	0.163	0.016	0.579	0.016	0.034	0.105	0.184	0.211

Note: Number of datapoints (N); Standard deviation (STDEV); Minimum (Min); Maximum (Max); Percentile (P); Risk is categorized into four levels: high risk (RQ > 1.0), medium risk (0.1 ≤ RQ ≤ 1.0), low risk (0.01 ≤ RQ ≤ 0.1), and no risk (RQ < 0.01); Statistics were not calculated in the case of cashmeran and triphenyl phosphate due to one and two data point, respectively.

Supplementary materials 5: A synthesis on the role of physicochemical properties of EOCs in their removal mechanisms.

Table S8. Role of physicochemical properties of EOCs in their removal mechanisms.

Physicochemical properties	Definition	References	Values	Nature of the compound	References	Expected removal mechanism
Log Kow	This is the ratio of the concentration of unionized compound between octanol and water and for organic compounds, it is considered as the standard measure of their hydrophobicity. The hydrophobicity of non-ionic organic compounds is invariable under different pH conditions, thus Log Kow is appropriate to measure their hydrophobicity.	Burken and Schnoor [41]; Reinhold et al. [42]	Log Kow > 4.0	Highly hydrophobic	Briggs et al. [43]; Alvarez et al. [44]; Le-Minh et al. [45]; Dan et al. [46]	Adsorption
			1.0 < Log Kow < 3.5	Moderately hydrophobic	Briggs et al. [43]; Dietz and Schnoor [47]; Alvarez et al. [44]; Pilon-Smits [48]; Le-Minh et al. [45]	Plant uptake
			Log Kow > 3.5	Highly hydrophobic	Dietz and Schnoor [47]	Phytostabilization; rhizosphere bioremediation (biodegradation)
			Log Kow < 1.0	Highly hydrophillic	Vystavna et al. [38]	Other than adsorption/ plant uptake
Log Dow	In the case of ionic organic compounds Log Dow is appropriate to represent the hydrophobicity, since the hydrophobicity of ionic organic compounds varies due to	Lee et al. [49]	Log Dow > 2.5	Highly hydrophobic	Briggs et al. [43]; Alvarez et al. [44]; Le-Minh et al. [45]; Dan et al. [46]	Adsorption

Physicochemical properties	Definition	References	Values	Nature of the compound	References	Expected removal mechanism
	ionization of the compound at certain pH.					
			Log Dow < 2.5	Moderately hydrophobic	Briggs et al. [43]; Dietz and Schnoor [47]; Alvarez et al. [44]; Pilon-Smits [48]; Le-Minh et al. [45]	Plant uptake
	Although, the Log Dow of organic compounds is nearly the same as Log Kow but the modification in ionizable functional groups may affect their removal by biological treatment processes.	Zhang et al. [50]				
Log Koc	This is the ratio of the mass of a compound that is adsorbed in the soil per unit mass of organic carbon in the soil.	Piwoni and Keeley [51]	High Log Koc > 2.5	Less mobile organic compounds	Piwoni and Keeley [51]	Sorption onto organic surfaces
			Low Log Koc < 2.5	More mobile organic compounds	Piwoni and Keeley [51]	
Charge	The neutral compounds which are hydrophilic in nature (Log Kow < 1.5) may be taken up by rooted vascular plants via hydrogen bonding with water molecules into the transpiration stream.	Petrie et al. [18]	-	Neutral	Petrie et al. [18]	Plant uptake
	The cationic compounds can partition into lipophilic cell structure of negatively charged biomembranes of the plant roots.	Petrie et al. [18]	-	Cationic	Petrie et al. [18]	Plant uptake

Physicochemical properties	Definition	References	Values	Nature of the compound	References	Expected removal mechanism
	The negatively charged compounds cannot be taken up by the plants because the charge repulsion with the negatively charged biomembrane restricts their uptake by the plant root.	Matamoros et al. [40]; Petrie et al. [18]	-	Anionic	Matamoros et al. [40]; Petrie et al. [18]	Adsorption
Molecular weight	Molecular weight is a measure of the sum of the atomic weight values of the atoms in a molecule.	*	MW > 500 g mol^{-1}	High	Yan et al. [52]	Adsorption
			MW < 500 g mol^{-1}	Moderate	Yan et al. [52]	Plant uptake
			MW < 100 g mol^{-1}	Low	Vystavna et al. [38]	Other than adsorption/ plant uptake
Water solubility	Water solubility is a measure of the amount of chemical substance that can dissolve in water at a specific temperature.	**	WS > 1000 mg L^{-1}	High water solubility	Vystavna et al. [38]	Other than adsorption/ plant uptake

Note: https://www.thoughtco.com/definition-of-molecular-weight-605369 (*); https://www.chemsafetypro.com/Topics/CRA/Water_Solubility.html (**).

Supplementary materials 6: Role of physicochemical properties of PCPs and removal mechanisms in CWs.

Table S9. Role of physicochemical properties of PCPs and removal mechanisms in CWs

Class/ PCPs	Role of physicochemical properties of PCPs and removal mechanisms in CWs
**Artificial sweeteners**	
**Acesulfame**	The removal efficiency of acesulfame was moderate in HCW (51±5%) but very low in HFCW (2.5±3.5%), and it was negatively removed in VFCW (-3.5±2.1%) (Table S10). Its high water solubility(270 g L^{-1} at 25 °C) with low molecular weight (163.15 g mol^{-1}), very low lipophilicity (Log Kow = -1.33; Log Dow = -1.49), and anionic form under neutral conditions (pH = 7) (Table 4), which might hinder its uptake by the plants as well as adsorption to the substrate. Jekel et al. [53] reported that it is persistent to biodegradation in biological treatment. However, some recent studies attributed its removal to biodegradation in biological wastewater treatment [54-56]. This can be explained by its higher removal efficiency in HCW containing two types of substrate media (gravel-based VFCW and subsequent sand-based VFCW). This is due to the reason that sand media provided a larger available surface area for microbial growth and higher oxygen to promote the elimination of substances that are majorly removed by aerobic biodegradation pathways. It was poorly removed by gravel-based HFCW and negatively removed in gravel-based VFCW. However, its moderate removal efficiency was achieved in subsequent sand-based VFCW [14,19]. Its removal by aerobic biodegradation is explicit by significantly higher removal efficiency in aerated (AA)-HFCW (71±12%) and AA-VFCW (54±1%) compared with their corresponding non-aerated (NA)-CWs (2.5±3.5% and -3.5±2.1%, respectively) [14,19] (Table S3). This is moderately biodegradable compound and improvement in dissolved oxygen (DO) level in the aerated CWs might contribute to this enhanced removal.
**Preservatives**	
**Propylparaben**	The removal efficiency of propylparaben was moderate in FWSCW (75±20%) and it was negatively removed in HFCW (-21±21%) (Table S10). It is moderately soluble in water (529.3 mg L^{-1} at 25 °C) and its moderate hydrophobicity and distribution coefficient (Log Kow = 2.98; Log Dow = 2.51) with low molecular weight (180.21 g mol^{-1}), and neutral form under neutral conditions (pH = 7) (Table 4) suggest its removal by plant uptake. Anjos et al. [8] attributed its removal to plant uptake. The removal efficiency by FWSCW, planted with *Landoltia punctata* and *Lemna minor*, was 61% and 89%, respectively (Table S1). In addition to direct uptake, the indirect positive effects of plants presence such as degradation by enzymatic exudates and aerobic biodegradation facilitated by the release of root exudates (such as carbohydrates and amino acids), and oxygen by the plant roots in the rhizosphere [17,37,40,57] might contribute to its removal [40]. These processes might take place in HFCW but its negative removal suggests that photodegradation could contribute to its removal in FWSCW.
**Insect repellents**	

**N,N-diethyl-meta-toluamide**	The removal efficiency of N,N-diethyl-meta-toluamide was higher in HFCW (98%) compared with HCW (80%). It was poorly removed in VFCW (28%), but FWSCW did not show any removal (0.0%) (Table S10). It is moderately soluble in water (912 mg L^{-1} at 25 °C) and its moderate hydrophobicity and distribution coefficient (Log Kow = 2.18; Log Dow = 2.50) with low molecular weight (191.3 g mol^{-1}), and neutral form under neutral conditions (pH = 7) (Table 4) suggest its removal by plant uptake. In spite of this, the lower contribution of plants (9.1%) in hydroponic system (*Spirodela polyrhiza*) compared with the control without plants (17% and 7.9%, respectively) reveals that this removal pathway is contributing very less in its removal (Figure 3 and Table S5). Furthermore, its organic carbon sorption capacity is also low (Log Koc = 1.76) (Table 4). Thus, the efficient removal in CWs might be due to enhancement in biodegradation processes in the presence of plants. However, in the *E-coli* biodegradation experiment the highest removal observed was very low (4.5%) [7] (Figure 3 and Table S5). The highest removal in HFCW followed by HCW indicates that predominantly anaerobic and slightly aerobic conditions might favor its removal [15,24]. In FWSCW, the major process contributes in PCPs removal is photodegradation. This compound is not removed in FWSCWs, which can be explained by its persistence against light. In photodegradation experiment, it has been demonstrated that this compound is not light sensitive (1.2% removal efficiency at highest by photodegradation) [7] (Figure 3 and Table S5).
**Antiseptics**	
**Triclocarban**	The removal efficiency of triclocarban was higher in HFCW (81±13%) but it was negatively removed in HCW (-14%) (Table S10). It is almost insoluble in water (0.11 mg L^{-1} at 25 °C), highly hydrophobic (Log Kow = 4.90; Log Dow = 4.90) with moderate molecular weight (315.6 g mol^{-1}) (Table 4), which suggest its removal by adsorption onto soil particles. Its high organic carbon sorption capacity (Log Koc = 3.73) also favors its removal by sorption onto organic surfaces. This can be seen by its significant positive correlation with the removal of chemical oxygen demand (COD) [16]. Consistent with that, Zhu and Chen [33] estimated its high concentration in sediments reaching an average value of 6.6 mg kg^{-1}, which indicates that sorption to sediments or sludge mainly contributed to its removal.
**Fragrances**	
**Cashmeran**	The removal efficiency of cashmeran was moderate in HCW (56±21%) (Table S10). Its high hydrophobicity (Log Kow = 4.49), very low water solubility (5.94 mg L^{-1} at 25 °C), with moderate molecular weight (206.33 g mol^{-1}) (Table 4) suggest its removal by adsorption onto soil particles. This can be explained by its higher removal efficiency in winter compared with summer (50% and 39%, respectively) [36] (Table S4). Since adsorption is an exothermic process, which is favored by low temperature (in winter). Its high organic carbon sorption capacity (Log Koc = 2.99) also favors its removal by sorption onto organic surfaces. This can be seen by the efficient removal in FWSCW compared with pond of the HCW (combination of two ponds and one FWSCW). The roots of the plants in the CWs increase the accumulation of organic matter as well as the sorption capacity [31].
**Flame retardants**	

**Tributyl phosphate**	The removal efficiency of tributyl phosphate was moderate in HFCW (48±19%) and HCW (43±15%) (Table S10). It is highly hydrophobic (Log Kow = 4.00) with slight water solubility (280 mg L^{-1} at 25 °C), and moderate molecular weight (266.32 g mol^{-1}), which favors its removal by adsorption to substrate. Its high organic carbon sorption capacity (Log Koc = 3.60) (Table 4) suggests its removal by sorption onto organic surfaces. This is explicit by its significant positive correlation with the removal of total suspended solids (TSS) and COD [13]. This can also be seen by its slightly better removal efficiency in summer compared with winter (37% and 32%, respectively) [36] (Table S4) due to the higher activity of the rooted plants in the warm season, since the roots of the plants in the CWs increase the accumulation of organic matter and the sorption capacity [31]. The other indirect positive effect of plants presence such as enhancement in biodegradation might also contribute to its removal, which is revealed by its strong positive relationship with ammonium-nitrogen (NH_4^+-N) removal [36].
**Triphenyl phosphate**	The removal efficiency of triphenyl phosphate was moderate in HFCW (79±5%) and HCW (53±21%) (Table S10). It is almost insoluble in water (1.9 mg L^{-1} at 25 °C), highly hydrophobic (Log Kow = 4.59) with moderate molecular weight (326.29 g mol^{-1}), which suggest its adsorption to substrate as a major removal mechanism. Its high organic carbon sorption capacity (Log Koc = 4.03) (Table 4) reveals its removal by sorption onto organic surfaces, which can be explained by its significant positive correlation with the removal of TSS and COD [13]. It shows a higher removal efficiency in summer compared with winter (67% and 38%, respectively) [36] (Table S4) because of efficient growth of the rooted plants in the warm season also reveals the contribution of this removal pathway. Furthermore, the enhancement in biodegradation in the presence of plants might also contribute to its removal, which is shown by its significant positive correlation with NH_4^+-N removal [36].
**Tris (2-chloroethyl) phosphate**	The removal efficiency of tris (2-chloroethyl) phosphate was very low in HFCW (24±6%) and HCW (17±10%) (Table S10). It is highly water soluble (7.82 g L^{-1} at 25 °C) with moderate hydrophobicity (Log Kow = 1.44), and moderate molecular weight (285.48 g mol^{-1}). It is neutral in nature under neutral conditions (pH = 7) (Table 4), which favors its removal by plant uptake. Its moderate organic carbon sorption capacity (Log Koc = 2.48) also suggests its removal by sorption onto organic surfaces. This is explicit by its significant positive correlation with the removal of TSS and COD [13]. Considering the efficient growth of the rooted plants in summer and the ability of roots to increase the accumulation of organic matter as well as the sorption capacity [31], its higher removal efficiency was observed in summer compared with winter (24% and 7.0%, respectively) [36] (Table S4). However, this compound showed very low removal because of its high recalcitrance [13,58], which might be due to the presence of chlorine in its structure.
**Sunscreen agents**	

**Sulisobenzone**	The removal efficiency of sulisobenzone was very low in HFCW (9.0±1.9%) (Table S10). It is highly water soluble (20.3 g L^{-1} at 25 °C) with moderate molecular weight (308.31 g mol^{-1}), highly hydrophilic (Log Kow = 0.37), and anionic under neutral conditions (pH = 7) (Table 4), which might hinder its uptake by the plants as well as adsorption to the substrate. Its anionic form and very low lipophilicity (Log Dow = -0.53) (Table 4) hinders its partition into lipophilic cell structure of negatively charged biomembranes of the plant roots because of the charge repulsion. Although its molecular weight is moderate, but it is most hydrophilic (Log Kow < 1.0) and the most water soluble (WS > 1000 mg L^{-1}), thus adsorption cannot be considered as a removal mechanism because for the sorption/sedimentation of these types of compounds more time is required [38]. Therefore, this low removal can be attributed to biodegradation, which has been established in other wastewater treatment technologies. For instance, in contact with activated sludge it was degraded in aerobic batch experiments forming at least nine transformation products (TPs) [59]. Beel et al. [59] proposed biodegradation pathway based on the structure of the TPs identified and the sequence of their formation.

Supplementary materials 7: The removal efficiency of 15 widely studied PCPs and the results of one-way ANOVA and z-Test for comparison of means of six selected PCPs.

Table S10. Removal efficiency (mean % and standard deviation) of 15 widely studied PCPs in different types of CWs.

Class/PCPs	FWSCW (n)	HFCW (n)	VFCW (n)	HCW (n)
**Artificial sweeteners**				
Acesulfame	NA	2.5±3.5 (2)	-3.5±2.1 (2)	51±5 (2)
**Preservatives**				
Methylparaben	90±1 (2)	23±21 (5)	NA	55±22 (3)
Propylparaben	75±20 (2)	- 21±21 (3)	NA	NA
**Insect repellents**				
N,N-diethyl-meta-toluamide	0.0 (1)	98 (1)	28 (1)	80 (1)
**Antiseptics**				
Triclosan	97±2 (4)	59±31 (18)	88±9 (8)	77±19 (15)
Triclocarban	NA	81±13 (4)	NA	- 14 (1)
**Fragrances**				
Methyl dihydrojasmonate	71±20 (15)	73±21 (37)	99 (1)	76±19 (23)
Cashmeran	NA	NA	NA	56±21 (3)
Galaxolide	63±26 (18)	53±26 (37)	90 (1)	65±22 (17)
Tonalide	59±27 (18)	43±25 (12)	74±11 (3)	72±20 (24)
**Flame retardants**				
Tributyl phosphate	NA	48±19 (2)	NA	43±15 (3)
Triphenyl phosphate	NA	79±5 (2)	NA	53±21 (2)
Tris (2-chloroethyl) phosphate	NA	24±6 (2)	NA	17±10 (4)
**Sunscreen agents**				
Oxybenzone	NA	64±28 (3)	94±4 (3)	88±8 (8)
Sulisobenzone	NA	9.0±1.9 (3)	NA	NA

Note: Number of data points (n); Not available (NA)

Table S11. The results (p-values) of one-way ANOVA and z-Test for comparison of means for six selected PCPs with different types of CWs.

Parameter	Galaxolide	Methyl dihydro-jasmonate	Tonalide	Triclosan	Methylparaben	Oxybenzone
			**ANOVA results**			
	0.21	0.70	**0.01**	**0.01**	0.08	**0.03**
			**z-Test results**			
FWSCW Vs HFCW	0.22	0.75	0.10	**0.01**	NA	NA
FWSCW Vs VFCW	NA	NA	0.11	**0.01**	NA	NA
FWSCW Vs HCW	0.75	0.41	0.1	**0.01**	NA	NA
HFCW Vs VFCW	NA	NA	**0.002**	**0.0003**	NA	0.06
HFCW Vs HCW	0.09	0.52	**0.001**	0.05	**0.04**	0.13
VFCW Vs HCW	NA	NA	0.78	0.05	NA	0.13

Note: Bold values indicate significant difference at $\alpha = 0.05$ ($p < 0.05$) for ANOVA and z-test results; Not available (NA).

Supplementary materials 8: The estimated statistics (mean and standard deviation of removal efficiency) of widely studied 15 PCPs in primary, secondary, and tertiary treatment by CWs.

Table S12. Statistics (mean and standard deviation) of widely studied 15 PCPs in primary, secondary, and tertiary treatment by CWs.

Class/PCPs	Primary<br>Removal efficiency (%)<br>Mean ± Stdev (n)	Secondary<br>Removal efficiency (%)<br>Mean ± Stdev (n)	Tertiary<br>Removal efficiency (%)<br>Mean ± Stdev (n)
**Artificial sweeteners**			
Acesulfame	NA	27±28 (4)	NA
**Preservatives**			
Methylparaben	77 (1)	67±28 (4)	23±21 (5)
Propylparaben	NA	75±20 (2)	19±17 (3)
**Insect repellents**			
N,N-diethyl-meta-toluamide	0.0 (1)	54±37 (2)	98 (1)
**Antiseptics**			
Triclosan	85±15 (17)	68±31 (16)	65±27 (12)
Triclocarban	79±15 (3)	NA	86 (1)
**Fragrances**			
Methyl dihydro-jasmonate	76±18 (24)	74±22 (49)	60±4 (3)
Cashmeran	NA	45±8 (2)	80 (1)
Galaxolide	61±23 (24)	55±27 (43)	76±21 (6)
Tonalide	NA	60±25 (51)	75±22 (6)
**Flame retardants**			
Tributyl phosphate	NA	35±4 (2)	52±15 (3)
Triphenyl phosphate	NA	53±21 (2)	79±5 (2)
Tris (2-chloroethyl) phosphate	NA	14±10 (3)	25±5 (3)
**Sunscreen agents**			
Oxybenzone	NA	91±6 (10)	67±24 (4)
Sulisobenzone	NA	NA	9±2 (3)

Note: Number of data points (n); Not available (NA).

Supplementary materials 9: Effect of artificial aeration on the removal of PCPs in CWs.

Table S13. Removal efficiency (mean % and standard deviation) of PCPs in different types of aerated (AA) and non-aerated (NA) CWs.

Parameter/ PCPs	NA-FWSCW/ AA-FWSCW	NA-HFCW/ AA- HFCW	NA-VFCW/ AA-VFCW	Dominant removal mechanism*
DO (mg L^{-1})	6.0/NA	3.0/10	3.8±0.7/6.3±1.5	
Triclosan	94/99	na	73/86	Adsorption; Biodegradation (aerobic); Photodegradation
Tonalide	na	na	61/83	Sorption; Adsorption
Oxybenzone	na	na	89/91	Adsorption **; Biodegradation (aerobic); Sorption
Acesulfame	na	2.5±3.5/71±12	-3.5±2.1/54±1	Biodegradation (aerobic)
N,N-diethyl-meta-toluamide	0.0/4.2	na	na	Biodegradation (anaerobic)**

Note: Data is taken from: Ávila et al. [22]; Li et al. [7]; Kahl et al. [14]; and Nivala et al. [19]. The enhanced removal is explicit in the case of AA-FWSCW (triclosan), AA-HFCW (acesulfame), AA-VFCW (triclosan, tonalide, oxybenzone and acesulfame) compared with their corresponding non-aerated CWs; Not available (na); Authors' own insight based on physicochemical properties, removal mechanisms and limited evidence in the literature (*); Authors' own insight based on physicochemical properties and removal mechanisms (**).

References

1. Matamoros, V.; García, J.; Bayona, J.M. Organic micropollutant removal in a full-scale surface flow constructed wetland fed with secondary effluent. *Water Res.* **2008**, 42 (3), 653-660. DOI: https://doi.org/10.1016/j.watres.2007.08.016
2. Llorens, E.; Matamoros, V.; Domingo, V.; Bayona, J.M.; García, J. Water quality improvement in a full-scale tertiary constructed wetland: effects on conventional and specific organic contaminants. *Sci. Total Environ*. **2009**, 407, 2517-2524. DOI: https://doi.org/10.1016/j.scitotenv.2008.12.042
3. Hijosa-Valsero, M.; Matamoros, V.; Sidrach-Cardona, R.; Martin-Villacorta, J.; Becares, E.; Bayona, J.M. Comprehensive assessment of the design configuration of constructed wetlands for the removal of pharmaceuticals and personal care products from urban wastewaters. *Water Res*. **2010**, 44 (12), 3669-3678. DOI: https://doi.org/10.1016/j.watres.2010.04.022
4. Reyes-Contreras, C.; Hijosa-Valsero, M.; Sidrach-Cardona, R.; Bayona, J.M.; Bécares, E. Temporal evolution in PPCP removal from urban wastewater by constructed wetlands of different configuration: a medium-term study. *Chemosphere* **2012**, 88 (2), 161-167. DOI: https://doi.org/10.1016/j.chemosphere.2012.02.064
5. Hijosa-Valsero, M.; Reyes-Contreras, C.; Domínguez, C.; Bécares, E.; Bayona, J.M. Behaviour of pharmaceuticals and personal care products in constructed wetland compartments: Influent, effluent, pore water, substrate and plant roots. *Chemosphere* **2016**, 145, 508-517. DOI: https://doi.org/10.1016/j.chemosphere.2015.11.090
6. Liu, J.; Wang, J.; Zhao, C.; Hay, A.G.; Xie, H.; Zhan, J. Triclosan removal in wetlands constructed with different aquatic plants. *Appl. Microbiol. Biotechnol.* **2016**, 100 (3), 1459-1467. DOI: https://doi.org/10.1007/s00253-015-7063-6
7. Li, J.; Zhou, Q.; Campos, L.C. Removal of selected emerging PPCP compounds using greater duckweed (*Spirodela polyrhiza*) based lab-scale free water constructed wetland. *Water Res*. **2017**, 126, 252-261. DOI: https://doi.org/10.1016/j.watres.2017.09.002
8. Anjos, M.L.; Isique, W.D.; Albertin, L.L.; Matsumoto, T.; Henares, M.N.P. Parabens Removal from Domestic Sewage by Free-Floating Aquatic Macrophytes. *Waste Biomass Valor.* **2019**, 10 (8), 2221–2226. DOI: https://doi.org/10.1007/s12649-018-0245-6
9. Carranza-Diaz, O.; Schultze-Nobre, L.; Moeder, M.; Nivala, J.; Kuschk, P.; Koeser, H. Removal of selected organic micropollutants in planted and unplanted pilot-scale horizontal flow constructed wetlands under conditions of high organic load. *Ecol. Eng.* **2014**, 71, 234-245. DOI: https://doi.org/10.1016/j.ecoleng.2014.07.048
10. Zhao, C.; Xie, H.; Xu, J.; Xu, X.; Zhang, J.; Hu, Z.; Liu, C.; Liang, S.; Wang, Q.; Wang, J. Bacterial community variation and microbial mechanism of triclosan (TCS) removal by constructed wetlands with different types of plants. *Sci. Total Environ.* **2015**, 505, 633-639. DOI: https://doi.org/10.1016/j.scitotenv.2014.10.053
11. Chen, Y.; Vymazal, J.; Březinová, T.; Koželuh, M.; Kule, L.; Huang, J.; Chen, Z. Occurrence, removal and environmental risk assessment of pharmaceuticals and personal care products in rural wastewater treatment wetlands. *Sci. Total Environ.* **2016**, 566-567, 1660-1669. DOI: https://doi.org/10.1016/j.scitotenv.2016.06.069

12. Herrera-Cardenas, J.; Navarro, A.E.; Torres, E. Effects of porous media, macrophyte type and hydraulic retention time on the removal of organic load and micropollutants in constructed wetlands. *J. Environ. Sci. Heal. A*. **2016**, 51 (5), 380-388. DOI: https://doi.org/10.1080/10934529.2015.1120512
13. Matamoros, V.; Rodríguez, Y.; Bayona, J.M. Mitigation of emerging contaminants by full-scale horizontal flow constructed wetlands fed with secondary treated wastewater. *Ecol. Eng.* **2017**, 99, 222-227. DOI: https://doi.org/10.1016/j.ecoleng.2016.11.054
14. Kahl, S.; Nivala, J.; Afferden, M.V.; Müller, R.A.; Reemtsma, T. Effect of design and operational conditions on the performance of subsurface flow treatment wetlands: Emerging organic contaminants as indicators. *Water Res*. **2017**, 125 (15), 490-500. DOI: https://doi.org/10.1016/j.watres.2017.09.004
15. Yi, X.; Tran, N.H.; Yin, T.; He, Y.; Gin, K.Y-H. Removal of selected PPCPs, EDCs, and antibiotic resistance genes in landfill leachate by a full-scale constructed wetlands system. *Water Res*. **2017**, 121, 46-60. DOI: https://doi.org/10.1016/j.watres.2017.05.008
16. Vymazal, J.; Březinová, T.D.; Koželuh, M.; Kule, L. Occurrence and removal of pharmaceuticals in four full-scale constructed wetlands in the Czech Republic – the first year of monitoring. *Ecol. Eng.* **2017**, 98, 354-364. DOI: https://doi.org/10.1016/j.ecoleng.2016.08.010
17. Salcedo, J.J.P.; Montes, G.H.E.; Frómeta, A.E.N.; Osorio, A.C.; Negrete, J.M. Removal of Organic Micropollutants from Riverine Waters using Constructed Wetlands: A Mesocosms Experiment. *Int. J. Appl. Eng. Res.* **2018**, 13 (22), 15740-15748.
18. Petrie, B.; Rood, S.; Smith, B.D.; Proctor, K.; Youdan, J.; Barden, R.; Kasprzyk-Hordern., B. Biotic phase micropollutant distribution in horizontal sub-surface flow constructed wetlands. *Sci. Total Environ.* **2018**, 630, 648-657. DOI: https://doi.org/10.1016/j.scitotenv.2018.02.242
19. Nivala, J.; Kahl, S.; Boog, J.; Afferden, M.; Reemtsma, T.; Müller, R.A. Dynamics of emerging organic contaminant removal in conventional and intensified subsurface flow treatment wetlands. *Sci. Total Environ.* **2019**, 649, 1144-1156. DOI: https://doi.org/10.1016/j.scitotenv.2018.08.339
20. Wang, Y.; Yin, T.; Kelly, B.C.; Gin, K.Y-H. Bioaccumulation behaviour of pharmaceuticals and personal care products in a constructed wetland. *Chemosphere* **2019**, 222, 275-285. DOI: https://doi.org/10.1016/j.chemosphere.2019.01.116
21. Matamoros, V.; Arias, C.; Brix, H.; Bayona, J.M. Removal of pharmaceuticals and personal care products (PPCPs) from urban wastewater in a pilot vertical flow constructed wetland and a sand filter. *Environ. Sci. Technol*. **2007**, 41 (23), 8171-8177. DOI: https://doi.org/10.1021/es071594+
22. Ávila, C.; Nivala, J.; Olsson, L.; Kassa, K.; Headley, T.; Mueller, R.A.; Bayona, J.M.; García, J. Emerging organic contaminants in vertical subsurface flow constructed wetlands: Influence of media size, loading frequency and use of active aeration. *Sci.Total Environ.* **2014**, 494-495, 211-217. DOI: https://doi.org/10.1016/j.scitotenv.2014.06.128
23. Francini, A.; Mariotti, L.; Gregorio, S.D.; Sebastiani, L.; Andreucci, A. Removal of micro-pollutants from urban wastewater by constructed wetlands with *Phragmites australis* and *Salix matsudana. Environ. Sci. Pollut. Res*. **2018**, 25 (36), 36474-36484. DOI: https://doi.org/10.1007/s11356-018-3582-x
24. Sgroi, M.; Pelissari, C.; Roccaro, P.; Sezerino, P.H.; García, J.; Vagliasindi, F.G.A.; Ávila, C. Removal of organic carbon, nitrogen, emerging contaminants and fluorescing organic matter in different

constructed wetland configurations. *Chem. Eng. J.* **2018**, 332, 619-627. DOI: https://doi.org/10.1016/j.cej.2017.09.122

25. Xie, H.; Yang, Y.; Liu, J.; Kang, Y.; Zhang, J.; Hu, Z.; Liang, S. Enhanced triclosan and nutrient removal performance in vertical up-flow constructed wetlands with manganese oxides. *Water Res.* **2018**, 143, 457-466. DOI: https://doi.org/10.1016/j.watres.2018.05.061
26. Button, M.; Cosway, K.; Sui, J.; Weber, K. Impacts and fate of triclosan and sulfamethoxazole in intensified re-circulating vertical flow constructed wetlands. *Sci. Total Environ.* **2019**, 649, 1017-1028. DOI: https://doi.org/10.1016/j.scitotenv.2018.08.395
27. Park, N.; Vanderford, B.J.; Snyder, S.A.; Sarp, S.; Kim, S.D.; Cho, J. Effective controls of micro-pollutants included in wastewater effluent using constructed wetlands under anoxic condition. *Ecol. Eng.* **2009**, 35 (3), 418-423. DOI: https://doi.org/10.1016/j.ecoleng.2008.10.004
28. Ávila, C.; Pedescoll, A.; Matamoros, V.; Bayona, J.M.; García, J. Capacity of a horizontal subsurface flow constructed wetland system for the removal of emerging pollutants: an injection experiment. *Chemosphere* **2010**, 81 (9), 1137-1142. DOI: https://doi.org/10.1016/j.chemosphere.2010.08.006
29. Hijosa-Valsero, M.; Matamoros, V.; Martin-Villacorta, J.; Becares, E.; Bayona, J.M. Assessment of full-scale natural systems for the removal of PPCPs from wastewater in small communities. *Water Res.* **2010**, 44 (5), 1429-1439. DOI: https://doi.org/10.1016/j.watres.2009.10.032
30. Hijosa-Valsero, M.; Matamoros, V.; Pedescoll, A.; Martín-Villacorta, J.; Bécares, E.; García, J.; Bayona, J.M. Evaluation of primary treatment and loading regimes in the removal of pharmaceuticals and personal care products from urban wastewaters by subsurface-flow constructed wetlands. *Int. J. Environ. An. Ch.* **2011**, 91 (7-8), 632-653. DOI: https://doi.org/10.1080/03067319.2010.526208
31. Matamoros, V.; Salvadó, V. Evaluation of the seasonal performance of a water reclamation pond-constructed wetland system for removing emerging contaminants. *Chemosphere* **2012**, 86 (2), 111-117. DOI: https://doi.org/10.1016/j.chemosphere.2011.09.020
32. Ávila, C.; Reyes, C.; Bayona, J.M.; García, J. Emerging organic contaminant removal depending on primary treatment and operational strategy in horizontal subsurface flow constructed wetlands: influence of redox. *Water Res.* **2013**, 47 (1), 315-325. DOI: https://doi.org/10.1016/j.watres.2012.10.005
33. Zhu, S.; Chen, H. The fate and risk of selected pharmaceutical and personal care products in wastewater treatment plants and a pilot-scale multistage constructed wetland system. *Environ. Sci. Pollut. Res.* **2014**, 21 (2), 1466-1479. DOI: https://doi.org/10.1007/s11356-013-2025-y
34. Ávila, C.; Matamoros, V.; Reyes-Contreras, C.; Piña, B.; Casado, M.; Mita, L.; Rivetti, C.; Barata, C.; García, J.; Bayona, J.M. Attenuation of emerging contaminants in a hybrid constructed wetland system under different hydraulic loading rates and their associated toxicological effects in wastewater. *Sci. Total Environ.* **2014**, 470-471, 1272-1280. DOI: https://doi.org/10.1016/j.scitotenv.2013.10.065
35. Ávila, C.; Bayona, J.M.; Martín, I.; Salas, J.J.; García, J. Emerging organic contaminant removal in a full-scale hybrid constructed wetland system for wastewater treatment and reuse. *Ecol. Eng.* **2015**, 80, 108-116. DOI: https://doi.org/10.1016/j.ecoleng.2014.07.056
36. Matamoros, V.; Rodríguez, Y.; Albaigés, J. A comparative assessment of intensive and extensive wastewater treatment technologies for removing emerging contaminants in small communities. *Water Res.* **2016**, 88, 777-785. DOI: https://doi.org/10.1016/j.watres.2015.10.058

37. Dai, Y.; Tao, R.; Tai, Y.; Tam, N.F.; Dan, A.; Yang, Y. Application of a full-scale newly developed stacked constructed wetland and an assembled bio-filter for reducing phenolic endocrine disrupting chemicals from secondary effluent. *Ecol. Eng.* **2017**, 99, 496-503. DOI: https://doi.org/10.1016/j.ecoleng.2016.11.007
38. Vystavna, Y.; Frkova, Z.; Marchand, L.; Vergeles, Y.; Stolberg, F. Removal efficiency of pharmaceuticals in a full scale constructed wetland in East Ukraine. *Ecol. Eng.* **2017**, 108 (Part A), 50-58. DOI: https://doi.org/10.1016/j.ecoleng.2017.08.009
39. Chen, J.; Liu, Y-S.; Deng, W-J.; Ying, G-G. Removal of steroid hormones and biocides from rural wastewater by an integrated constructed wetland. *Sci. Total Environ.* **2019**, 660, 358-365. DOI: https://doi.org/10.1016/j.scitotenv.2019.01.049
40. Matamoros, V.; Nguyen, L.X.; Arias, C.A.; Salvadó, V.; Brix, H. Evaluation of aquatic plants for removing polar microcontaminants: a microcosm experiment. *Chemosphere* **2012**, 88 (10), 1257-1264. DOI: https://doi.org/10.1016/j.chemosphere.2012.04.004
41. Burken, J.G.; Schnoor, J.L. Predictive relationships for uptake of organic contaminants by hybrid polar trees. *Environ. Sci. Technol.* **1998**, 32 (21), 3379-3385. DOI: https://doi.org/10.1021/es9706817
42. Reinhold, D.; Vishwanathan, S.; Park, J.J.; Oh, D.; Saunders, F.M. Assessment of plant-driven removal of emerging organic pollutants by duckweed. *Chemosphere* **2010**, 80 (7), 687-692. DOI: https://doi.org/10.1016/j.chemosphere.2010.05.045
43. Briggs, G.G.; Bromilow, R.H.; Evans, A.A. Relationships between lipophilicity and root uptake and translocation of non-ionized chemicals by barley. *Pestic. Sci.* **1983**, 13, 495-500. DOI: https://doi.org/10.1002/ps.2780130506
44. Alvarez, D.A.; Petty, J.D.; Huckins, J.N.; Jones-Lepp, T.L.; Getting, D.T.; Goddard, J.P.; Manahan, S.E. Development of a passive, in situ, integrative sampler for hydrophilic organic contaminants in aquatic environments. *Environ. Toxicol. Chem.* **2004**, 23 (7), 1640-1648. DOI: https://doi.org/10.1897/03-603
45. Le-Minh, N.; Khan, S.J.; Drewes, J.E.; Stuetz, R.M. Fate of antibiotics during municipal water recycling treatment processes. *Water Res.* **2010**, 44 (15), 4295-4323. DOI: https://doi.org/10.1016/j.watres.2010.06.020
46. Dan, A.; Yang, Y.; Dai, Y-n.; Chen, C-x.; Wang, S-y.; Tao, R. Removal and factors influencing removal of sulfonamides and trimethoprim from domestic sewage in constructed wetlands. *Bioresour. Technol.* **2013**, 146, 363-370. DOI: https://doi.org/10.1016/j.biortech.2013.07.050
47. Dietz, A.C.; Schnoor, J.L. Advances in phytoremediation. *Environ. Health Perspect*. **2001**, 109, 163-168. DOI: https://doi.org/10.1289/ehp.01109s1163
48. Pilon-Smits, E. Phytoremediation. *Ann. Rev. Plant Biol.* **2005**, 56, 15-39. DOI: doi:10.1146/annurev.arplant.56.032604.144214
49. Lee, S.; Kang, S.; Lim, J.; Huh, Y.J.; Kim, K.; Cho, J. Evaluating controllability of pharmaceuticals and metabolites in biologically engineered processes, using corresponding octanol-water distribution coefficient. *Ecol. Eng.* **2011**, 37, 1595-1600. DOI: https://doi.org/10.1016/j.ecoleng.2011.04.007
50. Zhang, D.Q.; Gersberg, R.M.; Zhu, J.; Hua, T.; Jinadasa, K.B.S.N.; Tan, S.K. Batch versus continuous feeding strategies for pharmaceutical removal by subsurface flow constructed wetland. *Environ. Pollut*. **2012**, 167, 124-131. DOI: https://doi.org/10.1016/j.envpol.2012.04.004

51. Piwoni, M.D.; Keeley, J.W. Ground Water Issue. *Basic Concepts of Contaminant Sorption at Hazardous Waste Sites*. U.S. Environmental Protection Agency, Washington, DC, EPA/540/4-90/053 (NTIS 91-191007), **1990**. URLs/Downloads: Ground Water Issue. BASIC CONCEPTS OF CONTAMINANT SORPTION AT HAZARDOUS WASTE SITES (Accessed: 28/01/19).
52. Yan, Q.; Feng, G.; Gao, X.; Sun, C.; Guo, J-S.; Zhu, Z. Removal of pharmaceutically active compounds (PhACs) and toxicological response of *Cyperus alternifolius* exposed to PhACs in microcosm constructed wetlands. *J. Hazard. Mater*. **2016**, 301, 566-575. DOI: https://doi.org/10.1016/j.jhazmat.2015.08.057
53. Jekel, M.; Dott, W.; Bergmann, A.; Dunnbier, U.; Gnirss, R.; Haist-Gulde, B.; Hamscher, G.; Letzel, M.; Licha, T.; Lyko, S.; Miehe, U.; Sacher, F.; Scheurer, M.; Schmidt, C.K.; Reemtsma, T.; Ruhl, A.S. Selection of organic process and source indicator substances for the anthropogenically influenced water cycle. *Chemosphere* **2015**, 125, 155-167. DOI: https://doi.org/10.1016/j.chemosphere.2014.12.025
54. Burke, V.; Greskowiak, J.; Grunenbaum, N.; Massmann, G. Redox and temperature dependent attenuation of twenty organic micropollutants - a systematic column study. *Water Environ. Res.* **2017**, 89 (2), 155-167. DOI: https://doi.org/10.2175/106143016X14609975746000
55. Castronovo, S.; Wick, A.; Scheurer, M.; Nödler, K.; Schulz, M.; Ternes, T.A. Biodegradation of the artificial sweetener acesulfame in biological wastewater treatment and sandfilters. *Water Res*. **2017**, 110, 342-353. DOI: https://doi.org/10.1016/j.watres.2016.11.041
56. Kahl, S.; Kleinsteuber, S.; Nivala, J.; van Afferden, M.; Müller, R.A.; Reemtsma, T. Emerging biodegradation of the previously persistent artificial sweetener acesulfame in biological wastewater treatment. *Environ. Sci. Technol*. **2018**, 52 (5), 2717-2725. DOI: https://doi.org/10.1021/acs.est.7b05619
57. Nuel, M.; Laurent, J.; Bois, P.; Heintz, D.; Wanko, A. Seasonal and ageing effect on the behaviour of 86 drugs in a full-scale surface treatment wetland: Removal efficiencies and distribution in plants and sediments. *Sci. Total Environ.* **2018**, 615, 1099-1109. DOI: https://doi.org/10.1016/j.scitotenv.2017.10.061
58. Meyer, J.; Bester, K. Organophosphate flame retardants and plasticisers in wastewater treatment plants. *J. Environ. Monit*. **2004**, 6, 599-605. DOI: https://doi.org/10.1039/B403206C
59. Beel, R.; Eversloh, C.L.; Ternes, T.A. Biotransformation of the UV-Filter Sulisobenzone: Challenges for the Identification of Transformation Products. *Environ. Sci. Technol*. **2013**, 47 (13), 6819-6828. DOI: https://doi.org/10.1021/es400451w
